# Reversing the Fold: Polyanionic Macrocycle Dissolves αA66–80 Crystallin Peptide Aggregates

**DOI:** 10.1021/acs.biomac.6c00441

**Published:** 2026-06-13

**Authors:** Frank Boateng Osei, Yuvraj Dangat, Roi Yasay, Josephine Esposto, Kwaku Twum, Robert J. Huber, Sanela Martic, Hedieh Torabifard, Ngong Kodiah Beyeh

**Affiliations:** † Department of Chemistry, 6918 Oakland University, 146 Library Drive, Rochester, Michigan 48309-4479, United States; ‡ Department of Chemistry and Biochemistry, 12335 The University of Texas at Dallas, 800 West Campbell Road, Richardson, Texas 75080-3021, United States; § Department of Biology, Environmental and Life Sciences Program, 6515 Trent University, 1154 Peterborough, Ontario K9L 0G2, Canada; ∥ Department of Pathology and Laboratory Medicine, Boston University School of Medicine, Boston, Massachusetts 02118, United States; ⊥ Department of Forensic Science, Trent University, 1154 Peterborough, Ontario K9L 0G2, Canada

## Abstract

Cataract is a leading cause of blindness worldwide, with no clear pharmacological agent for treatment. An octa-sulfonated polyanionic resorcinarene **MR-8S** was synthesized and investigated for its ability to inhibit the aggregation of αA66–80-Crystallin peptide, a key peptide of the αA-Crystallin protein involved in cataract formation. Nuclear magnetic resonance (NMR) and isothermal titration calorimetry (ITC) experiments revealed strong interactions between **MR-8S** and the nine amino acids that make up the αA66–80-Crystallin peptide as well as the peptide. ITC revealed dissociation constants ranging between 1 nm and 1000 μM. Fluorescence aggregation assays, dynamic light scattering (DLS) experiments, and transmission electron microscopy (TEM) graphs illustrated a concentration-dependent deaggregation ability of **MR-8S** toward αA66–80-Crystallin peptide in physiological solutions. At 1:1 equivalence of αA66–80-Crystallin and **MR-8S**, the average particle size of αA66–80 peptide aggregate dropped almost 9-fold (from 13293 ± 1072 d.nm to 1483 ± 15 d.nm), which was less than that determined for previously reported macrocycles with the same batch of peptide at a similar concentration. Evident from TEM imaging, varying ratios of **MR-8S** and αA66–80-Crystallin peptide produced fibrillar aggregates of the peptide when compared to pure αA66–80 Crystallin peptide. A concentration of 44 ± 1 μM of **MR-8S** was needed to break down 50% of αA66–80 peptide aggregates, which was significantly lower than reported IC_50_ values for two reported functionalized resorcinarenes and doubles that of a third macrocycle. Molecular dynamics (MD) simulations further showed that aggregation of the αA66–80 peptide was driven by core hydrophobic residues, which were effectively shielded by **MR-8S**, thereby inhibiting the formation of peptide aggregates. Among the four polyionic resorcinarenes tested, **MR-8S** displayed the strongest deaggregation effects, highlighting its potential as a molecular scaffold for the development of anticataract therapeutics.

## Graphical abstract

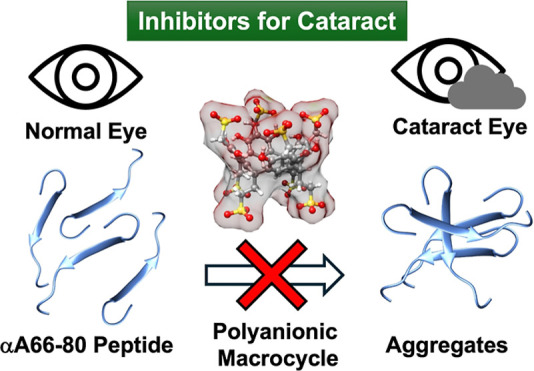

## 1 Introduction

Cataracts are the leading cause of blindness worldwide^1–7^ with no absolute cure apart from an expensive and uncomfortable surgery, which may be inaccessible in many developing countries.^8–10^ A noninvasive treatment option will be ideal, especially in a world of an increasingly aging population. Cataracts develop when αA-Crystallin, a structural and functional protein, aggregates with other ocular proteins, leading to blockade of the eye lens, further deteriorating to blindness.^11–14^ During aging, the αA-Crystallin protein concentration in the eye lens diminishes substantially, leading to the generation of low molecular weight peptide (LMW) fragments.^15–17^ The LMW peptide fragments are located in aggregates of cataract lenses, suggesting their critical involvement in the disease process.^15,18–20^ Notably, elevated levels of the αA66–80 peptide fragment of αA-Crystallin, along with other fragments, were detected at elevated levels in cataract eye lenses.^21^ The αA-Crystallin peptide sequence has also been examined for its propensity to aggregate and generate reactive oxygen species.^22^ Aggregation of the αA66–80 Crystallin peptide fragment has emerged as a compelling therapeutic target, and multiple small molecules, including aspirin, flavonoids, and orange G, have been systematically investigated for their potential to inhibit this process.^22,23^ Lanosterol has also been investigated in managing cataracts but produced suboptimal effects.^24,25^ The ineffectiveness of lanosterol was attributed to poor solubility in water and limited penetration into the eye lens to affect dissolution of existing protein aggregates.^26,27^

Macrocyclic hosts, such as resorcinarenes, have been employed in studying various biological processes and systems due to their ability to bind and alter the properties of different biological guests.^28–31^ These macrocycles boast of a cheaper and relatively straightforward method of synthesis, even in larger quantities.^29,32^ Our previous study explored the effects of three resorcinarene macrocycles, i.e., upper-rim tetrasulfonated resorcinarene (**UR-4S**), lower-rim tetrasulfonated resorcinarene (**LR-4S**), and upper-rim *N*-benzyl ammonium resorcinarene chloride salt (**UR-4A**), on the deaggregation of αA66–80 Crystallin peptide as a potential model in targeting the deaggregation of αA-Crystallin protein (Figure 1).^7^ The study revealed that both **UR-4S** and **LR-4S** attached to the αA66–80 Crystallin peptide at different locations on the peptide, leading to differing deaggregation effects on the αA66–80 Crystallin peptide.^7^ Given the characteristics of the binding properties of these resorcinarenes and their potential impact on αA66–80 Crystallin deaggregation, we hypothesize that an octasulfonated resorcinarene (**MR-8S**) that combines the features of both **UR-4S** and **LR-4S**, i.e., with a total of eight sulfonate groups, four on the upper and four on the lower rims, will substantially enhance the binding to αA66–80 Crystallin peptide, leading to a more potent and efficient deaggregation process. **MR-8S** has an established safety profile^29^ and provides a highly water-soluble formulation, allowing for greater potential penetration into the lens to facilitate adequate dissolution of αA66–80 Crystallin aggregates. This would be a basis for developing a less expensive, effective, noninvasive, and safer alternative for cataract treatment. We hereby investigate the effects and mechanism of interaction of an octa-sulfonated resorcinarene, **MR-8S,** on the deaggregation of α66–80-Crystallin peptide. The deaggregation studies were conducted through a series of analytical processes, including dynamic light scattering (DLS), fluorescence spectroscopy, and transmission electron microscopy (TEM). Nuclear magnetic resonance (NMR) and isothermal titration calorimetry (ITC) provide detailed analysis of the interactions with the individual amino acids on the α66–80-Crystallin peptide as well as the peptide. To complement our experimental findings, we conducted molecular dynamics (MD) simulations aiming at atomic-level insights into the aggregation behavior of the αA66–80 Crystallin peptide and its inhibition by these resorcinarenes. While experimental assays established the inhibitory potential of **MR-8S**, MD simulations elucidated the structural determinants of peptide self-association and the molecular interactions through which resorcinarenes modulate this process. By integrating computational and experimental approaches, this study provides a comprehensive understanding of the αA66–80 peptide aggregation mechanism and highlights the critical role of **MR-8S** in preventing the formation of aggregation-prone conformations.

**Figure 1:**
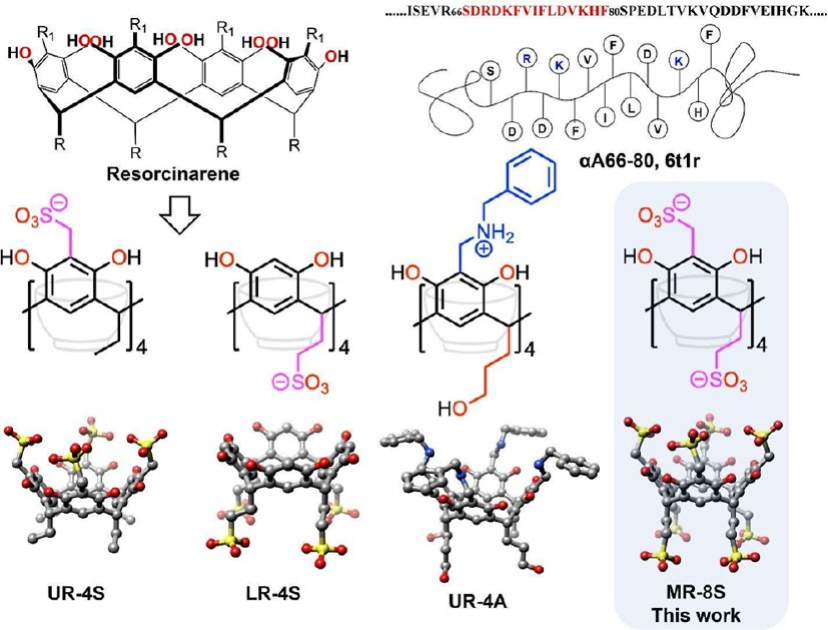
Structure of the αA66–80 Crystallin peptide chain and DFT-optimized geometries of functionalized polyionic macrocycles: upper-rim tetrasulfonated resorcinarene (**UR-4S**), lower-rim tetrasulfonated resorcinarene (**LR-4S**), upper-rim *N*-benzyl ammonium resorcinarene chloride (**UR-4A**), and mixed-rim octasulfonated resorcinarene (**MR-8S**). Macrocycle structures were optimized at the B3LYP^33^/6–31G­(d,p)^34,35^ level of theory. Atom color scheme: carbon, gray; nitrogen, blue; oxygen, red; sulfur, yellow; hydrogen atoms are omitted for clarity.

## 2 Experimental Section

### 2.1 Synthesis

A two-phase mixture of 2-(2-bromoethyl)-1,3-dioxane (4.0 g, 20 mmol), and an aqueous solution (20 mL) of Na_2_SO_3_ (5.0 g, 40 mmol) was stirred at 100 °C for 24 h. Water (20 mL) was added to the resulting homogeneous solution. The mixture was washed with ether (40 mL x2) to get rid of unreacted 2-(2-bromoethyl)-1,3-dioxane. Ethanol (40 mL), resorcinol (4.0 g, 36 mmol), and concentrated HCI (6 mL) were added to the mixture. The mixture was stirred under nitrogen at 100 °C for 24 h. The solvent was evaporated, and the residue was taken up in water (60 mL) and dialyzed three times against water (2 L) using a dialysis membrane having a transport critical molecular weight of 1000 (Spectra/Por membrane MWCO 1000) to remove inorganic salts. Most of the water was removed in vacuo, and the residue was triturated from methanol to give the lower rim tetrasulfonated resorcinarene (**LR-4S**). A mixture of **LR-4S** (0.01 mol), a solution of 37% formaldehyde (0.01 mol), and sodium sulfite (0.01M) in H_2_O (30 mL) was stirred and heated at 90–95 °C for 4 h. Dilute hydrochloric acid was added after cooling until pH 7, then methanol (50 mL or more) was added to precipitate the product **MR-8S**. Detailed synthetic schemes and spectra are reported in the Supporting Information

### 2.2 Mass Spectrometry

The mass spectrometric studies were performed with a Thermo Scientific Q-Exactive Plus Hybrid Quadrupole-Orbitrap mass spectrometer equipped with a heated electrospray ionization II (HESI-II) probe. The instrument was run in both positive and negative ion modes for all experiments, and was performed under low temperature conditions to stabilize the complexes formed (40 °C). The parameters of the ion source and fragmentation (MS/MS) were optimized for the maximum abundance of the ions. Detailed mass spectrometric procedures, spectra, and analysis are reported in the Supporting Information

### 2.3 Dynamic Light Scattering

To assess the particle size distribution of the αA66–80-Crystallin peptide with **MR-8S**, pure solutions of α66–80-Crystallin and the mixtures at 0.2, 0.4, 0.6, 0.8, 1.0, 2.0, 5.0, and 10.0 ratios were incubated at 37 °C in 10 mM Tris buffer (pH 7.4) for 7 days. One mL of each solution was pipetted into a cuvette and measured using the Malvern zetasizer. Each experiment was done in triplicate. The size distribution and Z-averages were obtained and analyzed. Detailed Z-averages and spectra are reported in the Supporting Information

### 2.4 Proteostat Fluorescence Aggregation Assay

αA66–80-Crystallin peptide–receptor mixtures were prepared by adding 1 mL of a freshly prepared 536 μM Crystallin peptide solution in 10 mM Tris buffer to a series of samples. Subsequently, 1 mL of a concentrated **MR-8S** solution was added to achieve **MR-8S**/peptide molar ratios of 0.2:1, 0.4:1, 0.6:1, 0.8:1, 1:1, 5:1, and 10:1. The resulting mixtures were incubated at 37 °C for 7 days. A control sample containing the peptide alone was incubated under identical conditions. Following incubation, 50 μL of ProteoStat dye was added to 800 μL of each sample. Prior to transferring the samples to the microplate wells, positive and negative controls were prepared and added to the plate to ensure sample integrity and to verify that free ProteoStat dye contributed minimally to the measured fluorescence signal. Fluorescence measurements were performed at 37 °C using excitation and emission wavelengths of 550 and 600 nm, respectively, to monitor fibril and filament formation. All measurements were conducted in triplicate. A calibration curve of the logarithm of concentration versus percentage inhibition of aggregation was generated, and the IC50 value was determined from the resulting plot. The IC50 plots are reported in the Supporting Information

### 2.5 Transmission Electron Microscopy

αA66–80 Crystallin peptide was mixed with **MR-8S** in a 1:1 molar ratio to a final equimolar concentration of 5 mM in DI water. Pure Crystallin and pure **MR-8S** were also prepared individually to final concentrations of 5 mM in DI water. Samples were incubated for 7 days at 37 °C. Samples for αA66–80 Crystallin peptide, **MR-8S**, and the αA66–80 Crystallin peptide:**MR-8S** mix were also freshly made on day 7 to compare with the aged samples. Ten μL aliquots were taken from each sample, loaded onto a Formvar-carbon-coated 200 mesh nickel grids, and allowed to absorb for 15 min in ambient light. The grids were washed with 10 μL of DI water and blotted dry with filter paper. Five μL of 2% glutaraldehyde was then loaded onto each grid for 5 min, blotted dry, and washed with DI water. Each grid was then stained with 5 μL of 1% uranyl acetate for 5 min, blotted dry, and washed a final time with DI water. Images were acquired from the TEM using a magnification range of 3000–10,000*x*. Different pictures of the grids are reported in the Supporting Information

### 2.6 NMR Spectroscopy

Stock solutions of **MR-8S** (5 mM) and the amino acids or αA66–80 Crystallin peptide (5 mM) were prepared in D_2_O. For individual sample measurements, 250 μL of the 5 mM stock solution was transferred to an NMR tube and diluted with 250 μL of D_2_O, yielding a final concentration of 2.5 mM. To investigate potential binding interactions, 250 μL of the 5 mM **MR-8S** solution was mixed with 250 μL of a 5 mM amino acid solution in an NMR tube, resulting in a 1:1 equimolar mixture containing 2.5 mM of each component. This procedure was repeated for each amino acid studied. ^1^H NMR spectra were recorded on a 400 MHz Bruker spectrometer. Detailed NMR spectra are reported in the Supporting Information

### 2.7 Isothermal Titration Calorimetry

The ITC experiments were performed by loading the sample cell with a 1 mM solution of **MR-8S** and the injection syringe with a 10 mM solution of the amino acid or the αA66–80 Crystallin peptide . Titrations were conducted at 310 K using a computer-controlled automated injector. Blank titrations of the amino acid or αA66–80 Crystallin peptide solution into 10 mM Tris buffer were also performed and subtracted from the corresponding experimental titrations to correct for heats of dilution. Binding isotherms and thermodynamic parameters, including the association constant (Ka), enthalpy change (Δ*H*), and entropy change (Δ*S*), were obtained by fitting the data to single-site and multiple-site binding models using NanoAnalyze software. The Gibbs free energy change (Δ*G*) was subsequently calculated at 310 K and reported. Detailed ITC plots and fittings are reported in the Supporting Information

### 2.8 Molecular Dynamics Simulations

All simulations were performed using the pmemd.cuda^36,37^ implementation of the AMBER24 ^38,39^ software package. To ensure proper relaxation, the systems underwent a multistage explicit solvent equilibration protocol. First, the added water molecules were minimized for 5000 steps (steepest descent followed by conjugate gradient) while restraining the solute with a force constant of 100 kcal mol^–1^ Å^–2^. This was followed by three rounds of MD at constant pressure and 298 K with progressively decreasing restraints. In the first round, the system was heated from 100 to 298 K over 1 ns with peptide and resorcinarene restrained at 100 kcal mol^–1^ Å^–2^. In the second round, box density was equilibrated for 1 ns at 298 K while restraining the peptide backbone and ions (100 kcal mol^–1^ Å^–2^), allowing other atoms to relax. In the third round, the system was equilibrated for 1 ns with backbone restraints reduced to 10 kcal mol^–1^ Å^–2^. The system was then minimized for 1000 steps with 10 kcal mol^–1^ Å^–2^ backbone restraints, followed by three additional 1 ns equilibration rounds with restraints of 10, 1.0, and 0.1 kcal mol^–1^ Å^–2^, respectively. A final 1 ns equilibration at constant pressure and 298 K with no restraints allowed the peptide–ligand complex to fully relax. The fully relaxed structure was equilibrated through a 20 ns production run using a 2 fs time step, with all bonds involving hydrogen atoms constrained via the SHAKE^37^ algorithm. Long-range electrostatic interactions were treated using a 9 Å cutoff, and simulations were carried out in the NPT ensemble at 298 K, employing a Langevin thermostat^40^ and a Berendsen barostat.^41^ Following this, the production run was extended to either 100 or 300 ns with three independent replicas, which served as the final production simulations. The analysis were carried out on the final 100 or 300 ns of the trajectories, and residue-wise interaction energies were determined using the linear interaction energy (lie) modules available in cpptraj^42^ module of AMBER24.^38,39^ To enable a comparable assessment of core shielding using SASA, the uncomplexed αA66–80 peptide monomer was simulated using the same MD protocol in the replicates of three. The detailed theoretical and computational calculations are reported in the Supporting Information

## 3 Results and Discussion

The αA66–80 Crystallin peptide was purchased from GenScript and used without further purification. The **MR-8S** receptor was synthesized according to a reported procedure.^29^ The macrocycle **MR-8S** was decorated with four sulfonate groups at both the upper rim and the lower rim. Briefly, first, the lower rim sulfonated resorcinarene **LR-4S** was obtained from reacting resorcinol with 2-(2-bromoethyl)-1,3-dioxane and Na_2_SO_3_. The **LR-4S** was then reacted with formaldehyde and Na_2_SO_3_ under reflux conditions to obtain the mixed-rim octasulfonated resorcinarene **MR-8S** (Figures S1–S3). **LR-4S**, **UR-4S**, and **UR-4A** were synthesized according to reported procedures.^7^

### 3.1 Mass Spectrometry

The stability of the **MR-8S** and αA66–80 Crystallin peptide was tested in the gas phase using an ESI buffer, which is a solution used in electrospray ionization mass spectrometry (ESI-MS) to maintain sample stability, solubility, and pH during analysis. ESI-MS, a soft ionization method, was used to characterize the **MR-8S** and αA66–80 Crystallin peptide using freshly made sample solutions, as well as an equimolar mixture of both (1:1). The mass spectrum of the peptide shows the [M+3H]^3+^ peak at *m*/*z* 623.0013, associated with the triply charged peptide in the positive ion mode (Figure S4). [M + H]^2–^ peak at *m*/*z* 931.9921 associated with a doubly deprotonated peptide was observed in the negative ion mode (Figure S5). The mass spectra for the receptor **MR**-**8S** show peaks at *m*/*z* 430.9914 [M+8H-8Na]^3–^, 438.3165 [M+7H-7Na]^3–^, 445.6438 [M+6H-6Na]^3–^, and 452.9711 [M+5H-5Na]^3–^, respectively (Figure S6). The MS analysis of an equimolar mixture of the showed small signals corresponding to the receptor + peptide complex, such as *m*/*z* 1053.3174 [**MR-8S**+αA66–80 + 10H-8Na]^3–^ (Figure S7).

### 3.2 Dynamic Light Scattering

Dynamic Light Scattering experiments are used to study the particle size distributions of α66–80-Crystallin in isolation and in mixtures with varying concentrations of **MR-8S**. This method affords the study of aggregate formation without the inclusion of a dye in solution and has been useful for measuring the size distribution of small particles, peptides, cells, carbohydrates, nanoparticles, and polymers in solution.^7,29,30,43^ The macrocycle’s ability to bind and complex α66–80-Crystallin peptide was studied by following the count rate with DLS. Measurements were carried out on increasing macrocycle: peptide molar ratios (0.0, 0.2, 0.4, 0.6, 0.8, 1.0, 2.0, 5.0, and 10.0:1) after 7 days of incubation at 37 °C. The results were compared to the αA66–80-Crystallin peptide without the macrocycle. Concentration of **MR-8S** as low as a 0.2:1 ratio shifted the particle sizes more toward smaller sizes compared to pure αA66–80-Crystallin peptide (Figure 2). In the incubations of pure αA66–80 Crystallin peptide without any macrocycle, the Z-average size was 13293 ± 1072 d.nm after 7 days of incubation in 10 mM Tris buffer. This value substantially reduced as the concentration of **MR-8S** increased. The average sizes from triplicate measurements of a 1:1 concentration ratio of macrocycles **LR-4S**, **UR-4S**, and **UR-4A**/αA66–80 Crystallin on the same batch of peptide were 5681 ± 1184, 4998 ± 596, and 2120 ± 101 d.nm, respectively, in 10 mM Tris buffer. Interestingly, a 1:1 mixture of **MR-8S** and αA66–80 Crystallin peptide gave an average size of 1483 ± 15 d.nm and 2118 ± 286 d.nm in Tris buffer and 100% aqueous humor, respectively. The size (Z-) average in d.nm of triplicate measurements of the different concentration ratios reflects a similar inhibitory effect of **MR-8S** against aggregation of the αA66–80 Crystallin peptide­(Figures 2, and S8, S9; Tables S1 and S2). From these measurements, it can be concluded that aggregation inhibition of αA66–80 Crystallin peptide is evident in the presence of **MR-8S** and substantially more when compared to the reported **LR-4S**, **UR-4S**, and **UR-4A**.

**Figure 2:**
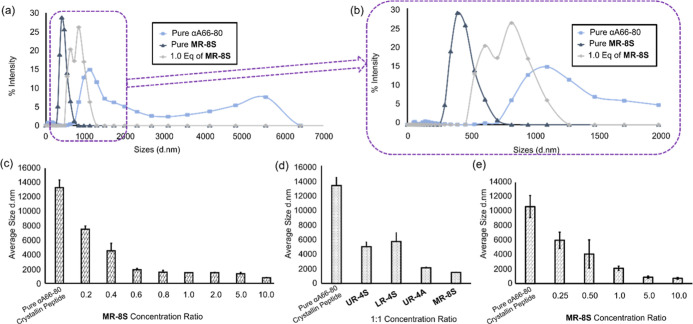
(a) Graph showing percent intensities of Z-average sizes in solution of pure αA66–80 Crystallin peptide, pure receptor **MR-8S**, and mixtures of **MR-8S**+αA66–80. (b) Inset of graph (a) highlighting the effect of **MR-8S**. (c) Bar chart showing the average size distribution of the assemblies formed with different ratios of up to 10 equiv of **MR-8S** mixed with the αA66–80 peptide in Tris buffer. (d) Bar chart showing the superior deaggregation potential of **MR-8S** when compared with a 1:1 equiv mixture of reported macrocycles **LR-4S**, **UR-4S**, and **UR-4A**. (e) Bar chart showing the average size distribution of the assemblies formed with different ratios of up to 10 equiv of **MR-8S** mixed with the αA66–80 peptide in 100% aqueous humor. All experiments were done in triplicate.

### 3.3 Fluorescence Aggregation Assay

Fluorescence aggregation assays are used to detect the presence of protein aggregates in varying concentrations of **MR-8S** using the Proteostat detection dye, as **MR-8S** is a weak fluorescent compound. In principle, this dye intercalates into quaternary protein structures, which are typical of aggregated β-pleated sheet peptides and proteins, to enhance fluorescence emission.^44,45^ The dye has been reliably used as an indicator of β-pleated sheet formation during αA66–80 Crystallin peptide aggregation.^7,46^ An inverse relationship between fluorescence intensity and resorcinarene concentration would suggest that the resorcinarene disrupts peptide aggregation. Results indicate a positive correlation between **MR-8S** concentration and deaggregation. Pure αA66–80 Crystallin peptide aggregation resulted in structural assemblies that yielded a high fluorescence intensity. To assess the level of aggregation of the αA66–80 Crystallin peptide with **MR-8S**, the mixtures of macrocycle/peptide ratios at 0.2, 0.4, 0.6, 0.8, 1.0, 2.0, 5.0, and 10.0 were incubated at 37 °C in 10 mM Tris buffer (pH 7.4) for 7 days. Comparatively, in the presence of **MR-8S**, all ratios showed a concentration-dependent reduction in the extent of peptide aggregation. A 1:1 ratio of **MR-8S** and αA66–80 Crystallin peptide resulted in approximately 5-fold reduction in fluorescence intensity compared to pure α66–80-Crystallin peptide, with approximately 80% reduction in aggregation. A near complete reduction is achievable above a 10:1 ratio of **MR-8S** and αA66–80-Crystallin peptide (Figure 3).

**Figure 3:**
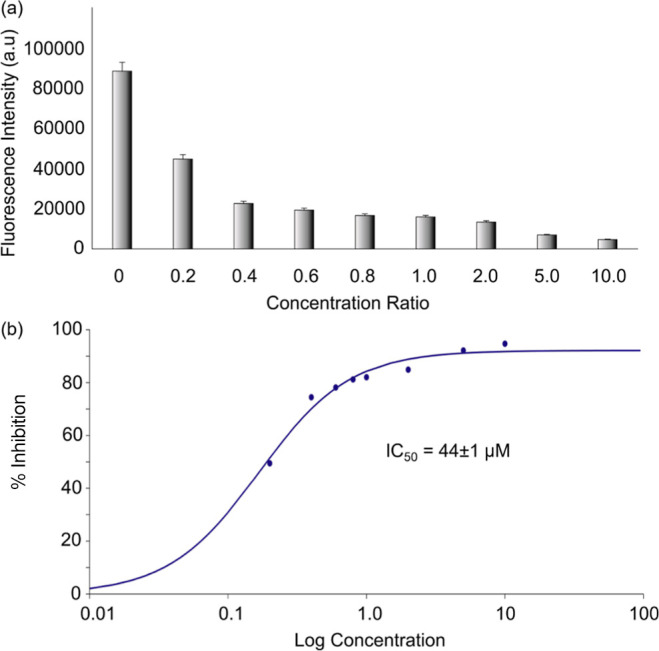
Figure 3. (a) Charts of fluorescence aggregation intensities at different concentrations of **MR-8S** with αA66–80-Crystallin peptide. (b) Percent inhibition of αA66–80-Crystallin peptide aggregates in increasing concentrations of **MR-8S** in 10 mM Tris buffer. All experiments were done in triplicate.

Positive and negative controls were done to rule out any inconsistent fluorescence contributions from the Proteostat dye. To make sure, (a) each freshly prepared dye solution is effective, (b) there is little to no fluorescence contributions from free dye solutions. Results show high and minimal fluorescence intensities from the proprietary positive and negative controls, respectively. The normalized inhibitory response was also used to derive the IC_50_, which refers to the concentration of **MR-8S** required to cause 50% deaggregation, with a calculated value of 44 ± 1 μM (Figures 3, and S10). This value is substantially smaller than those reported for **UR-4S** and **UR-4A** (203 and 418 M, respectively).^7^ However, its inhibitory potency relative to **LR-4S** (IC50 = 89 μM) is only ∼2-fold greater, suggesting that the performance of the inhibitor against **LR-4S** may not be as pronounced as the other two resorcinarenes. These results indicate that **MR-8S** has a strong inhibitory effect on αA66–80 Crystallin peptide, also aided by its high aqueous solubility. These fluorescence results also support the DLS results showing a positive correlation between the concentration of **MR-8S** and the deaggregation of α66–80-Crystallin peptide.

### 3.4 Transmission Electron Microscopy

TEM was used to visualize and further analyze αA66–80 Crystallin peptide aggregates in the presence of increasing concentrations of **MR-8S**, after aging for 7 days at 37 °C. Samples from the freshly prepared solution were imaged using a magnification range of 3000–10,000x. The imaging results revealed clear morphological differences among the Crystallin peptide samples in the absence and presence of **MR-8S** (Figures 4, and S11). Figure 4A (middle) demonstrates the morphology of the αA66–80 Crystallin peptide alone, which predominantly formed extensive fibrillar networks that were similar to amyloid-like tendrils or clumps.^47,48^ Here, the fibrils appeared long, unbranched, and densely intertwined, spanning several micrometers in length. This was consistent with the results from the fluorescence and DLS experiments. By contrast, Figure 4A (top) shows **MR-8S** structures alone, which lacked fibrillar or filamentous structures but contained sparse, diffuse particles observed across the grids. The addition of **MR-8S** to the Crystallin peptide (Figure 4A, bottom) showed good potential to disrupt the αA66–80 Crystallin peptide aggregation, as it showed fewer fibrillar aggregates of the peptide when compared to pure αA66–80 Crystallin peptide, suggesting an inhibitory potential for **MR-8S** toward the aggregation of α66–80-Crystallin peptide.^49^ The fibrillar organization was disrupted compared to pure peptide and resulted in a mixture of fragmented, short fibrils and amorphous, electron-dense aggregates. These darker clustered regions suggest that **MR-8S** interactions with the peptide induce partial mediation of the structure through either direct macrocycle-mediated interaction (impacting the peptide’s ability to self-assemble into extended fibrils) or could stabilize nonfibrillar intermediates of the peptide.

**Figure 4:**
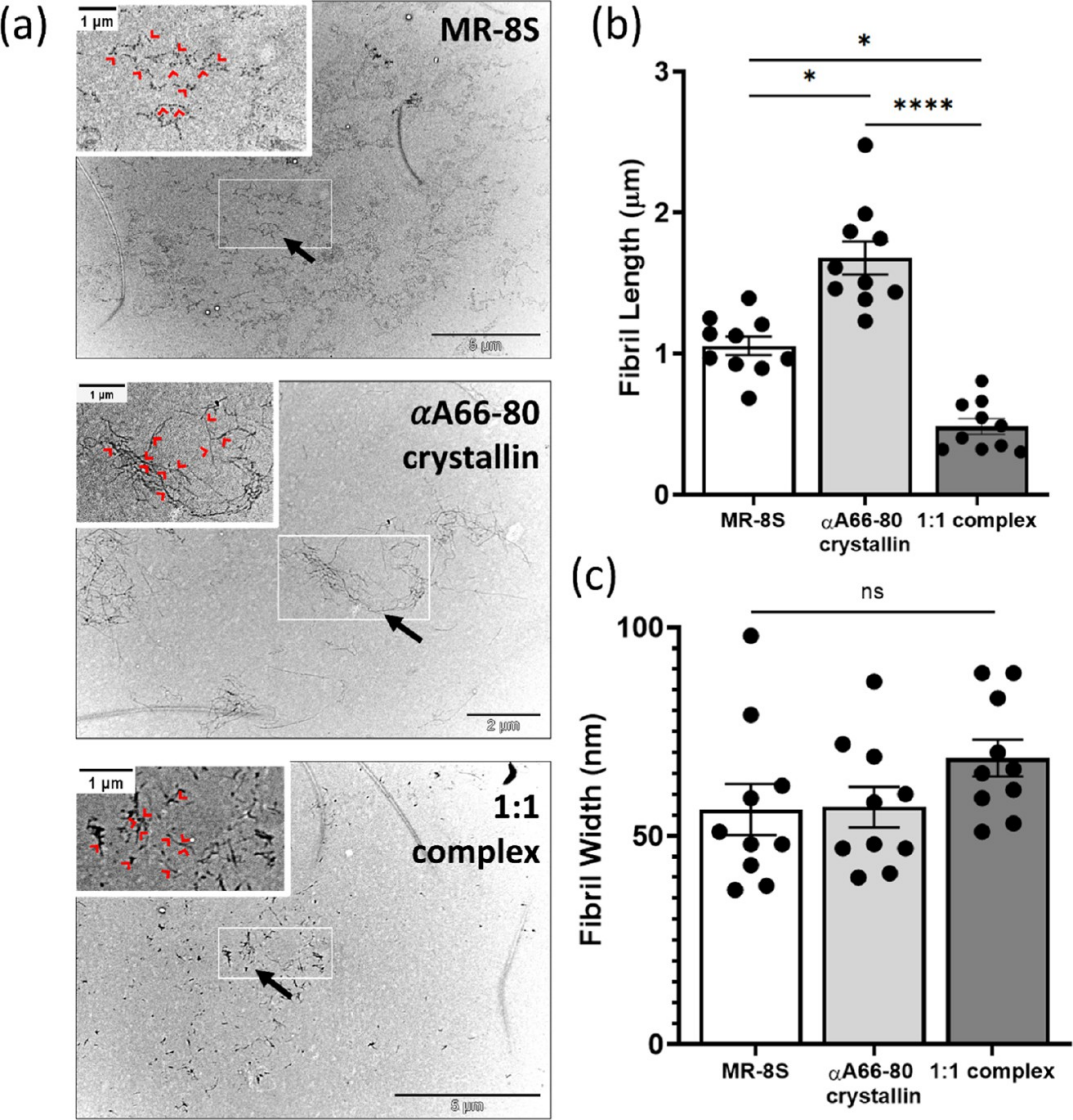
TEM images of αA66–80 Crystallin peptide aged (*t* = 7d) (a) **MR-8S**, αA66–80 Crystallin peptide alone, and αA66–80 Crystallin peptide:**MR-8S** complexation (1:1 molar concentration ratio). Black arrows: showing morphological structures within each TEM image. Red arrows: specific fibrils used in width and length analysis. (b) Fibril length (μm) and (c) fibril width (nm) of aggregates in each respective sample, along with statistical significance. Data are presented as the mean ± SEM (*n* = 10). Stats: Kruskal–Wallis test with Dunn’s multiple comparisons posthoc test *p < 0.05, ****p < 0.0001, ns = not significant.

### 3.5 Nuclear Magnetic Resonance

^1^H NMR is useful as the chemical and electronic environments of the protons are affected in macrocycle–substrate interactions. To gain some insight into the potential interactions between the **MR-8S** and the αA66–80 Crystallin peptide, we investigated the binding ability of **MR-8S** toward all the individual amino acids within the αA66–80 Crystallin peptide using ^1^H NMR experiments. This qualitative process was achieved by monitoring the complexation-induced ^1^H NMR chemical shift changes of the **MR-8S**-amino acid mixtures and comparing them to those of the pure **MR-8S** and the amino acids. Complexation of guests within the resorcinarene cavity is commonly signaled by guest resonance shielding and broadening, whereas hydrogen-bonding interactions are characterized by downfield chemical shift changes. The ^1^H NMR spectra of the mixtures in D_2_O display only a single set of signals, indicating that the complexes are in a rapid dynamic equilibrium with their free components. The ^1^H NMR spectra depict varying degrees of shielding of amino acid protons ranging between 0.04 and 0.86 ppm (Figures 5, and S6–S8), consistent with in-cavity-complexation.^50–52^ Shielding from 0.04 to 0.29 ppm was observed with all the neutral amino acids (Phe, Ile, Leu, and Val) that form the hydrophobic section of the αA66–80 Crystallin peptide (Figure 5). This shows the amino acids reside in the hydrophobic cavity of **MR-8S**. Significant shielding from 0.05 to 0.86 ppm was observed with the cationic amino acids (Lys, Arg, and His, Figure S12). This suggests strong interactions between the cationic amino acids and the sulfonate groups on **MR-8S**. The degree of shielding also suggests a potential interaction with the internal aromatic cavity of **MR-8S** through likely cation–π interactions. Interestingly, shift changes of up to 0.18 and 0.20 ppm were also observed between **MR-8S** and negatively charged Asp and polar Ser, respectively (Figures S13 and S14). In principle, **MR-8S** interacted to some degree with all the amino acids on the α66–80-Crystallin peptide. This is markedly different from the reported **LR-4S**, **UR-4S**, and **UR-4A,** which only show a preference for mainly the cationic amino acids Lys and Arg, with minor changes between **UR-4A** and Asp.^7^

**Figure 5:**
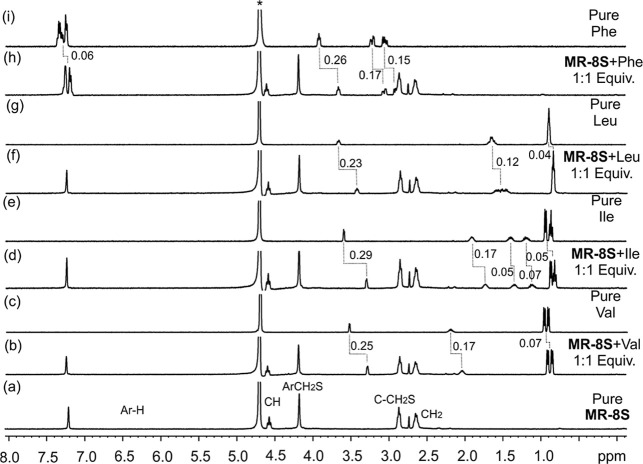
Sections of the ^1^H NMR spectra in D_2_O at 298 K of receptor **MR-8S** and several neutral amino acids of the αA66–80 Crystallin peptide. Pure samples: (a) **MR-8S**, (c) Val, (e) Ile, (g) Leu, and (h) Phe. Equimolar mixtures of: (b) **MR-8S** + Val, (d) **MR-8S** + Ile, (f) **MR-8S** + Leu, and (i) **MR-8S** + Phe. The dashed lines indicate the signal changes in ppm. The star (*) represents the residual D_2_O solvent.

We then investigated the binding ability of **MR-8S** toward the αA66–80 Crystallin peptide via ^1^H NMR. The ^1^H NMR spectra depict varying degrees of shielding of some of the amino acid protons on the αA66–80 Crystallin peptide backbone. While it is challenging to pinpoint the exact amino acid, we identify the peak ∼8.5 ppm to correspond to histidine protons and ∼7.2 ppm to correspond to phenylalanine (Figures 6, and S15). These peaks show intense shielding of ∼ 0.19 ppm and ∼ 0.16 ppm, respectively, consistent with in-cavity-complexation. Other signals show both shielding and deshielding, which clearly confirms that the **MR-8S** macrocycle interacts with the αA66–80 Crystallin peptide.

**Figure 6:**
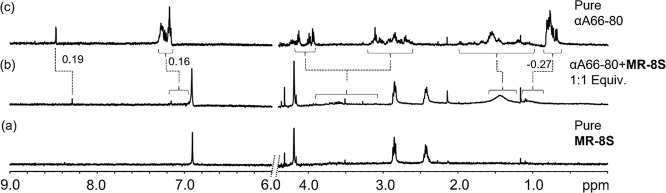
Sections of the ^1^H NMR spectra in D_2_O at 298 K of receptor **MR-8S** and αA66–80 Crystallin peptide. Pure samples: (a) **MR-8S**, (c) αA66–80 peptide. (b) Equimolar mixture of **MR-8S**+αA66–80 peptide. The dashed lines indicate the signal changes in ppm.

### 3.6 Isothermal Titration Calorimetry

ITC experiments are useful for quantifying the binding process and gaining insights into the thermodynamic parameters involved in receptor–substrate interactions, such as binding constants (K), change in enthalpy (Δ*H*), change in entropy (Δ*S*), and Gibbs free energy (Δ*G*).^53–55^ ITC isotherms (Figures S16–S24) showed multiple binding sites, and this is possible for the multiple binding moieties available on the amino acids to interact with **MR-8S**. Complexations were both enthalpically and entropically favorable at 37 °C. The octa-sulfonated macrocycle **MR-8S** showed substantially stronger binding toward all the amino acids as compared to the reported tetrasulfonated analogues **LR-4S** and **UR-4S**, and **UA-4S**.^7^ The dissociation constants (K_d_) reveal stronger binding for the cationic and neutral amino acids with hydrophobic side chains (Table 1), similar to results obtained from ^1^H NMR. Though interaction with the free amino acids in solution cannot be directly assumed to be the same when they are attached to a peptide backbone, these results do show a correlation of possible interaction between the **MR-8S** and the α66–80-Crystallin peptide. The thermodynamics of binding between the **MR-8S** macrocycle and the α66–80-Crystallin peptide were also investigated via ITC. The isotherm was fitted to a two-site sequential model with the dissociation constants (K_d_) revealing stronger binding to the α66–80-Crystallin peptide in the low micromolar range (Table 1, and Figure S25).

**Table 1:** Thermodynamic Parameters Associated with the Binding of **MR-8S** to the Amino Acids of α66-80-Crystallin Peptide and Also with the Peptide at 310 K

system	*K*_d1_ (μM)	*K*_d2_ (μM)	Δ*H*_*1*_ (kJ/mol)	Δ*H*_*2*_ (kJ/mol)	*T*Δ*S*_*1*_ (kJ/mol)	*T*Δ*S*_*2*_ (kJ/mol)	Δ*G*_*1*_ (kJ/mol)	Δ*G*_*2*_ (kJ/mol)
**MR-8S** + Arg	0.001	1000	–0.07	–200.00	53.23	–190.15	–53.29	–9.85
**MR-8S** + Asp	39.97	0.001	–7.08	–0.93	18.74	52.42	–25.82	–53.35
**MR-8S** + His	0.39	1000	0.04	11.93	38.07	30.21	–38.03	–18.28
**MR-8S** + Ile	1000	0.001	1.40	–0.06	19.26	52.95	–17.86	–53.01
**MR-8S** + Leu	1000	0.001	9.65	–0.09	27.84	52.67	–18.19	–52.50
**MR-8S** + Lys	0.001	1000	–0.15	–200.00	53.26	–190.15	–53.41	–9.85
**MR-8S** + Phe	0.022	-	–200.00	-	–162.50	-	–37.50	-
**MR-8S** + Ser	144.7	139.2	–42.17	47.26	–21.06	72.01	–21.11	–24.75
**MR-8S** + Val	1000	0.001	8.70	–0.29	26.85	52.11	–18.15	–52.40
**MR-8S**+α66–80	0.001	0.825	–17.23	–14.27	35.50	21.27	–52.73	–35.54

Arg = arginine; Asp = aspartic acid; His = aistidine; Ile = isoleucine; Leu = leucine; Lys = lysine; Phe = phenylalanine; Ser = serine; Val = valine.

### 3.7 Computational Studies: Mechanistic Basis for Preventing αA66–80 Crystallin Peptide Aggregation

Experimental results show that resorcinarenes **UR-4S** and **LR-4S** exhibit weak aggregation blocking activity, **UR-4A** demonstrates moderate inhibition^7^ and **MR-8S** is the most effective among the four. The structures of these resorcinarenes, along with their geometries optimized using Density Functional Theory (DFT) at the B3LYP^33^/6–31G­(d,p)^34,35^ level with the Gaussian 16 suite^56^ of quantum chemical programs, are shown in Figure 1. To gain molecular-level insight into this trend, MD simulations were performed with two objectives: (a) to explore the aggregation mechanism of the αA66–80 Crystallin peptide and identify the factors driving it, and (b) to investigate the interactions between functionalized resorcinarenes and the αA66–80 Crystallin peptide, focusing on how these interactions contribute to aggregation inhibition. The findings of our investigation are discussed in detail in the following sections.

#### 3.7.1 Investigation of the αA66–80-Crystallin Peptide Aggregation Mechanism

To elucidate the aggregation mechanism of the αA66–80 peptide, we implemented a two-stage simulation framework. Initial analysis focused on dimer formation to characterize early intermolecular recognition events, followed by four-chain simulations to examine progression toward tetrameric assemblies. Although DLS and TEM detect mature fibrillar aggregates at micrometer length scales, such late-stage species are inaccessible to conventional all-atom MD simulations. Accordingly, our computational strategy was designed to capture early self-association processes at atomic resolution. Dimers and tetramers serve as tractable models of the nucleation phase, enabling identification of the intermolecular contacts and hydrophobic interactions that stabilize nascent oligomers. The stabilization and conformational organization observed at these stages are consistent with the known aggregation propensity of the αA66–80 peptide.

The initial peptide conformation was derived from the experimentally resolved structure of human αA-Crystallin (PDB ID: 6T1R),^57^ representing the native oligomeric assembly of the protein. A single monomeric chain (chain A) was extracted and protonated at physiological pH (7.4) using the H++ server.^59–61^ The αA66–80 segment was then trimmed to generate the isolated peptide, see Figure 7. Details of the structure preparation protocol are provided in Section VIII of the Supporting Information This approach preserves the peptide’s native structural context, providing a realistic and structurally grounded starting geometry rather than an idealized or fully extended conformation. Dimeric and tetrameric systems were constructed by placing two and four peptide chains, respectively, within a 40 Å cubic simulation box using Packmol.^62^ The peptides were randomly distributed within the box, maintaining a minimum interpeptide separation of 2.0 Å. No predefined intermolecular contacts or specific oligomeric arrangements were enforced, allowing unbiased aggregation during the simulation. Thus, the starting monomer structure was informed by the native folded protein, each peptide was free to relax in aqueous solution and associate spontaneously during the simulations. This protocol ensures an unbiased exploration of early aggregation events without artificially enforcing a predetermined folded or aggregated state.

**Figure 7:**
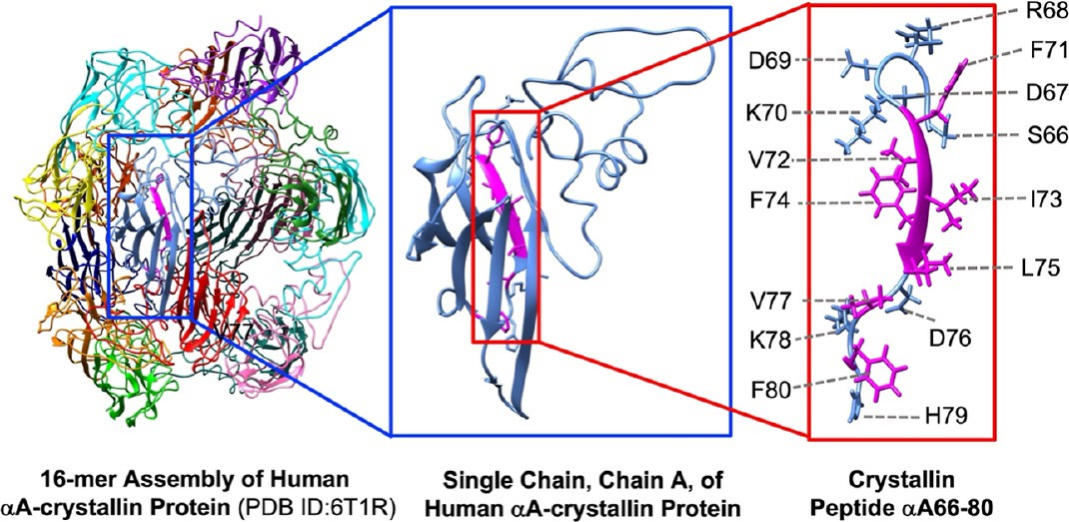
Preparation of the αA66–80 Crystallin peptide. The human αA-Crystallin sixtee*n*-mer (PDB ID: 6T1R)^57^ was obtained from the RCSB Protein Data Bank and processed using Chimera^58^ to retain chain A. Protonation states were assigned at physiological pH 7.4 with the H++ server,^59–61^ and the structure was trimmed to isolate residues S66–F80. Hydrophobic residues of the peptide are depicted in magenta, whereas hydrophilic residues are shown in blue.

The Figure 7 illustrates the residue-wise composition of the αA66–80 Crystallin peptide. The peptide comprises 15 residues, including seven hydrophobic and eight hydrophilic amino acids. Five hydrophobic residues (F71–L75) are clustered in the central region, forming a well-defined hydrophobic core, whereas the N-terminal segment (S66–K70) is predominantly hydrophilic. The C-terminal region contains a mixture of hydrophilic (D76, K78, H79) and hydrophobic (V77, F80) residues. To visually highlight this distribution, hydrophobic residues are shown in magenta and hydrophilic residues in blue, a color scheme consistently applied throughout all subsequent figures, unless stated otherwise.

Following system construction, MD simulations were performed using the AMBER24 software package,^38,39^ employing the TIP3P^63^ water model and the Amber ff19SB^64,65^ force field for the peptide. The production simulations consisted of three independent replicas of 100 ns each. The details of molecular dynamics simulations are documented in the Supporting Information (Section IX). The progression of the structural dynamics is illustrated in Figure 8. In the dimer system, hydrophobic residues progressively clustered to minimize water exposure, while hydrophilic residues remained solvent-exposed and interacted with hydrophilic residues from the other chain. By 50 ns, partial segregation of hydrophobic and hydrophilic regions was observed, and by 100 ns, hydrophobic residues from both chains had tightly clustered in the core, consistent with aggregation behavior. Similar aggregation pattern was observed for the tetramer across all three replicas, the representative replica is shown alongside the dimer results in Figure 8.

**Figure 8:**
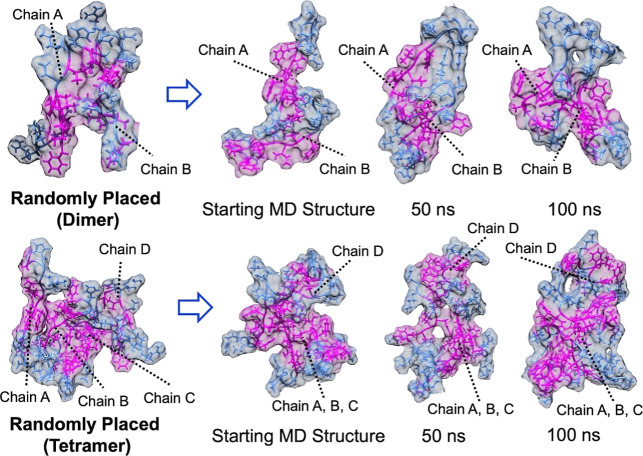
Representative snapshots from 100 ns MD simulations of the αA66–80 Crystallin peptide in dimer and tetramer states, shown at randomly placed, at the start of the simulation (after equilibration), 50 ns, and 100 ns. Hydrophobic residues are depicted in magenta and hydrophilic residues in blue. Progressive clustering of hydrophobic residues toward the core and solvent exposure of hydrophilic residues illustrates the peptide aggregation process.

Aggregation behavior was quantified through three trajectory analyses: (a) Radius of Gyration (RoG), (b) Solvent-Accessible Surface Area (SASA), and (c) Root-Mean-Square Fluctuation (RMSF), as summarized in Figure 9. Each plot represents averages over three replicas, with individual replica data provided in Supporting Information (Section X, Figures S26–S31). To further assess structural stability and equilibration, additional RMSD, RoG, and SASA analyses were performed on a per-peptide-chain basis and are presented in the Supporting Information (Figures S32–S37). It should be noted that the analyses shown in Figure 9 describe residue-wise properties of the complete dimeric and tetrameric assemblies, whereas Figures S32–S37 focus on individual peptide-chain behavior. Both the dimer and tetramer systems exhibit rapid initial structural relaxation followed by convergence of the monitored properties, indicating the formation of well-equilibrated conformational ensembles within the simulation time scale. This behavior is consistent with previous molecular dynamics studies of short peptides, where RMSD stabilization is commonly observed within the first tens of nanoseconds.^66–71^

**Figure 9:**
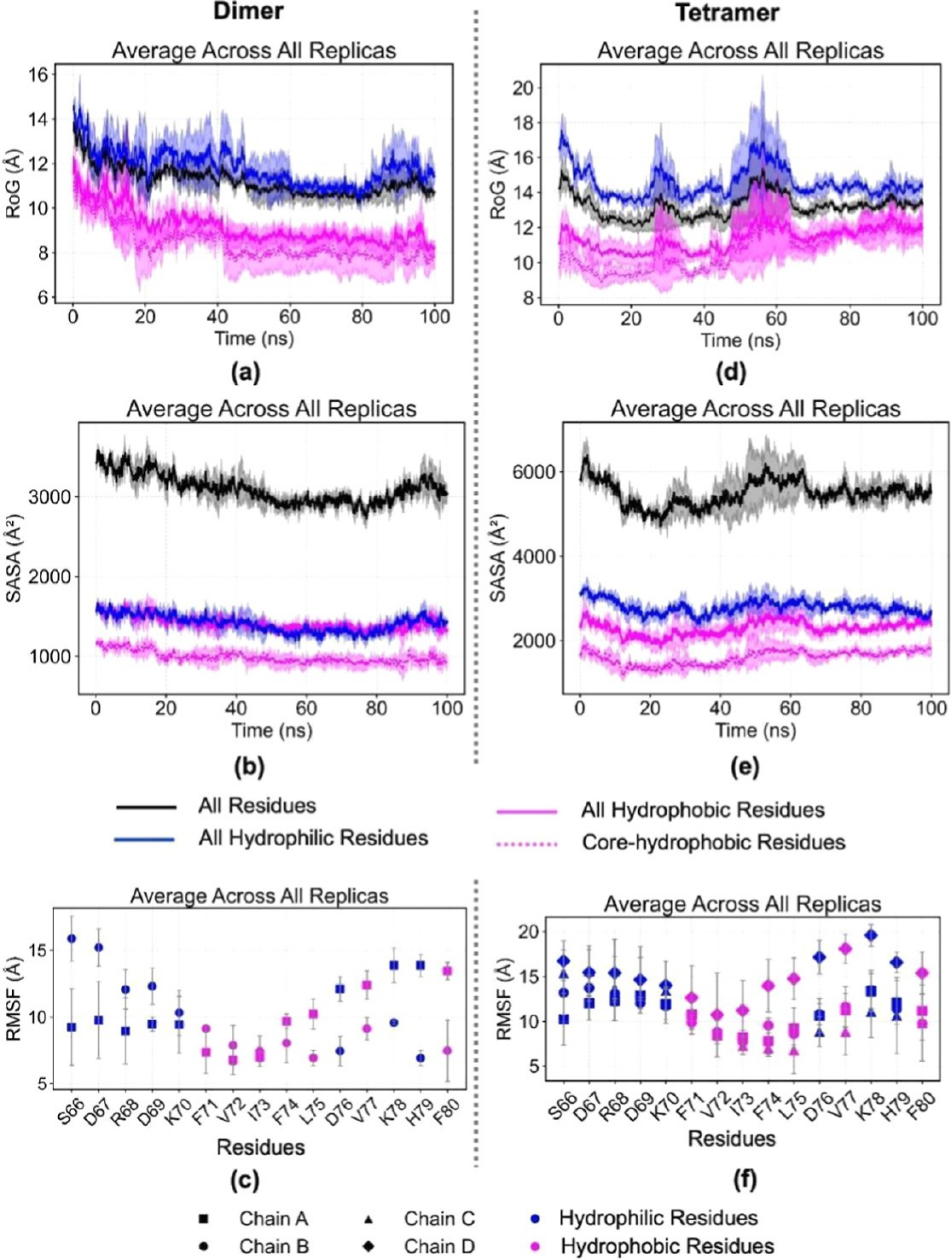
Trajectory analysis of αA66–80 peptide aggregation averaged over three replicas. (a,d) Radius of gyration (RoG) for dimer and tetramer, respectively, showing structural compaction with core hydrophobic residues exhibiting the lowest RoG. (b,e) Solvent-accessible surface area (SASA) for dimer and tetramer, indicating reduced solvent exposure upon aggregation, particularly for core hydrophobic residues. (c,f) Root-mean-square fluctuation (RMSF) for dimer and tetramer, showing minimal fluctuations in core residues, consistent with stable interchain interactions driving aggregation.

Overall, both the dimer and tetramer systems exhibit clear signs of compaction during the simulation. The RoG values for all residues decrease over time, reflecting the transition from initially dispersed chains to more compact aggregated states (Figure 9a,d). Hydrophilic residues display a comparatively higher RoG throughout, suggesting less compact packing, whereas hydrophobic residues–particularly the core hydrophobic ones–show a pronounced reduction in RoG, consistent with their key role in driving aggregation. This trend is further supported by the SASA analysis (Figure 9b,e), which reveals a steady decrease in total solvent-accessible surface area over time. The reduction is most pronounced for core hydrophobic residues, indicating reduced solvent exposure and enhanced packing within the aggregate. Finally, RMSF analysis (Figure 9c,f) highlights that core hydrophobic residues (F71-L75) undergo minimal fluctuations, demonstrating their stabilizing role in the aggregate’s interior. In contrast, the N-terminal hydrophilic residues (S66–K70) and the C-terminal residues–which include both hydrophilic (D76, K78, H79) and hydrophobic (V77, F80) residues–display enhanced flexibility, suggesting their role in peripheral structural rearrangements that accompany aggregation. Collectively, these analyses reveal consistent aggregation patterns across both dimeric and tetrameric systems, emphasizing the central role of core hydrophobic residues in promoting and stabilizing aggregation.

To further characterize the intermolecular interactions driving peptide self-association, linear interaction energy (lie) analysis was performed for both the dimer and tetramer systems (Section X.III, Figures S38–S42). Decomposition of interaction energies into electrostatic and van der Waals components revealed that electrostatic stabilization is primarily mediated by hydrophilic–hydrophilic residue interactions, whereas hydrophobic residues contribute predominantly to the van der Waals component. Notably, the interactions between the core-hydrophobic residues are found to be more pronounced in the tetramer, underscoring their critical role in aggregation-driven stabilization, See Figure S38. Together with RoG, SASA, and RMSF analyses, these findings reinforce the central role of core hydrophobic residues in promoting oligomerization and highlight them as potential targets for aggregation inhibition.

#### 3.7.2 Resorcinarene-Peptide Interactions in Aggregation Inhibition

In the previous section, we concluded that the core hydrophobic region of the αA66–80 Crystallin peptide plays a critical role in its aggregation. Experimental studies have demonstrated that functionalized resorcinarenes can interfere with this process and reduce aggregation. Among the four resorcinarenes tested, **UR-4S** and **LR-4S** showed weak inhibition, **UR-4A** displayed moderate activity, while **MR-8S** emerged as the most effective inhibitor. To rationalize these experimental observations at the molecular level, we investigated the structural dynamics of the peptide in the presence of each resorcinarene, focusing on their specific interaction patterns. As discussed in the experimental results, DLS and TEM experiments performed using a 1:1 resorcinarene-αA66–80 peptide ratio following 7 days of incubation, revealed a significant reduction in aggregate formation. DLS measurements further indicated that all tested resorcinarenes reduced aggregation to varying extents when mixed at a 1:1 ratio. Notably, even at a substoichiometric ratio (0.2:1), a measurable decrease in the average size of oligomeric assemblies was observed, whereas at a 1:1 ratio, aggregate size was reduced by approximately 9-fold. These results suggest that a 1:1 ratio is sufficient to significantly disrupt early nucleation events. Accordingly, we modeled a 1:1 resorcinarene-αA66–80 peptide complex as the minimal system for our MD simulations. Our analysis reveals key interaction patterns that help explain the experimentally observed inhibition trends. The details of these investigations are presented in the following section.

To begin, we employed the docking software Smina^72^ to dock the DFT-optimized resorcinarenes (optimized at B3LYP^33^/6–31G­(d,p)^34,35^ level of theory), namely **UR-4S**, **LR-4S**, **MR-8S**, and **UR-4A** onto the αA66–80 peptide. The docking results (presented in the Supporting Information (Section XI, Figures S43–S46) revealed distinct binding preferences. For **UR-4S**, the best-ranked pose was located around residue F80, while the remaining poses clustered near residue F74. In the case of **LR-4S** and **MR-8S**, all docking poses were consistently clustered around F74. Interestingly, **UR-4A** exhibited a slightly different pattern: its docking poses spanned the core hydrophobic residues (F71–L75) and also extended interactions to the flanking hydrophilic residues K70 and K78. Overall, these results suggest that all four resorcinarenes preferentially target the core hydrophobic region of the peptide, with **UR-4A** showing an extended affinity for both hydrophobic and hydrophilic residues. The best poses were selected for subsequent structural dynamics simulations; for **UR-4S**, the second-best pose was chosen to ensure consistency in comparative analysis. The evolution of these docked poses during structural dynamics is discussed in the following section and depicted in Figure 10. All MD simulations were carried out in explicit solvent with NaCl added to achieve physiological ionic strength 0.15 M, ensuring charge neutrality and mimicking in vivo conditions. Under these conditions, all resorcinarenes, including **MR-8S**, remained structurally stable and exhibited consistent conformational behavior and interaction patterns. MD simulations were performed using the same protocol as implemented in the investigation of aggregation in the dimeric and tetrameric oligomeric assemblies of the αA66–80 peptide. The resorcinarene parameters were generated using Antechamber with the AM1-BCC charge scheme and the GAFF2 ^73^ force field. The final MD production runs were performed as three independent 100 ns replicas for each resorcinarene-αA66–80 peptide complex. To enable a comparable assessment of core shielding using SASA, the αA66–80 peptide monomer was simulated using the same MD protocol in the replicates of three. Details of the MD simulations are provided in Section IX of the Supporting Information

**Figure 10:**
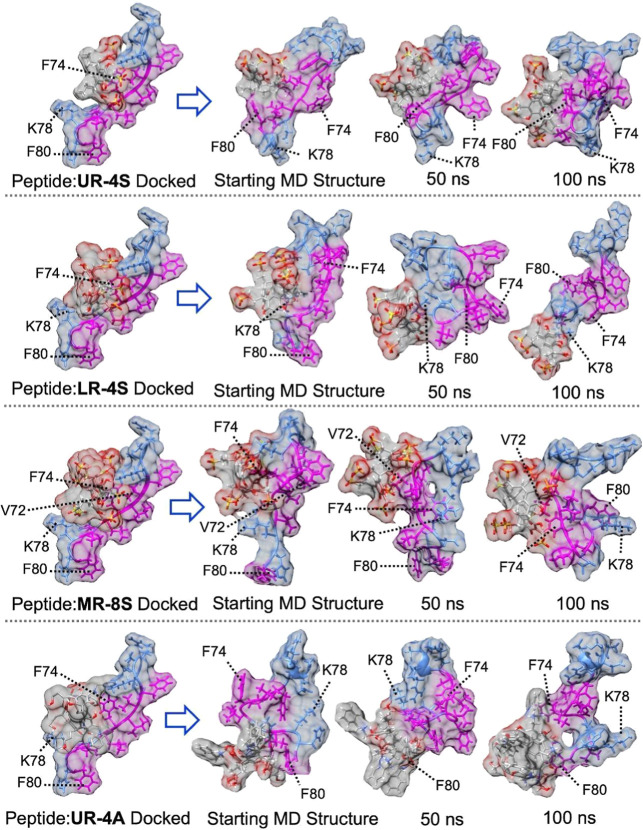
Representative snapshots from 100 ns MD simulations showing the interactions between the αA66–80 Crystallin peptide and the four tested resorcinarenes at 0 ns (start), 50 ns, and 100 ns. Hydrophobic residues are depicted in magenta, hydrophilic residues in blue, and resorcinarenes are colored by atom type (carbon: gray, oxygen: red, sulfur: yellow, nitrogen: blue). Among the resorcinarenes, **MR-8S** consistently shields the core hydrophobic residues (F71–L75), demonstrating its effectiveness as the strongest inhibitor of aggregation.

Figure 10 illustrates the structural dynamics of resorcinarene-αA66–80 peptide complex interactions over a 100 ns MD simulation. In all cases, the starting structure was obtained from the docking pose, with the resorcinarene positioned around the central hydrophobic core (F71–L75). At the onset of the MD simulation, all resorcinarenes engaged with the core hydrophobic residues. However, their binding behavior diverged as the simulation progressed. It should be noted that the observed differences between the docking poses and the starting MD structures arise from the explicit water relaxation procedure applied during system preparation, followed by a 20 ns equilibration phase. Thus, the starting MD structures represent the equilibrated conformations at the beginning of the production run. Resorcinarene **UR-4S** rapidly lost contact with the central hydrophobic core and instead interacted primarily with F80. This indicates its preference for hydrophobic interactions, but at the expense of leaving the core (F71–L75) exposed, thereby permitting aggregation. In contrast, **LR-4S** displayed strong affinity for the hydrophilic residue K78 throughout the simulation, effectively unblocking the core region. **MR-8S** exhibited the most stable interaction profile, maintaining persistent contact with the hydrophobic core residues for the entire trajectory. In particular, F74 or V72 remained in closest proximity to **MR-8S**, suggesting that **MR-8S** strongly masks the hydrophobic core, thereby reducing its exposure and functioning as the most effective inhibitor of aggregation. **UR-4A** showed a dual binding pattern, interacting with both hydrophobic (V72) and hydrophilic (D76) residues. As a result, it only partially shielded the hydrophobic core, leaving part of the region accessible for aggregation, thus acting as an intermediate inhibitor.

These observations are further supported by RMSF analysis of the MD trajectories, see Figure 11, where the αA66–80 peptide monomer profile is included as a reference to allow direct comparison between the complexed and uncomplexed states. Each plot represents averages over three replicas, with individual replica data provided in Supporting Information (Section XII, Figure S47–S51). In its uncomplexed form the peptide exhibits relatively low flexibility within the hydrophobic core region (F71–L75). This reduced mobility likely arises from self-association among these five hydrophobic residues, which tend to cluster together in order to minimize solvent exposure. Their intrinsic aversion to water promotes intrapeptide hydrophobic contacts, thereby restricting local fluctuations. Upon complexation with the resorcinarenes, distinct changes in the RMSF profiles of the core residues are observed, reflecting differences in binding modes and core engagement. In the case of **UR-4S**, the hydrophobic core residues display RMSF values comparable to the αA66–80 peptide monomer, indicating minimal interaction with the core region. Instead, **UR-4S** significantly reduces the fluctuations of residue F80, consistent with MD trajectories that show preferential binding at the peptide tail, leaving the aggregation-prone core largely unmasked. For **LR-4S**, reduced RMSF values are observed primarily for hydrophilic residues D76, K78, and H79, indicating preferential interaction with polar regions of the peptide. This binding mode disrupts stabilizing intramolecular contacts within the hydrophobic core, leading to increased flexibility of core residues and maintaining their availability for intermolecular association. This behavior is consistent with its comparatively weak aggregation–inhibitory activity. **UR-4A** exhibits an intermediate effect, influencing both hydrophobic and hydrophilic residues and interacting transiently with the core, resulting in moderate inhibition efficiency. In contrast, **MR-8S** produces the most pronounced reduction in core-residue flexibility. Across all resorcinarenes, **MR-8S** exhibits the largest reduction in RMSF values across the key core residues V72–L75, demonstrating the strongest dynamic confinement of the aggregation-prone region. This pronounced stabilization highlights its superior core-masking efficiency. Overall, RMSF analysis serves as a reliable quantitative descriptor for differentiating the relative core-masking efficiency and inhibitory potential of the investigated resorcinarenes.

**Figure 11:**
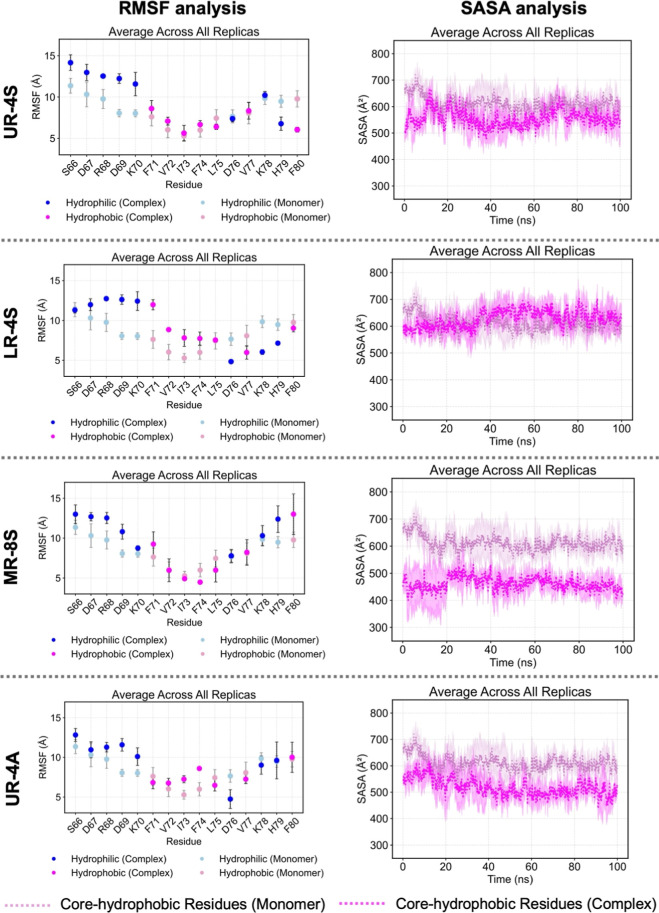
Comparative RMSF and SASA analysis of the αA66–80 peptide in complex with **UR-4S**, **LR-4S**, **MR-8S**, and **UR-4A**. RMSF profiles (with the αA66–80 peptide monomer shown as a faint reference) reveal resorcinarene-dependent modulation of peptide flexibility and masking of the hydrophobic core (F71–L75). SASA profiles (with the αA66–80 peptide monomer shown as a faint reference) demonstrate reduced solvent exposure of core residues upon binding; notably, **MR-8S** exhibits the greatest reduction compared to the other systems, serving as a quantitative measure of its superior blocking activity.

To further quantify hydrophobic core shielding, we computed the SASA of residues F71–L75 for the αA66–80 peptide monomer and for each resorcinarene-αA66–80 peptide complex, see Figure 11. The SASA analysis for all peptide residues is provided in the Supporting Information (Section XII, Figures S52–S56). For clarity, only the core-residue SASA is presented in Figure 11. The αA66–80 peptide monomer exhibits an average core SASA of 611.73 ± 18.10 Å^2^ (Supporting Information Section XII, Table S3), reflecting substantial solvent exposure of the aggregation-prone region. In the presence of **LR-4S**, the core SASA (627.68 ± 25.96 Å^2^) remains comparable to, and slightly higher than, that of the αA66–80 peptide monomer, indicating negligible shielding and ineffective coverage of the hydrophobic segment. This is further supported by the near-complete overlap of the SASA time profiles for the αA66–80 peptide monomer and the **LR-4S**-αA66–80 peptide complex throughout the 100 ns simulation (Figure 11). **UR-4S** produces only a modest reduction in core SASA (554.56 ± 14.99 Å^2^), consistent with partial and localized surface coverage, as reflected by the marginal separation between the corresponding SASA curves. **UR-4A** further decreases solvent exposure (517.83 ± 28.40 Å^2^), suggesting broader engagement of the hydrophobic region, with a clearly distinguishable gap between the αA66–80 peptide monomer and complexed SASA profiles. In contrast, **MR-8S** yields the most pronounced reduction in core SASA (461.02 ± 24.38 Å^2^), representing the largest decrease relative to the αA66–80 peptide monomer. The substantial loss of solvent exposure, evident from the distinct separation between the SASA time series, provides direct quantitative evidence of effective hydrophobic core shielding. Overall, the SASA analysis clearly differentiates the resorcinarene derivatives, ranking their shielding efficiency as **MR-8S** > **UR-4A** > **UR-4S** > **LR-4S**, in full agreement with the RMSF trends and their experimentally observed inhibitory activities.

The interactions between the resorcinarenes and the peptide were further investigated using lie analysis, which revealed that inhibitor potency correlates strongly with effective engagement of the hydrophobic core (F71–L75). Detailed results of this analysis are provided in the Supporting Information (Section XII, Figure S57–S61). **MR-8S** exhibits dominant van der Waals stabilization arising from the core hydrophobic residues, indicating efficient core masking and the highest complex stability among the tested derivatives. **UR-4A** shows intermediate stabilization with partial involvement of the core region. For **UR-4S**, van der Waals stabilization arises from hydrophobic residues; however, contributions from the aggregation-prone core are overshadowed by peripheral residues, indicating incomplete core masking and moderate inhibitory potency. In contrast, **LR-4S** is primarily stabilized through hydrophilic interactions, with minimal contribution from the core hydrophobic residues, resulting in weaker inhibitory efficacy. Overall, effective aggregation inhibition is directly associated with strong van der Waals interactions originating from the aggregation-prone hydrophobic core.

Thus, the RMSF and SASA analysis provide a direct and mechanistically meaningful assessment of hydrophobic core shielding. These quantitative metrics demonstrate that **MR-8S** uniquely maintains long-lived interactions with multiple hydrophobic core residues and produces the greatest reduction in core solvent exposure, consistent with the enhanced stabilization observed in the lie analysis. Collectively, these results identify hydrophobic core masking as the key determinant of resorcinarenes efficacy and rationalize the superior ability of **MR-8S** to suppress early stage αA66–80 peptide aggregation.

Another important aspect is that the simulations were performed in the presence of 0.15 M NaCl to approximate physiological ionic strength. Given the high charge density of the octasulfonated macrocycle **MR-8S** (−8), it is essential to assess whether electrostatic screening and/or specific ion association modulate its binding affinity and preferred orientation relative to the peptide. In addition to **MR-8S**, the other resorcinarenes examined in this study differ significantly in their net charge: **UR-4S** and **LR-4S** are tetrasulfonated (−4), whereas **UR-4A** is tetraaminated (+4). All systems were neutralized with appropriate counterions, resulting in different ionic environments that may contribute to their distinct inhibitory activities. To evaluate the role of ionic screening and ion–macrocycle interactions, we performed detailed contact analysis of ions with the peptide–resorcinarene complexes over the course of the simulations. The details of the contact analysis are provided in Supporting Information Section IX, and the results are presented in Figure S62. The analysis indicates that ion association is governed by the net charge of the macrocycles and varies systematically across the different resorcinarene systems. On average, approximately two Na^+^ ions remain in close proximity to the tetraanionic resorcinarenes (**UR-4S** and **LR-4S**), while two Cl^–^ ions associate with the cationic **UR-4A**. In contrast, the octaanionic **MR-8S** is coordinated by approximately four Na^+^ ions, consistent with its higher charge density. Despite these differences, ion–macrocycle interactions are highly dynamic and do not impose a fixed orientation on the macrocycles relative to the peptide. For example, in the **MR-8S** system, Na^+^ ions continuously exchange between interactions with the upper and lower rims of the macrocycle throughout the simulation. Representative snapshots (Figure S63, Supporting Information) taken at 0, 20, 40, 60, 80, and 100 ns illustrate this dynamic behavior, demonstrating that ion association does not constrain the binding orientation of **MR-8S**. These findings suggest that while electrostatic screening and ion association are present and system-dependent, they do not dominate the binding mode; instead, intrinsic macrocycle–peptide interactions govern the observed inhibitory activity.

#### 3.7.3 MR-8S-Induced Disruption of αA66–80 Peptide Aggregates

To further investigate the inhibitory mechanism of **MR-8S** beyond monomer-level peptide recognition, additional MD simulations were performed on preassembled dimeric and tetrameric αA66–80 peptide aggregates in the presence of **MR-8S**. These oligomeric models provide a framework for examining higher-order 2:1 and 4:1 peptide:**MR-8S** assemblies and for evaluating whether **MR-8S** can destabilize pre-existing aggregation-prone structures and reverse peptide self-association. Aggregation analysis of the apo dimeric and tetrameric systems, as discussed in the peptide aggregation mechanism, demonstrated that aggregation of the αA66–80 peptide is primarily stabilized by intermolecular hydrophobic interactions involving the central core residues F71–L75. These hydrophobic-core interactions promote persistent interpeptide contacts and facilitate the formation of early oligomeric assemblies. Based on these observations, representative dimeric and tetrameric peptide conformations were selected to construct the corresponding 2:1 and 4:1 peptide:**MR-8S** systems, enabling direct evaluation of how **MR-8S** perturbs aggregation-driving peptide–peptide interactions (see Supporting Information Section XIII for details of system preparation and Figure S64 for docking results).

The resulting systems were subjected to 300 ns MD simulations to compare interpeptide interaction patterns and structural evolution in the absence and presence of **MR-8S**. To enable direct comparison between intrinsic aggregate stability and **MR-8S**-induced dissociation behavior, the previously simulated apo dimeric and tetrameric assemblies were also extended to 300 ns simulations. Structural analysis, including RMSD, radius of gyration (RoG), and SASA profiles for the apo dimeric and tetrameric systems, are presented in Figures S65–S70 of the Supporting Information. This analysis showed that the overall trends in structural properties remained consistent throughout the extended simulations, with no substantial deviations observed beyond the initial equilibration period. The results indicate that both dimeric and tetrameric assemblies remained structurally stable over the extended time scale and that the essential aggregation characteristics were already established within the initial 100 ns simulations. The effects of **MR-8S** on peptide aggregation and aggregate dissociation are discussed below.

Figure 12 presents the time-resolved structural evolution of the αA66–80 peptide assemblies in the presence of **MR-8S** during the 300 ns molecular dynamics simulations, demonstrating the progressive destabilization and dissociation of both the dimeric (2:1 peptide:**MR-8S**) and tetrameric (4:1 peptide:**MR-8S**) aggregates. In the 2:1 peptide:**MR-8S** system, strong intermolecular contacts involving the hydrophobic core region F71–L75 (highlighted in magenta) are clearly observed at the beginning of the simulation. As the trajectory progresses, **MR-8S** increasingly perturbs these intermolecular interactions, leading to gradual weakening of the peptide–peptide contacts and progressive separation of the peptide chains. Across all three replicas, a consistent disruption of interactions between chains A and B was observed, with chain B emerging as the most dynamic component and progressively detaching from the complex. However, due to periodic boundary conditions in the MD simulation setup, the dissociated peptide can re-enter the simulation box and transiently re-establish contacts, which can make strictly uniform time-interval snapshots (e.g., every 100 ns) visually misleading. To more accurately capture the progressive loss of intermolecular core contacts, representative frames were therefore selected based on structural state rather than fixed time spacing, shown at 98, 219, and 284 ns in Figure 12. Despite these periodic artifacts in spatial positioning, the simulations consistently demonstrate a clear and sustained disruption of hydrophobic core interactions throughout the trajectory. A similar destabilization mechanism is observed for the 4:1 peptide:**MR-8S** tetrameric assembly. At the start of the simulation, extensive intermolecular interactions involving the F71–L75 hydrophobic core residues are present among all four peptide chains. As the simulation progresses, chains A, C, and D remain associated and form a partially retained cluster, whereas chain B gradually becomes increasingly dynamic and separates from the assembly. Similar to the dimeric system, periodic boundary effects occasionally allow the dissociated peptide chain to reapproach the cluster and transiently establish new interactions. Nevertheless, the representative frames at 120, 207, and 285 ns clearly demonstrate the progressive disruption of hydrophobic-core contacts between chain B and the remaining A-C–D cluster, consistent with destabilization and partial dissociation of the tetrameric aggregate. These observations are further quantified by the RMSF and hydrophobic-core contact analysis described in the following section.

**Figure 12:**
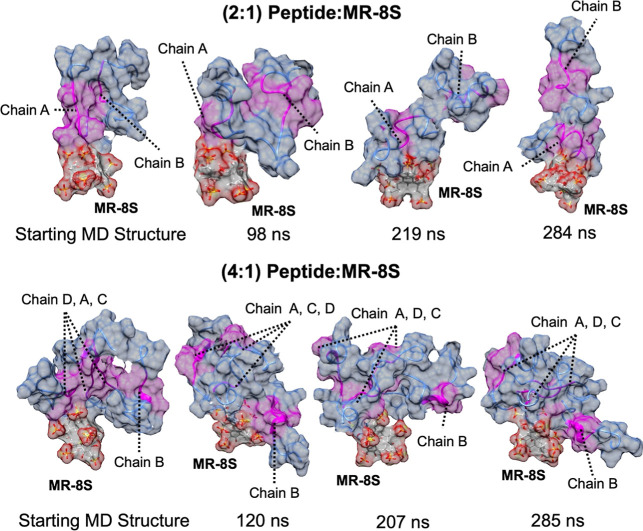
Time-resolved structural evolution of αA66–80 peptide assemblies in the presence of **MR-8S** during the 300 ns MD simulations. Representative snapshots are shown from the beginning of the simulation and at subsequent stages selected to illustrate the progressive dissociation of peptide assemblies, with snapshots separated by approximately 100 ns for both the dimer (2:1 peptide:**MR-8S**) and tetramer (4:1 peptide:**MR-8S**) systems. The hydrophobic core region (F71–L75) is highlighted in magenta in both systems.

The **MR-8S**–induced perturbation of interpeptide interactions in both dimeric and tetrameric αA66–80 assemblies was quantitatively analyzed using residue-wise RMSF profiles and time-resolved hydrophobic core contact analysis. For comparison, these metrics were also evaluated for the corresponding apo dimer and tetramer systems simulated over 300 ns. In the case of the dimeric assembly, the RMSF comparison presented in Figure 13 shows that the apo system exhibits overall lower residue fluctuations, particularly within the aggregation-prone hydrophobic core region F71–L75. This reduced flexibility is consistent with stable interpeptide packing and persistent hydrophobic interactions between chains A and B, which maintain the compactness of the dimeric assembly. In contrast, upon **MR-8S** binding, these core residues exhibit a marked increase in fluctuations, indicating a loss of compactness and disruption of hydrophobic core integrity between the two peptide chains. Notably, chain B displays significantly higher flexibility compared to chain A, consistent with its progressive dissociation from the assembly, while chain A remains more persistently associated with **MR-8S**. This behavior is in strong agreement with the structural evolution observed in Figure 12. A similar but more complex trend is observed for the tetrameric system. In the apo tetramer, chains A, B, and C form a relatively stable cluster, while chain D exhibits slightly higher fluctuations but remains associated with the oligomeric core. This is reflected in only a modest increase in RMSF for chain D, indicating partial peripheral flexibility rather than complete dissociation. In contrast, in the presence of **MR-8S**, the system reorganizes such that chains A, C, and D form cluster, whereas chain B becomes highly dynamic and undergoes progressive dissociation. This is clearly captured by the elevated RMSF values of chain B, which distinguish it from the remaining clustered chains.

**Figure 13:**
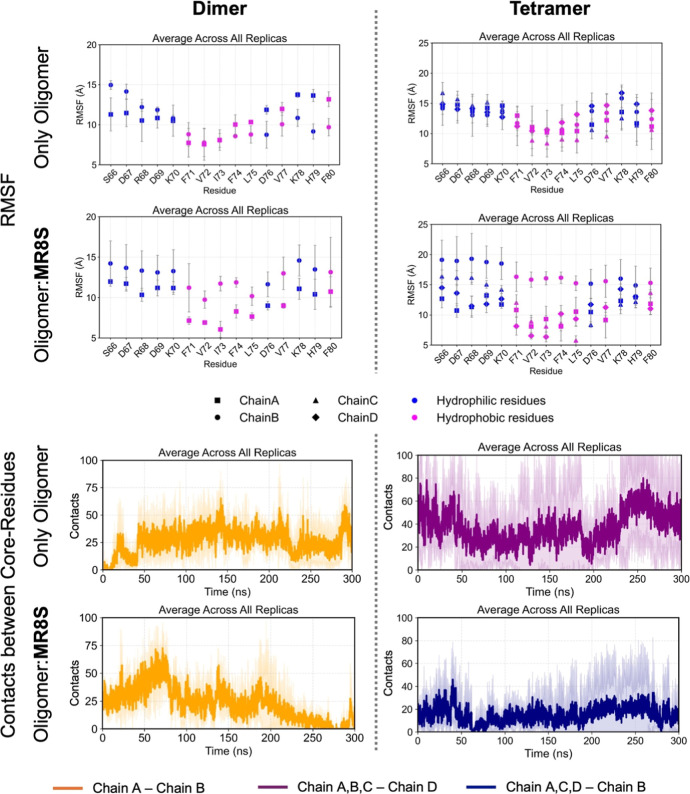
Quantitative analysis of interpeptide interactions in apo and **MR-8S**-bound dimeric and tetrameric αA66–80 peptide assemblies during 300 ns MD simulations. RMSF profiles show increased flexibility of the aggregation-prone hydrophobic core residues (F71–L75) upon **MR-8S** binding, consistent with destabilization and partial dissociation of peptide chains. Time-resolved hydrophobic core contact analysis reveals stable interchain contacts in the apo assemblies, whereas **MR-8S** induces progressive loss of hydrophobic interactions in both dimeric and tetrameric systems. For the dimer, contacts were monitored between chains A and B. In the apo tetramer, contacts were analyzed between the A,B,C cluster and chain D, while in the **MR-8S**-bound tetramer, contacts were monitored between the A,C,D cluster and chain B. Together, these results demonstrate that **MR-8S** disrupts hydrophobic packing essential for oligomer stability and promotes disassembly of early stage peptide aggregates.

These observations are further supported by the interpeptide hydrophobic contact analysis. In the apo dimer, contacts between core residues of chains A and B remain highly persistent, fluctuating around an average value of approximately 25, indicating stable hydrophobic packing. Upon **MR-8S** binding, these contacts become increasingly unstable: they initially show transient enhancement but subsequently decrease significantly over time, ultimately leading to near-complete loss of interchain core contacts, consistent with dissociation of chain B from chain A. This clearly demonstrates that **MR-8S** disrupts early stage aggregation-prone interactions critical for dimer stability. In the tetrameric system, a similar comparison of core contacts was performed between chain A, B, C and chain D in the apo state, and between the A, C, D cluster and chain B in the **MR-8S**-bound state. In the apo assembly, interchain core contacts remain relatively stable across the clustered chains, while chain D remains weakly associated but does not fully dissociate. However, in the presence of **MR-8S**, a pronounced reduction in core contacts is observed between chain B and the A,C,D cluster, indicating selective destabilization and dissociation of chain B. Overall, the comparison between apo and **MR-8S**-bound systems clearly demonstrates a significant reduction in hydrophobic core contacts within both oligomeric assemblies, highlighting **MR-8S**-induced destabilization and partial disassembly of preformed peptide aggregates. These results collectively suggest that **MR-8S** effectively targets and disrupts hydrophobic interactions that are essential for early stage oligomer stability, thereby interfering with peptide self-association and aggregation progression. Replia-wise analysis of the properties depicted in Figure 13 are provided in Supporting Information Figures S71–S78.

The choice of contact analysis in the tetrameric system was guided by the dominant structural behavior observed during the simulations and supported by the RMSF profiles. In the apo tetramer, chains A, B, and C remained associated as a relatively stable cluster, while chain D exhibited comparatively higher flexibility but largely retained its association with the assembly. In contrast, in the **MR-8S**-bound tetramer, chains A, C, and D formed the dominant clustered assembly, whereas chain B became highly dynamic and progressively dissociated. Therefore, the contact analysis was focused on the interactions between the A,B,C cluster and chain D in the apo system, and between the A,C,D cluster and chain B in the MR-8S-bound system, as these interfaces most clearly captured the major dissociation events induced by **MR-8S**. Although interactions within the remaining A,C,D cluster were also perturbed in the presence of **MR-8S**, these changes were highly dynamic and heterogeneous, preventing meaningful quantitative comparison. Consequently, these intracluster perturbations were not included in the discussion.

Thus, our findings demonstrate that **MR-8S** disrupts the key interpeptide hydrophobic interactions within the F71–L75 core region, which primarily drive peptide association and oligomer stabilization. This disruption results in a progressive loss of interpeptide contacts, increased flexibility and perturbation of core residues, and structural reorganization of the assemblies, as supported by the RMSF and contact analyses. Importantly, these observations provide a conceptual bridge between early stage nucleation and larger aggregate formation: by interfering with the hydrophobic interactions that initiate and stabilize oligomer assembly, **MR-8S** disrupts the structural alignment necessary for β-sheet propagation, thereby suppressing the formation and growth of higher-order aggregates observed experimentally. Although complete oligomer dissociation is not fully captured within the 300 ns simulation time scale, the trajectories clearly reveal early stage destabilization events that mechanistically underpin the inhibitory activity of **MR-8S**.

In summary, the computational studies reveal that αA66–80 peptide aggregation is primarily driven by strong hydrophobic core interactions involving residues F71–L75, which stabilize both dimeric and tetrameric assemblies through persistent interpeptide packing. Screening of resorcinarenes shows a clear activity trend, where **MR-8S** exhibits the strongest inhibition by effectively masking the hydrophobic core, **UR-4A** shows moderate core engagement, and **UR-4S/LR-4S** display weak or peripheral interactions. This behavior is consistently reflected in RMSF, SASA, and interaction energy analyses, identifying core shielding as the key determinant of inhibition. Importantly, **MR-8S** not only prevents aggregation at the binding stage but also disrupts preformed oligomers by weakening hydrophobic contacts and increasing chain flexibility. Overall, **MR-8S** emerges as the most effective inhibitor by targeting and destabilizing the hydrophobic core that governs peptide self-assembly.

## 4 Conclusions

We have successfully synthesized a polysulfonated resorcinarene **MR-8S** with a total of eight sulfonate groups, four located on the lower rim and four located on the upper rim. ^1^H NMR and ITC binding studies showed **MR-8S** interacts very strongly with all the amino acids that constitute the α66–80-Crystallin peptide as well as the peptide. A fluorescence aggregation assay revealed the deaggregation ability of **MR-8S** toward the α66–80-Crystallin peptide, which increased with the concentration of **MR-8S**. IC_50_, which refers to the concentration of **MR-8S** required to cause 50% deaggregation, has a calculated value of 44 ± 1 μM, which is substantially smaller than other reported resorcinarene analogues **UR-4S** and **UR-4A** but 2-fold greater than **LR-4S**. Similarly, in the presence of one equivalent of **MR-8S**, DLS experiments showed average particle size of αA66–80-Crystallin peptide aggregate was reduced almost 9-fold, from 13293 ± 1072 d. nm to 1483 ± 15 d.nm. This was further confirmed with TEM images that showed fewer fibrillar aggregates in mixtures of **MR-8S** and α66–80-Crystallin peptide compared to the pure α66–80-Crystallin peptide. Computational studies successfully rationalized the observed experimental findings. MD simulations revealed that the core hydrophobic residues of αA66–80 (F71–L75) play a central role in aggregation, as evident in both dimer and tetramer formations. In these cases, the hydrophobic core residues cluster together to minimize exposure to water, emphasizing their intrinsic hydrophobic nature. This behavior was further supported by analysis of the RoG, SASA, and RMSF. MD simulations of the peptide-resorcinarene complexes further elucidated their aggregation inhibition profiles. Among the tested resorcinarenes, **MR-8S** exhibited the strongest interaction with the hydrophobic core residues, effectively masking them and preventing aggregation. The comparative RMSF and SASA analysis highlight that effective inhibition correlates with the masking of the peptide core-hydrophobic residues by resorcinarenes, positioning **MR-8S** as the most potent inhibitor in this series. Overall, the computational study provided molecular–level understanding of αA66–80 peptide aggregation and the mechanism of resorcinarene-mediated inhibition, offering valuable guidance for the rational design of more effective aggregation inhibitors. Based on these results, **MR-8S** macrocycle, with its strong deaggregation effects, could form the basis of a molecular scaffold for the development of anticataract therapeutics. We will continue to explore the potential for such macrocycles to affect the deaggregation potential of not only the αA66–80 Crystallin peptide but also potentially the single-chain A of the αA Crystallin protein and possibly the 16-mer αA Crystallin protein.

## Supplementary Material



## Notes

The ACS Publication Web site. Synthesis, DLS, Fluorescence, TEM, NMR, ITC, and computation data are included in the Supporting Information (PDF)
