# Supplementary material for: Reversing the Fold: Polyanionic Macrocycle Dissolves αA66–80 Crystallin Peptide Aggregates
Source: Biomacromolecules. 2026 Jun 13;27(7):4523–41. doi: 10.1021/acs.biomac.6c00441 (PMC13327089; doi:10.1021/acs.biomac.6c00441)
Supplement: Supplementary file 1 [file bm6c00441_si_001.pdf]

Reversing the Fold: Polyanionic Macrocycle Dissolves αA66–80 Crystallin Peptide Aggregates

Frank Boateng Osei,[†] Yuvraj Dangat,[‡] Roi Yasay,[†] Josephine Esposto,[§] Kwaku Twum,[ǁ] Robert J. Huber,[§] Sanela Martic,[§,#] Hedieh Torabifard,[‡] and Ngong Kodiah Beyeh[†*]*

[†]Department of Chemistry, Oakland University, 146 Library Drive, Rochester, MI 48309-4479, USA

[‡]Department of Chemistry and Biochemistry, The University of Texas at Dallas, 800 West Campbell Road, Richardson, Texas 75080-3021, USA

[§]Departments of Biology, Environmental and Life Sciences Program, Trent University, ON, Canada, K9L0G2

[ǁ]Department of Pathology and Laboratory Medicine, Boston University School of Medicine, Boston, MA, USA

[#]Departments of Forensic Science, Trent University, ON, Canada, K9L0G2

Supporting Information

Contents

I. General Information 2

II. Synthesis 2

III. Dynamic Light Scattering (DLS) Experiments 9

IV. Proteostat Fluorescence Aggregation Assay 11

V. Transmission Electron Microscopy (TEM) Imaging 11

VI. ^{1}H NMR Spectroscopy 14

VII. Isothermal Titration Calorimetry (ITC) 16

VIII. Structure Preparations 22

IX. Details of Molecular Dynamics (MD) Simulations 24

X. Investigation of the αA66–80-Crystallin Peptide Aggregation Mechanism 25

XI. Molecular Docking of αA66–80 Peptide–Resorcinarene Complexes 41

XII. Investigation of Resorcinarene-Mediated Inhibition of αA66–80 Peptide Aggregation 45

XIII. Reversing the Fold by MR-8S 64

XIV. References 77

I. General Information

Receptor **MR-8S** was synthesized according to reported procedures[1]. The αA66-80 crystallin peptide was purchased from GenScript. All the solvents used were purchased from Thermo Fisher Scientific and Sigma Aldrich, USA. Fluorescence Proteostat aggregation and dynamic light scattering experiments were carried out in 10mM TRIS buffer using TAKE 3 BioTek microplate reader and Malvern Zetasizer, respectively. ^{1}H NMR was carried out on 400MHz Bruker Spectrometer. ITC measurements were carried out using NanoITC instrument from TA Instruments.

II. Synthesis

A two-phase mixture of 2-(2-bromoethyl)-1,3-dioxane, 1 (4.0 g, 20 mmol) and an aqueous solution (20 mL) of Na_2SO_3, 2 (5.0 g, 40 mmol) was stirred at 100 °C for 24 hours. To the resulting homogeneous solution was added water (20 mL), and the mixture was washed with ether (40 mL x2) to get rid of unreacted 1. To this were successively added ethanol (40 mL), resorcinol, 3 (4.0 g, 36 mmol), and concentrated HCI (6 mL). The mixture was stirred under nitrogen at 100 °C for 24 h and solvent evaporated. The residue was taken in water (60 mL) and dialyzed three times against water (2 L) using a dialysis membrane having a transport critical molecular weight of 1000 (Spectra/Por membrane MWCO 1000) to remove inorganic salts. Most of the water was removed in vacuo, and the residue was triturated from methanol to give compound 4. Compound 4 (0.01 mol), a solution of 37% formaldehyde (0.01mol) and sodium sulfite (0.01M) in H_2O (30 ml) was stirred and heated at 90–95°C for 4 h. Dilute hydrochloric acid was added after cooling until pH 7, then methanol (50 ml or more) was added to precipitate the product **MR-8S**. The solid was filtered and dried.[1]

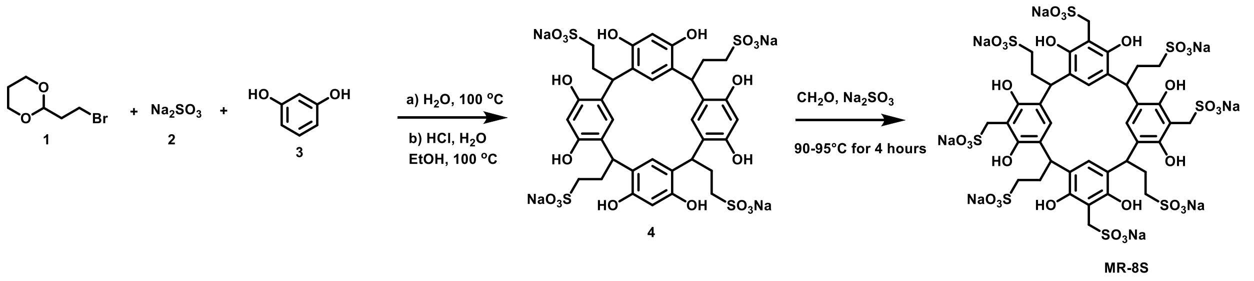

Figure S1: Schematic representation of the synthesis of resorcinarene **MR-8S**.

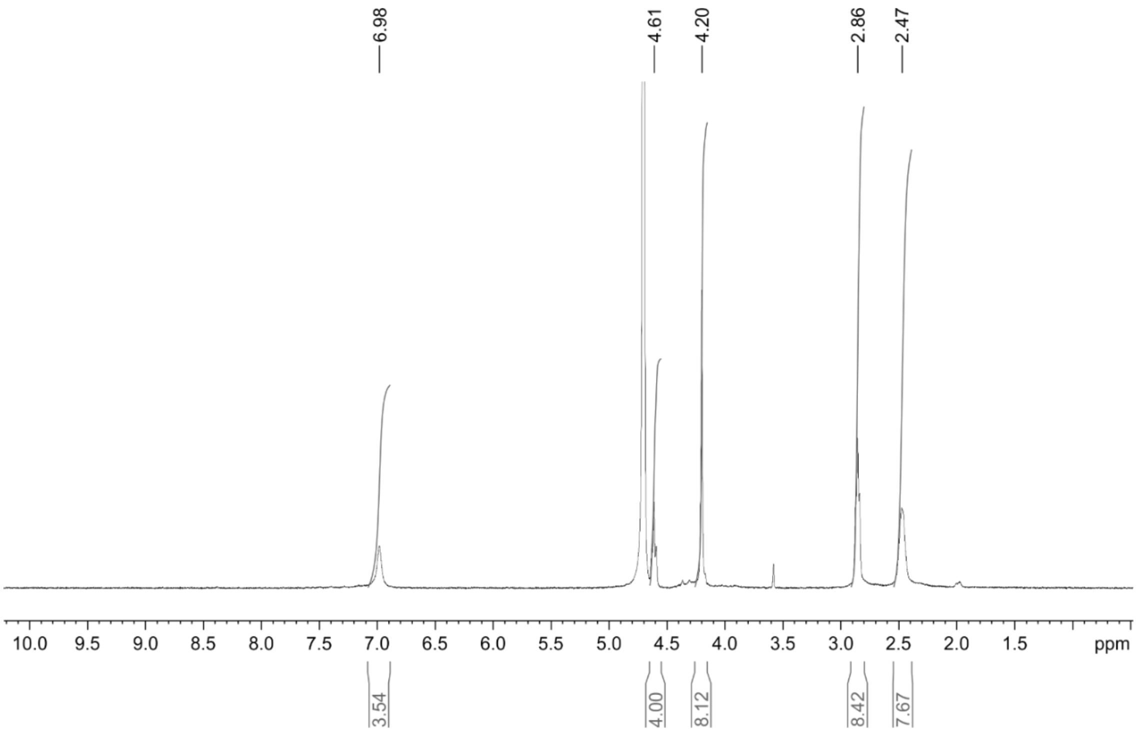

Figure S2: ^{1}H NMR spectra of octa sulfonated resorcinarene **MR-8S** at 400 MHz in D_2O at 298 K.

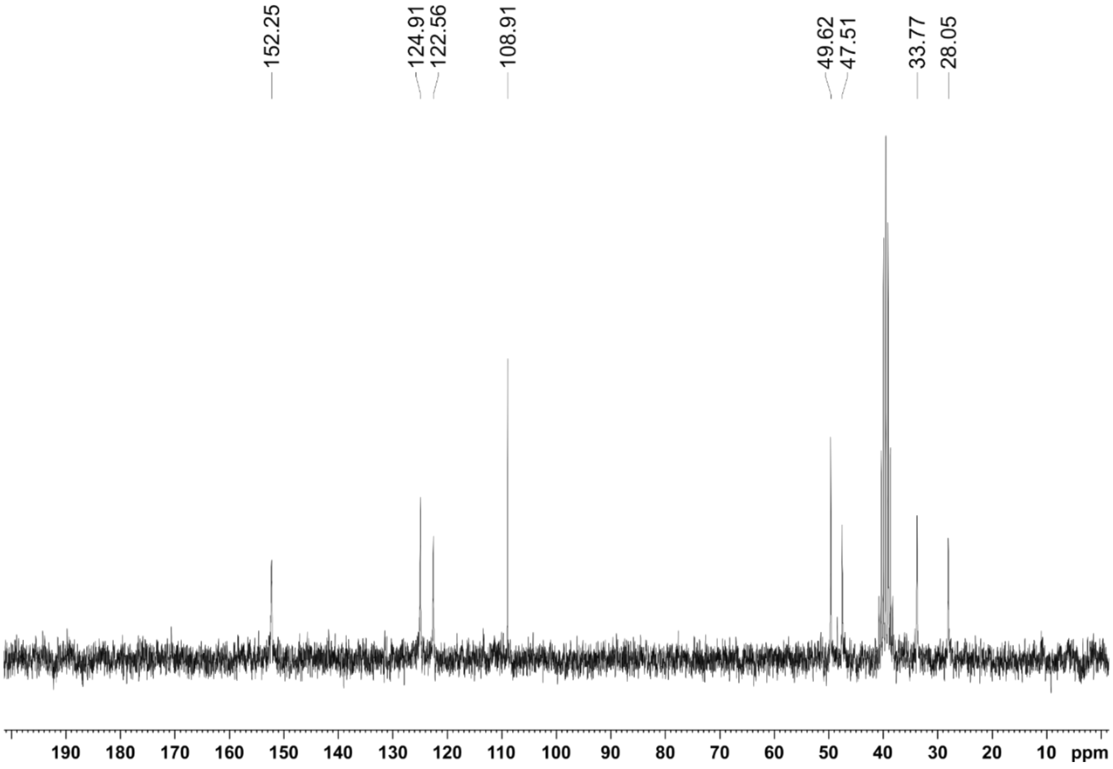

Figure S3: ^{13}C NMR spectra of octa sulfonated resorcinarene **MR-8S** at 400 MHz in D_2O at 298 K.

Mass Spectrometry: The mass spectrometric studies were performed with a Thermo Scientific Q-Exactive Plus Hybrid Quadrupole-Orbitrap mass spectrometer equipped with a heated electrospray ionization II (HESI-II) probe. The instrument was run in both positive and negative ion modes for all experiments and were performed under low temperature conditions to stabilize complexes formed (40°C). The parameters of the ion source and fragmentation (MS/MS) were optimized for the maximum abundance of the ions in this study.

Solution preparation

A 10 mM stock solution of the α66-80 crystallin peptide in water was prepared from 5 mg of solid α66-80 crystallin peptide. The solution was stored in 40 mL aliquots at -20 ^{o}C. 10 mM stock solutions of host **MR-8S** was prepared from 5 mg of solid powder in water. The host solution was stored in 20 mL aliquots at -20 ^{o}C.

For the analysis of each individual molecule, 50 μM solutions were prepared by combining 5 mL of the 10 mM stock solutions with 955 mL of the ESI buffer in a microcentrifuge tube and vortexing for 1 minute. The ESI buffer consisted of 50% HPLC grade methanol, 49% water, and 1% acetic acid (%v).

For complexation study, equal parts of 10 mM peptide and 10 mM **MR-8S** was combined in a microcentrifuge tube, vortexed for 1 min and briefly centrifuged down. This yielded equimolar (5 mM: 5 mM) host-peptide. 2 mL were immediately portioned out into a separate centrifuge tube with 198 mL ESI buffer to dilute the concentration of each to 50 mM. This sample was analyzed with ESI-MS immediately, within 2 hours of preparing the solution.

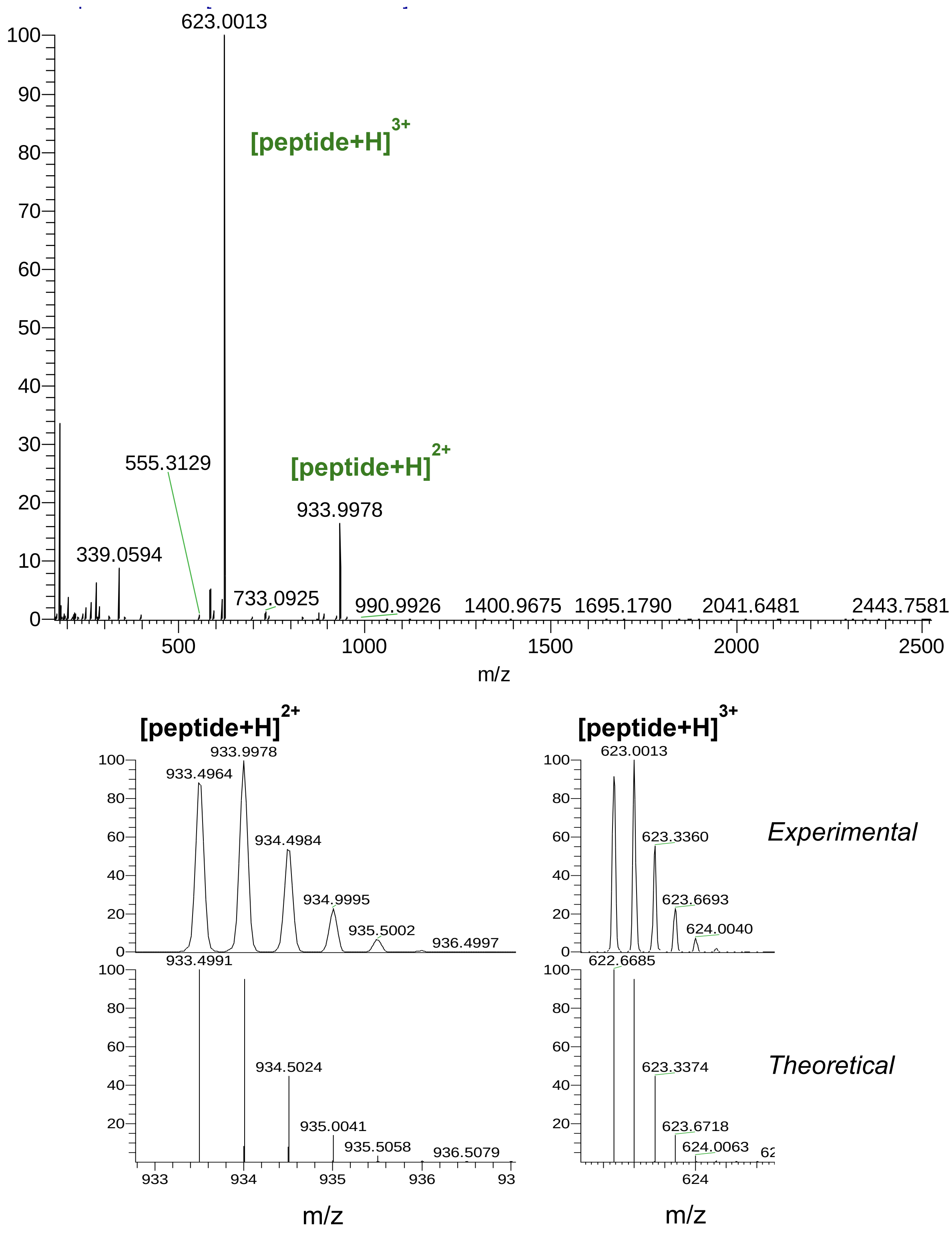

Figure S4: ESI mass spectrum of αA66-80-crystallin peptide (ESI buffer, positive ionization mode).

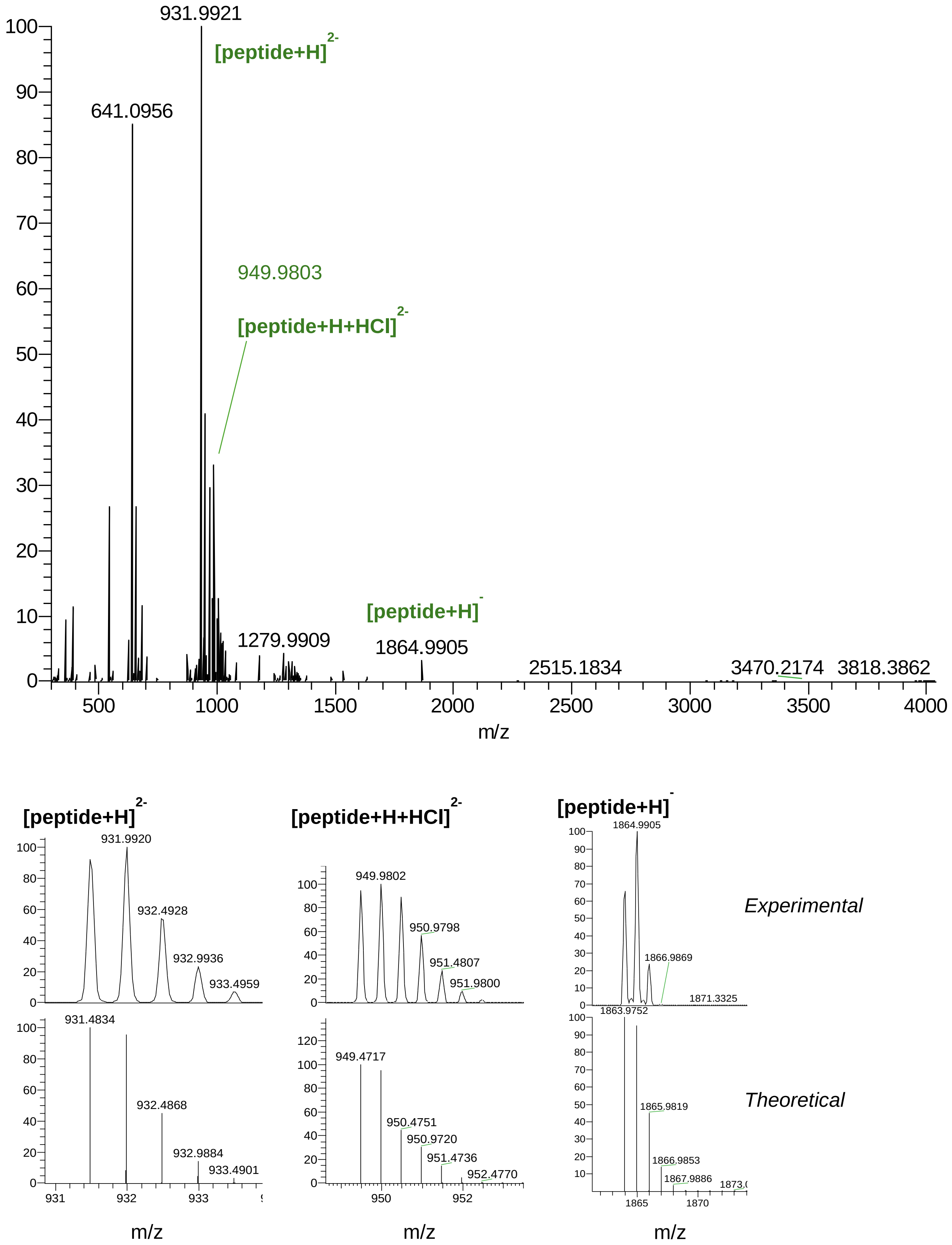

Figure S5: ESI mass spectrum of αA66-80-crystallin peptide (ESI buffer, negative ionization mode).

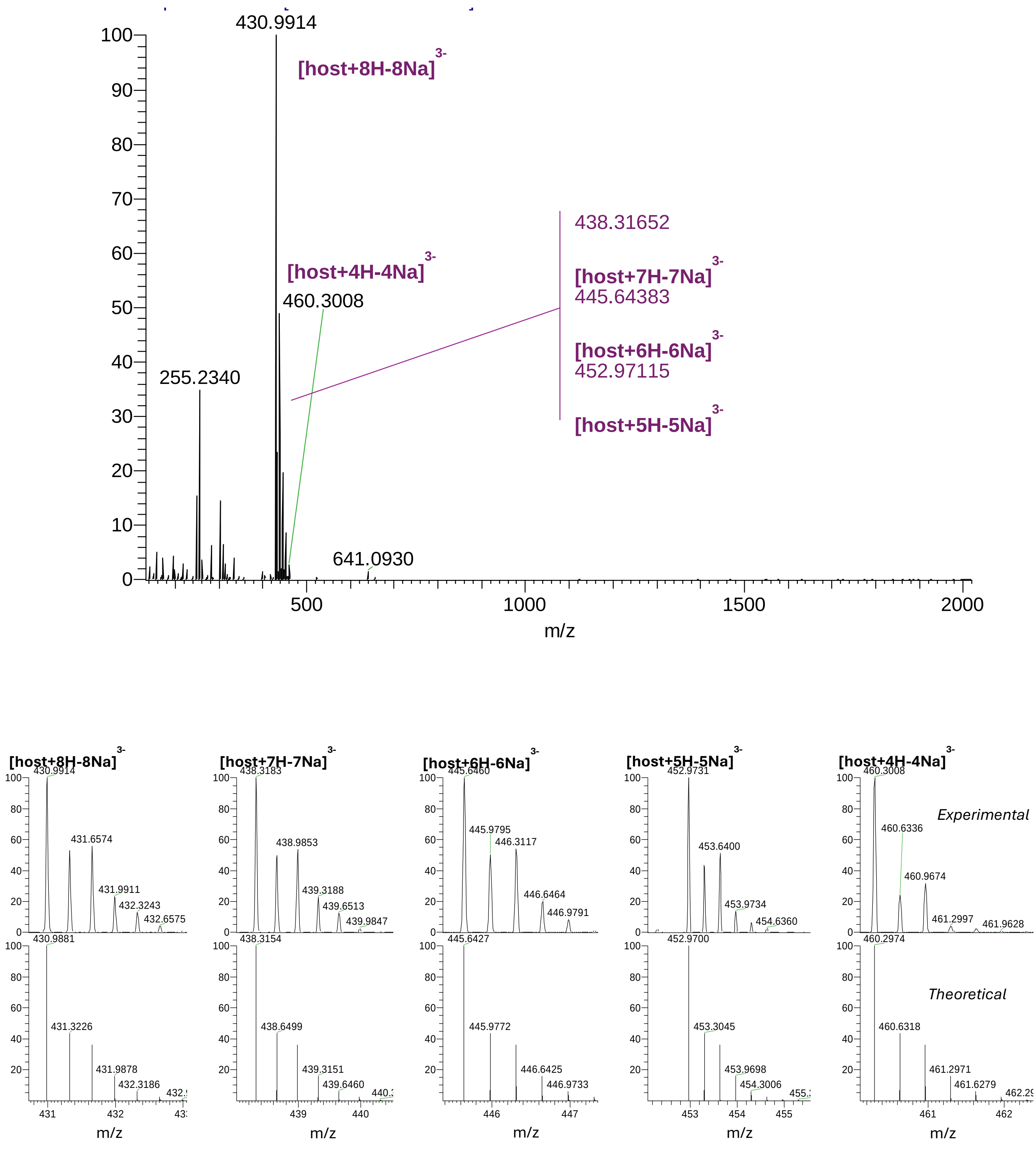

Figure S6: ESI mass spectrum of **MR-8S** macrocycle (ESI buffer, negative ionization mode).

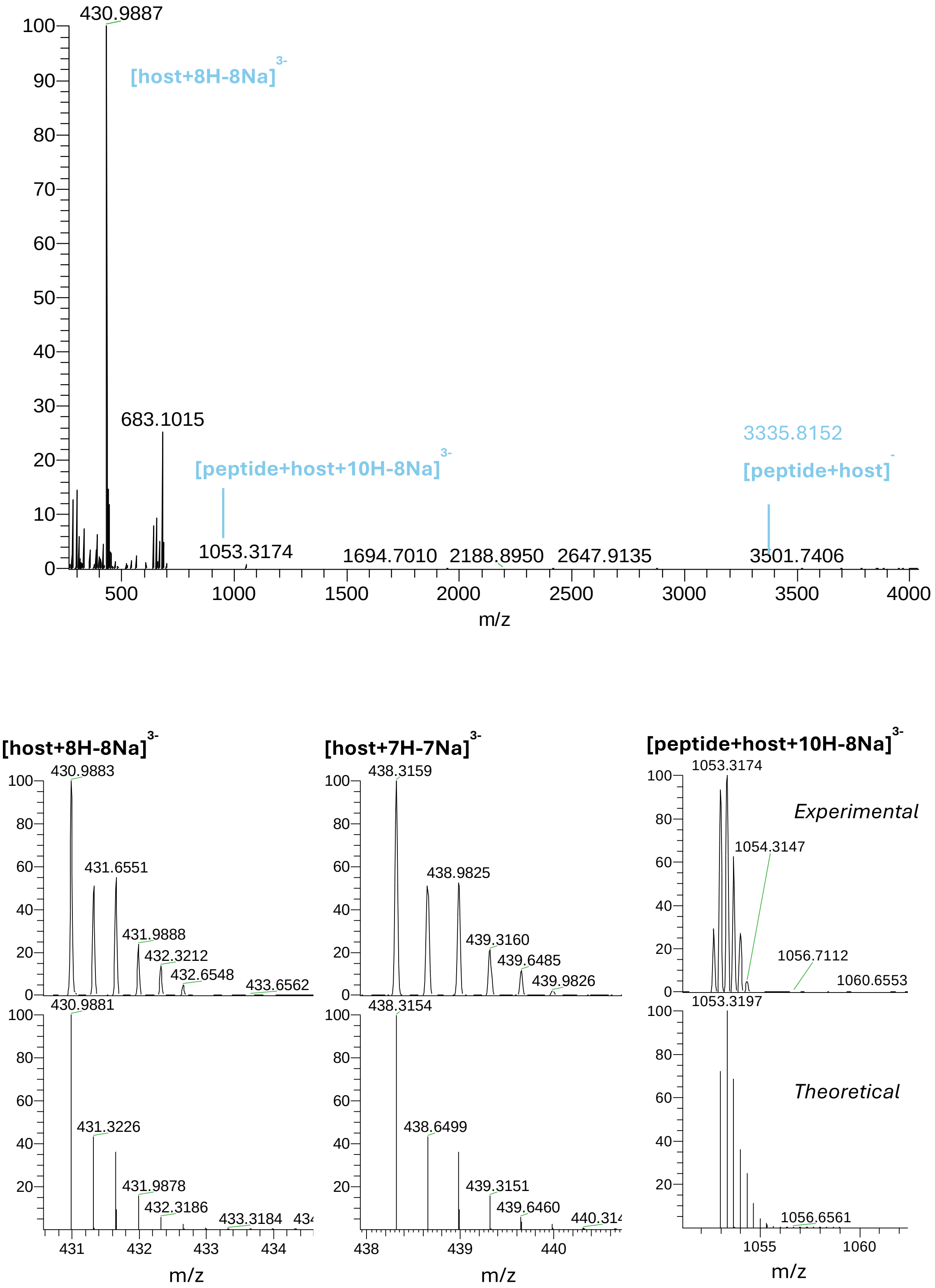

Figure S7: ESI mass spectrum of an equimolar mixture of **MR-8S** and αA66-80-crystallin peptide (ESI buffer, negative ionization mode).

III. Dynamic Light Scattering (DLS) Experiments

To assess the particle size distribution of the αA66-80-crystallin with **MR-8S**, pure solutions of α66-80-crystallin and the mixtures at 0.2, 0.4, 0.6, 0.8, 1.0, 2.0, 5.0, and 10.0 ratios were incubated at 37 °C in 10 mM Tris buffer (pH 7.4) for seven days. 1 mL of each solution was pipetted into a cuvette and measured using the Malvern zetasizer. Each experiment was done in triplicates. The size distribution and Z-averages were obtained and analyzed.

Table S1: A list of the Z-average sizes of crystallin in varying concentrations of **MR-8S** in 10 mM Tris buffer.

System	Z – Average
Pure αA66-80-crystallin	13293±1072
0.2 **MR-8S**:1 crystallin	7519±454
0.4 **MR-8S**:1 crystallin	4518±1051
0.6 **MR-8S**:1 crystallin	1885±186
0.8 **MR-8S**:1 crystallin	1537±264
1.0 **MR-8S**:1 crystallin	1483±15
2.0 **MR-8S**:1 crystallin	1482±36
5.0 **MR-8S**:1 crystallin	1320±194
10 **MR-8S**: 1 crystallin	767±25

Table S2: A list of the Z-average sizes of crystallin in varying concentrations of **MR-8S** in 100%$^{v}/_{v}$ aqueous humor.

System	Z – Average
Pure αA66-80-crystallin	10639±1520
0.25 **MR-8S**:1 crystallin	5987±1111
0.50 **MR-8S**:1 crystallin	4083±1942
1.0 **MR-8S**:1 crystallin	2118±286
5.0 **MR-8S**:1 crystallin	869±162
10.0 **MR-8S**:1 crystallin	718±121

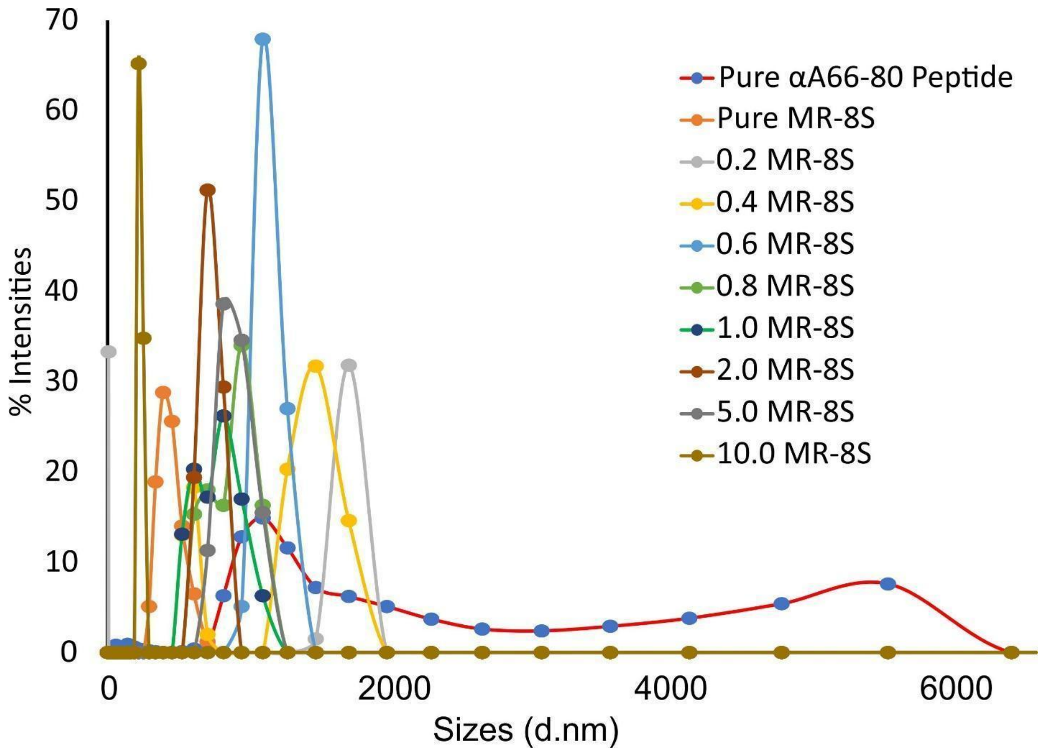

Figure S8. A scatter plot illustrating the size distributions of αA66-80-crystallin peptide in varying concentrations of **MR-8S** in 10 mM Tris buffer.

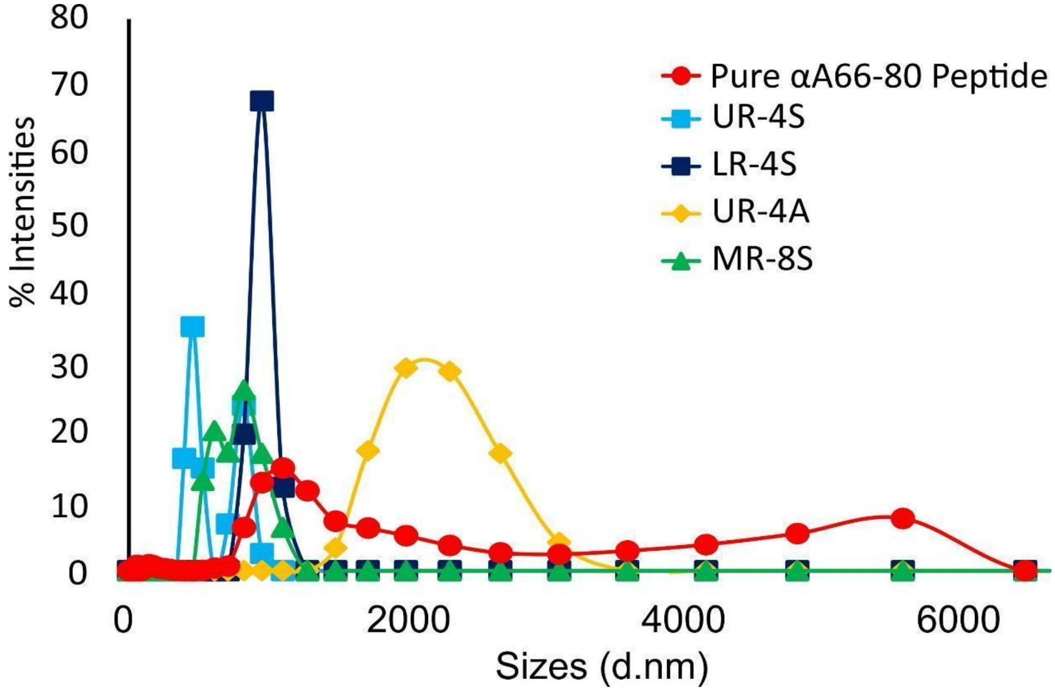

Figure S9. A scatter plot illustrating the size distributions of αA66-80-crystallin in equimolar concentrations with **LR-4S**, **UR-4S**, **UR-4A** and **MR-8S** in 10 mM Tris buffer.

IV. Proteostat Fluorescence Aggregation Assay

Receptor:peptide concentrations were prepared by pipetting 1 mL of 536 μM freshly prepared crystallin peptide solution in 10mM Tris buffer. 1 mL of a concentrated solution of the receptor is added to the solution to make 0.2:1, 0.4:1, 0.6:1, 0.8:1, 1:1, 5:1 and 10:1 ratio that is then incubated at 37°C for 7 days. A control sample of peptide alone was also incubated for same duration. After the incubation period, a 50 μL aliquot of proteostat dye is added into 800 μL of each sample solution. Prior to transferring each sample into the microplate wells, positive and negative controls were prepared and added to the plate in order to ensure sample integrity and to verify that there was minimal contribution of free proteostat dye solution to the level of fluorescence intensity measured. Fluorescence readings were done at 37 °C, emission of 550 nM and excitation of 600 nM to monitor the extent of fibrils and filaments formed. All the measurements were done in triplicates. A calibration curve of log of concentration against percent inhibition of aggregation was plotted (Figure S10) and IC50 calculated.

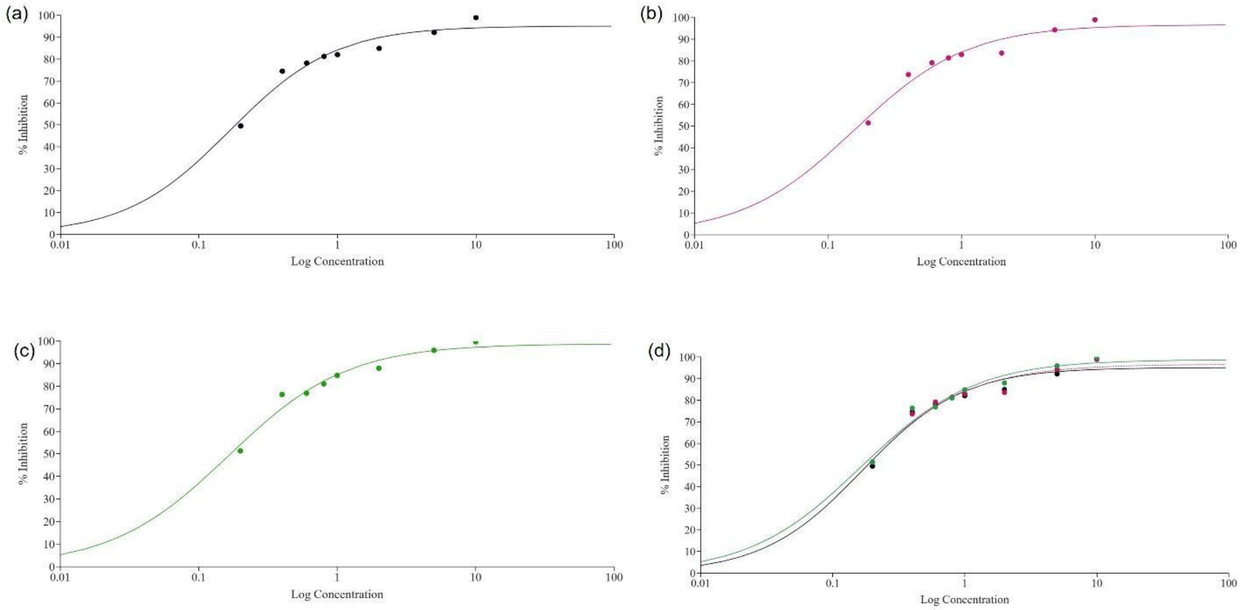

Figure S10. Percent inhibition of αA66-80-crystallin peptide aggregates in increasing concentrations of **MR-8S** in 10 mM Tris buffer. The experiments were done in triplicates. (a) dataset 1, b) dataset 2, c) dataset 3 and d) overlay of datasets 1-3.

V. Transmission Electron Microscopy (TEM) Imaging

αA66-80 crystallin peptide was mixed with **MR-8S** in a 1:1 molar ratio to a final equimolar concentration of 5 mM in DI water. Pure crystallin and pure host 10 were also prepared individually to final concentrations of 5 mM in DI water. Samples were incubated for 7 days at 37°C. Samples for crystallin, **MR-8S**, and the peptide-host mix were also freshly made on day 7 to compare to the aged samples. 10 μL aliquots were taken from each sample, loaded onto a Formvar-carbon coated 200 mesh nickel grids, and allowed to absorb for 15 minutes in ambient light. The grids were washed with 10 μL of DI water and blotted dry with filter paper. 5 μL of 2% glutaraldehyde was then loaded onto each grid for 5 minutes, blotted dry, and washed with DI

water. Each grid was then stained with 5 μL of 1% uranyl acetate for 5 minutes, blotted dry, and washed a final time with DI water. Images were acquired from the TEM using a magnification range of 3000-10,000x. Duplicate grids were made for each sample.

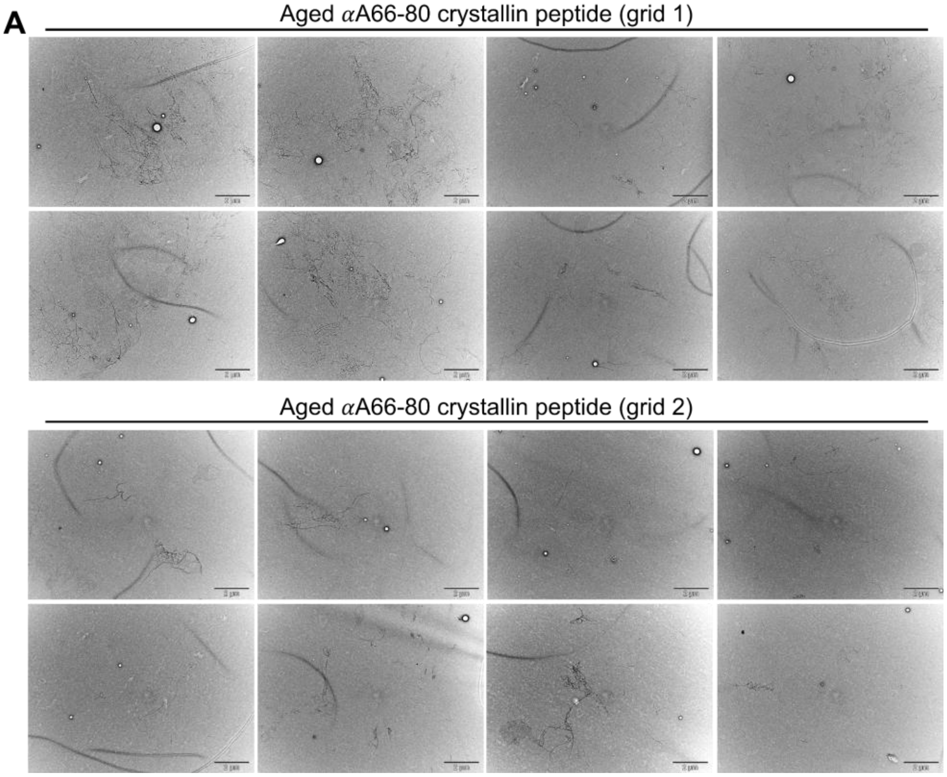

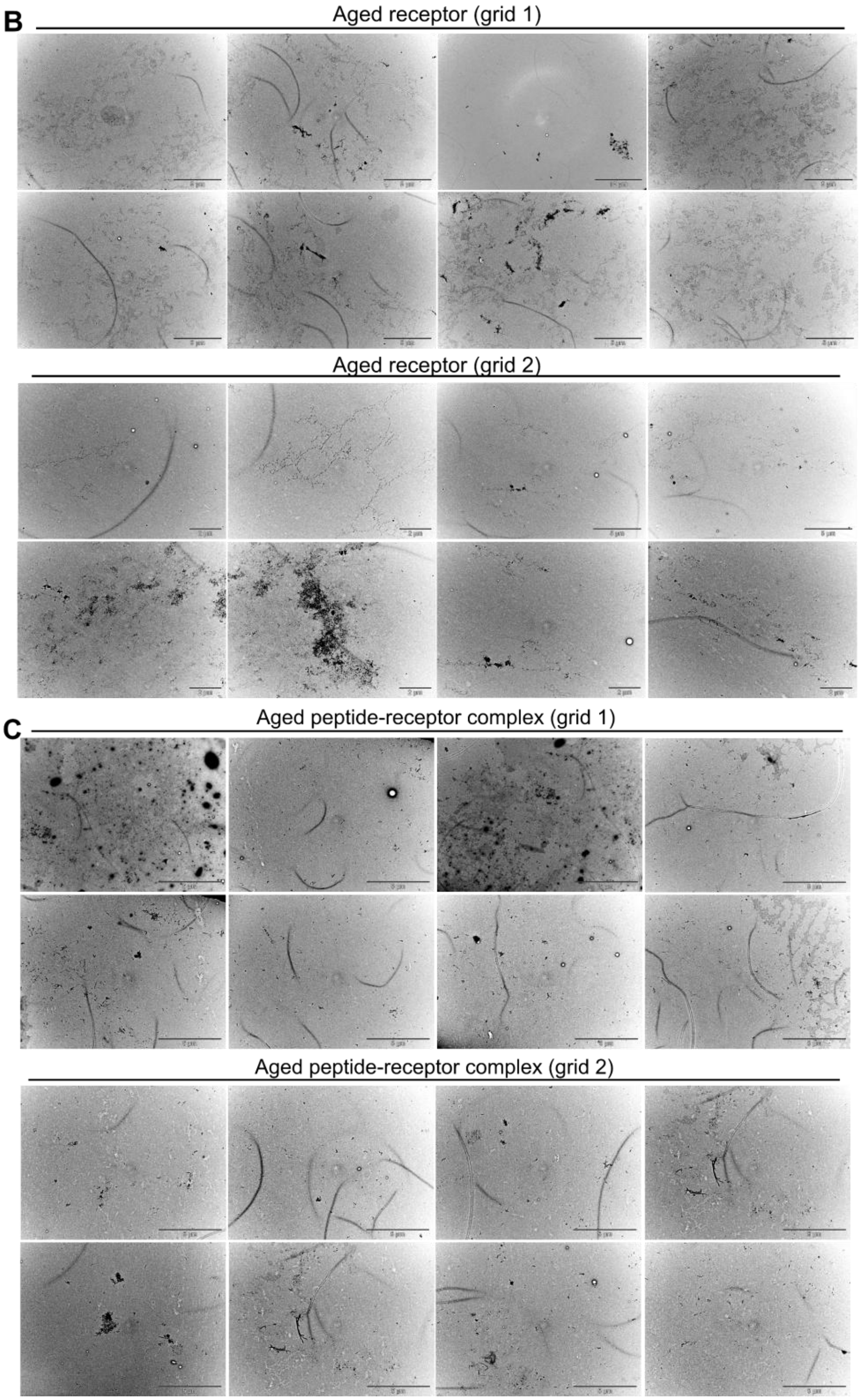

Figure S11. TEM images of aged (t = 7 days) (A) αA66-80 crystallin peptide, (B) receptor **MR-8S**, and (C) complexation of peptide:**MR-8S** (1:1 molar concentration). Individual sample replicates were analyzed on separate formvar carbon-coated nickel mesh TEM grids. Unless

otherwise noted, the samples were incubated at 37°C for 7 days prior to imaging. Each image contains a separate scale bar and the magnification range is listed in the TEM Methods section.

VI. ^{1}H NMR Spectroscopy

For sample preparation, stock solutions of the **MR-8S** (5 mM) and amino acids (5 mM) were prepared in D_2O. For pure sample measurements, 250 µL of the 5 mM stock solution was measured into NMR tube and diluted with 250 µL of D_2O. To measure possible binding interactions, 250 µL of 5 mM **MR-8S** and 250 µL of 5 mM amino acid solution were measured into an NMR sample tube to give a resulting mixture of 2.5 mM of both samples. This was repeated using **MR-8S** and the amino acids. For a 1:1 (**MR-8S** ·amino acid) mixture, 250 µL of **MR-8S** and 250 µL of amino acid were pipetted into a clean NMR tube, making a 1:1 equimolar sample with 2.5 mM concentration of each component in the mixture. ^{1}H NMR was carried out on 400MHz Bruker Spectrometer.

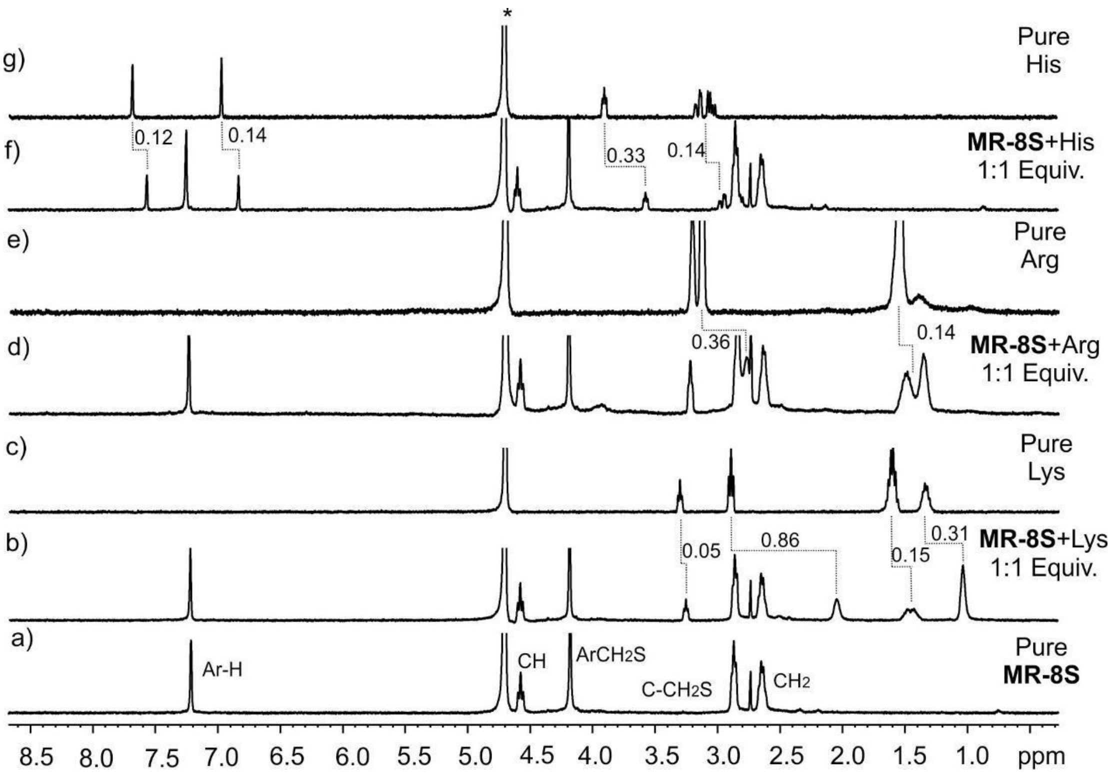

Figure S12. Sections of the ^{1}H NMR spectra in D_2O at 298 K of receptor **MR-8S** and several cationic amino acids of the **αA66-80** crystallin peptide. Pure samples: (a) **MR-8S**, (c) Lysine, (e) Arginine, and (g) histidine. Equimolar mixtures of: (b) **MR-8S**+Lysine, (d) **MR-8S**+Arginine, and (f) **MR-8S**+Histidine. The dashed lines indicate the signal changes in ppm. The star (*) represents the residual D_2O solvent.

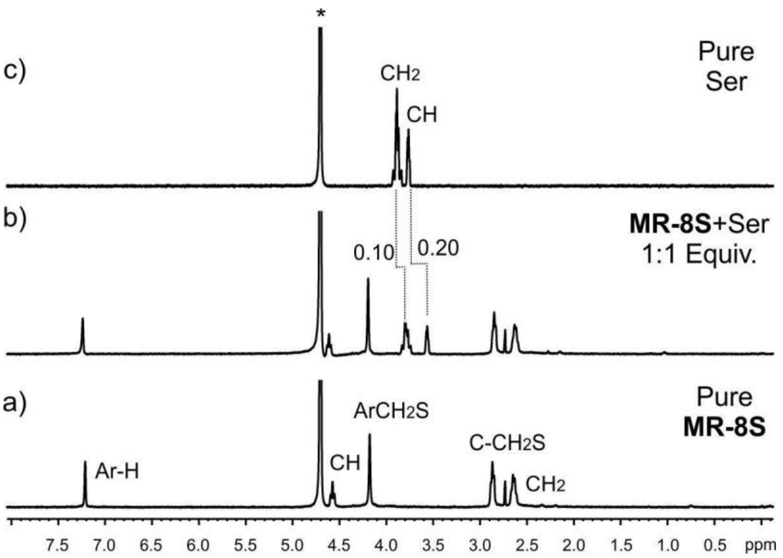

Figure S13. Sections of the ^{1}H NMR spectra in D_2O at 298 K of **MR-8S** and polar amino acids (a) receptor **MR-8S**, (c) Serine, and equimolar mixture of (b) receptor **MR-8S**+Serine. The dashed arrows indicate the signal changes in ppm. Star represents the residual D_2O solvent.

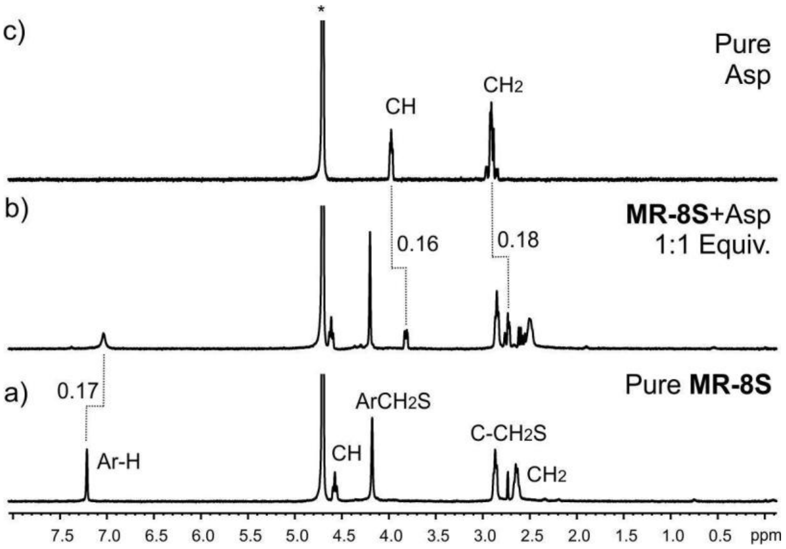

Figure S14. Sections of the ^{1}H NMR spectra in D_2O at 298 K of **MR-8S** and anionic amino acids (a) receptor **MR-8S**, (c) Aspartic acid, and equimolar mixture of (b) receptor **MR-8S**+Aspartic acid. The dashed arrows indicate the signal changes in ppm. Star represents the residual D_2O solvent

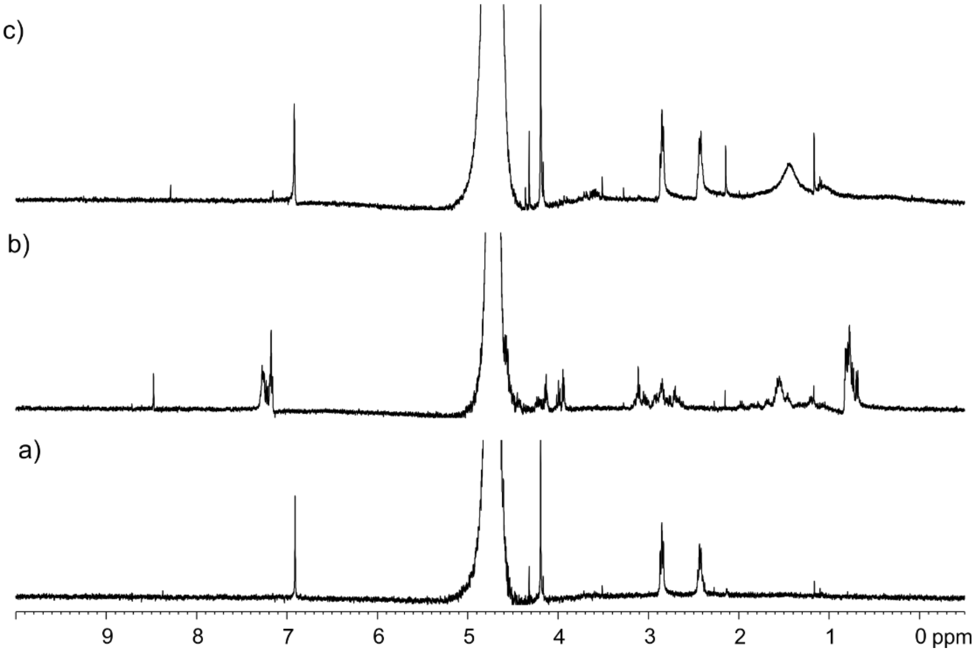

Figure S15. ^{1}H NMR spectra in D_2O at 298 K of receptor **MR-8S** and αA66-80 crystallin peptide. Pure samples: (a) **MR-8S**, (c) αA66-80 peptide. (b) Equimolar mixture of **MR-8S**+αA66-80 peptide.

VII. Isothermal Titration Calorimetry (ITC)

The ITC experiment was carried out by filling the sample cell with **MR-8S** (1 mM), filling the syringe with the amino acid (10 mM), and titrating via computer-automated injector at 310 K. Blank titrations into plain Tris buffer (10 mM) were also performed and subtracted from the corresponding titration to remove any effect from the heats of dilution from the titrant. Isotherms and thermodynamic parameters from an independent and a multiple site fitting model (Ka, ΔH and ΔS) were obtained using the NaNoAnalyze software. Gibbs' free energy ΔG was subsequently calculated at 310 K and recorded.

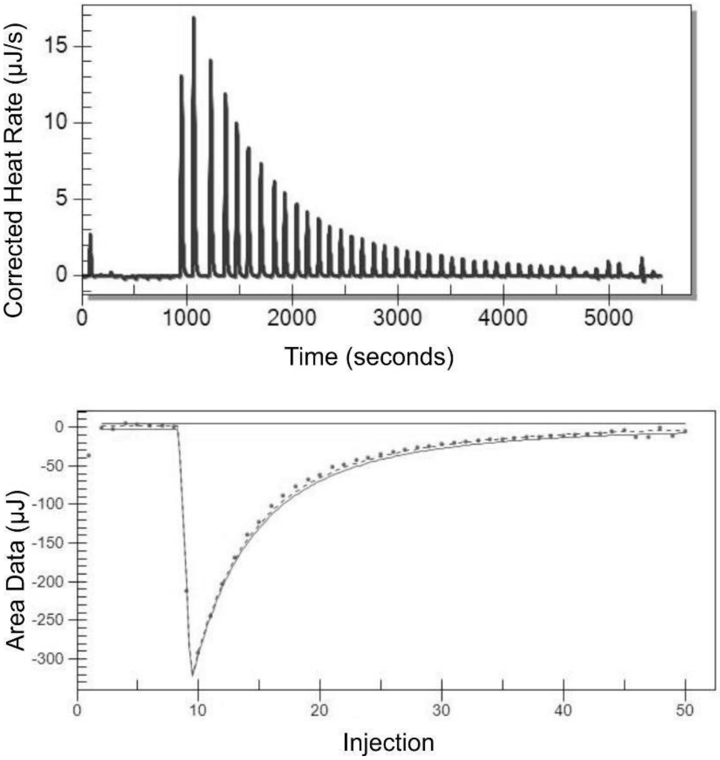

Figure S16. ITC traces of the titration into **MR-8S** with Lysine in 10mM TRIS buffer at 310 K. The data were fitted into a two-site binding model.

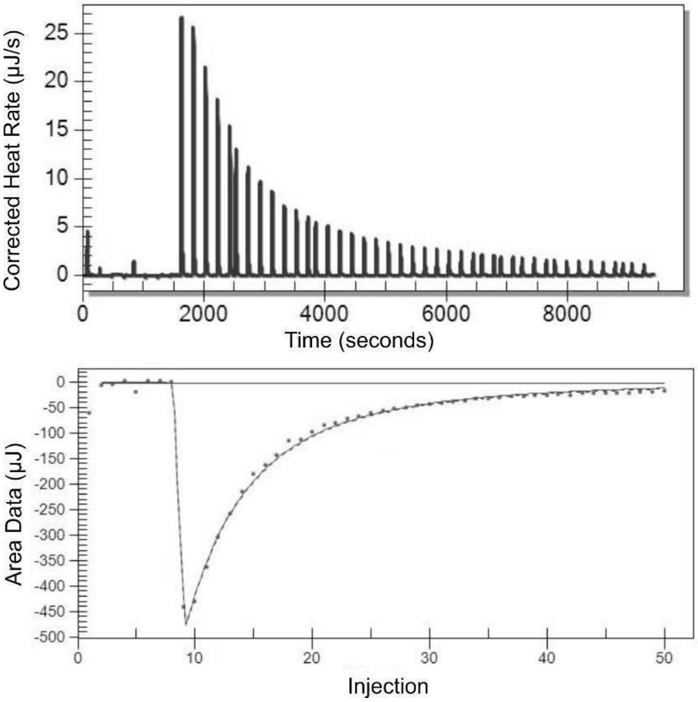

Figure S17. ITC traces of the titration into **MR-8S** with Arginine in 10mM TRIS buffer at 310 K. The data were fitted into a two-site binding model.

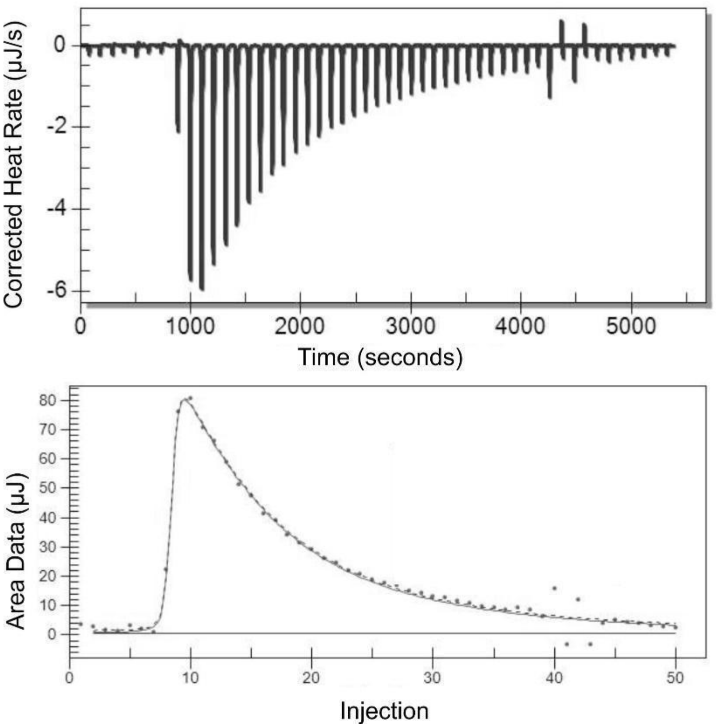

Figure S18. ITC traces of the titration into **MR-8S** with Histidine in 10mM TRIS buffer at 310 K. The data were fitted into a two-site binding model.

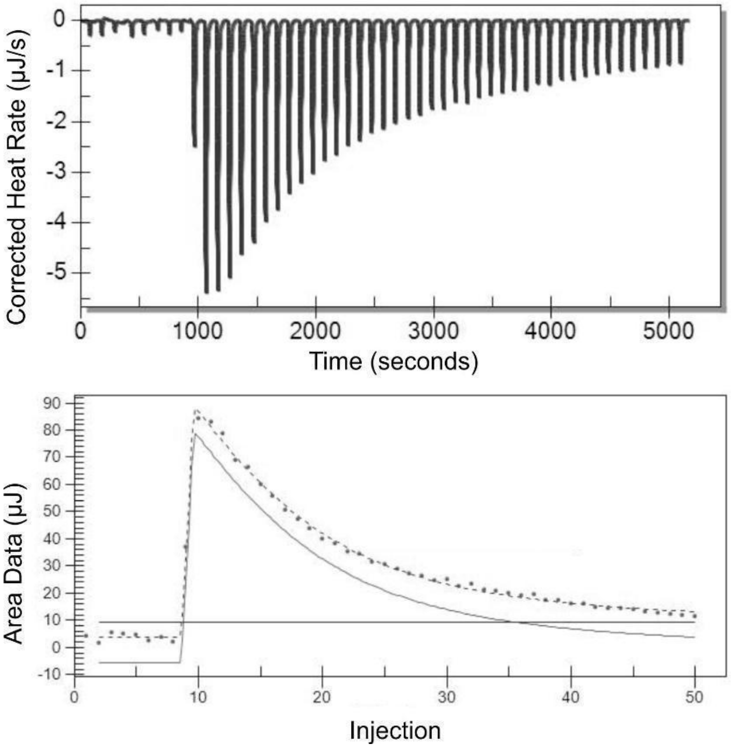

Figure S19. ITC traces of the titration into **MR-8S** with Valine in 10mM TRIS buffer at 310 K. The data were fitted into a two-site binding model.

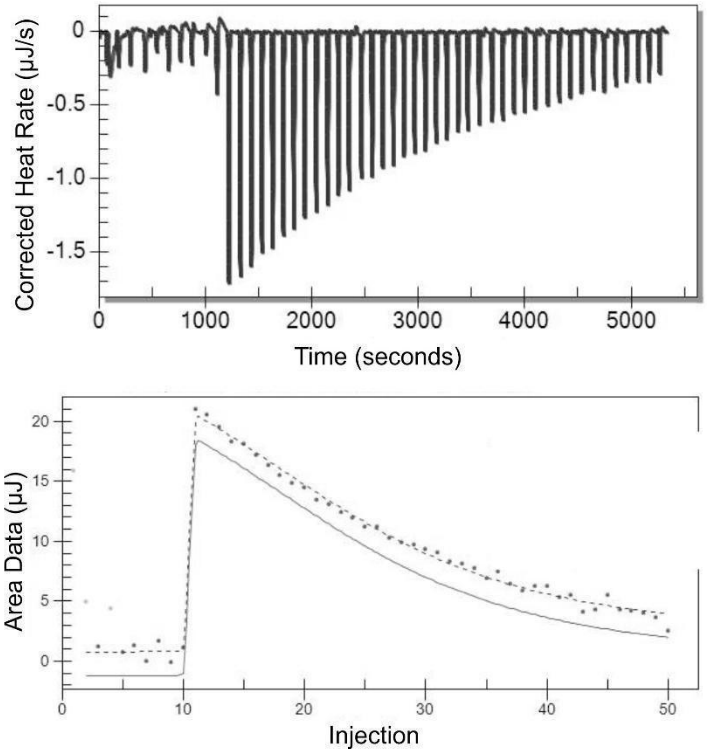

Figure S20. ITC traces of the titration into **MR-8S** with Isoleucine in 10mM TRIS buffer at 310 K. The data were fitted into a two-site binding model.

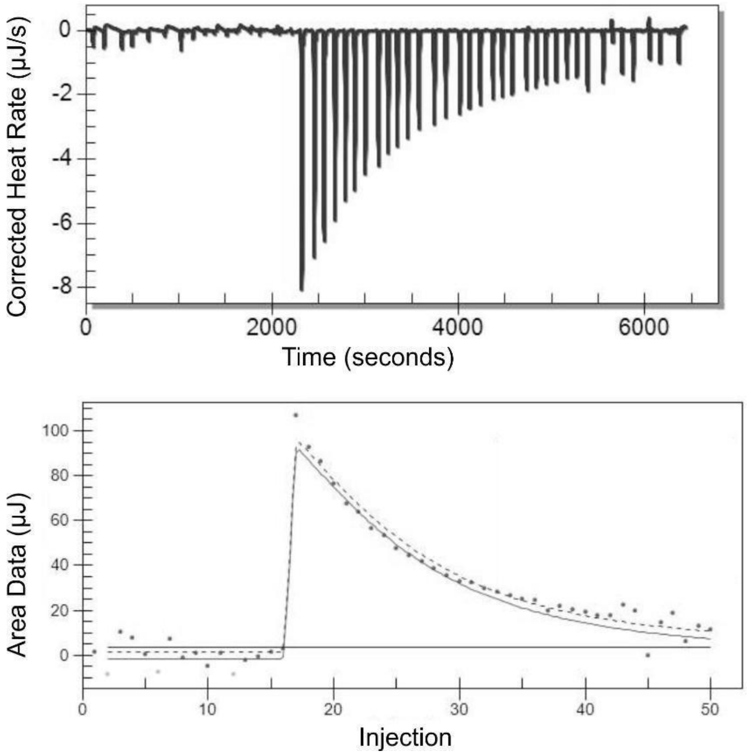

Figure S21. ITC traces of the titration into **MR-8S** with Leucine in 10mM TRIS buffer at 310 K. The data were fitted into a two-site binding model.

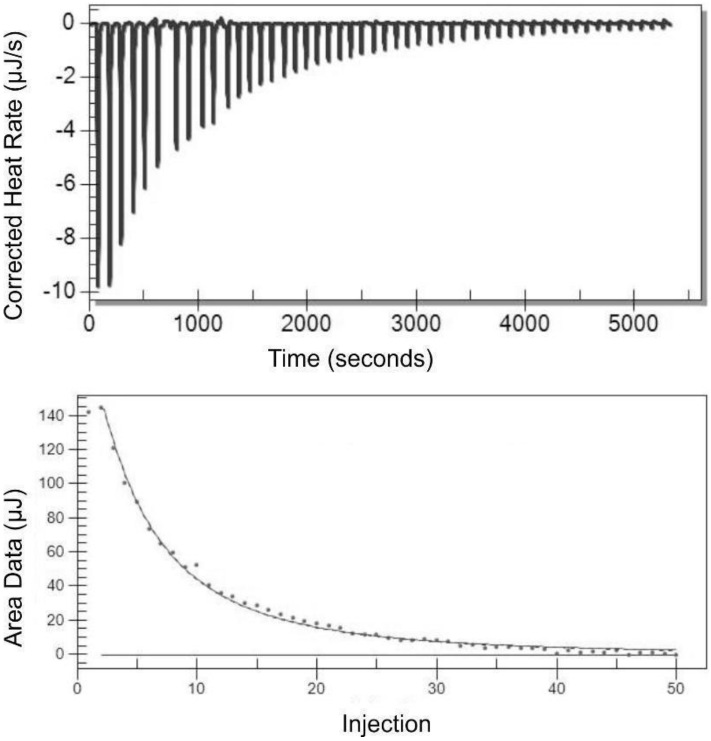

Figure S22. ITC traces of the titration into **MR-8S** with Phenylalanine in 10mM TRIS buffer at 310 K. The data were fitted into a one-site binding model.

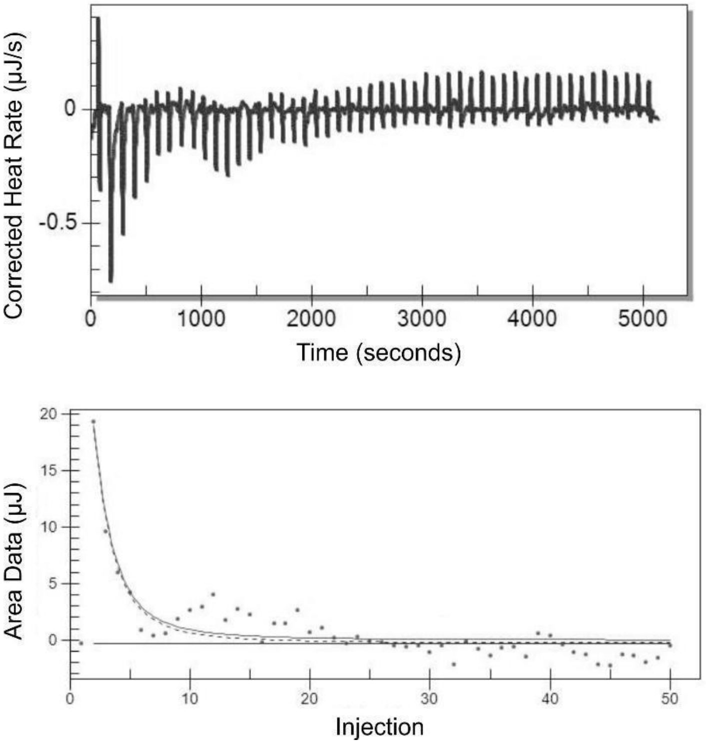

Figure S23. ITC traces of the titration into **MR-8S** with Serine in 10mM TRIS buffer at 310 K. The data were fitted into a two-site binding model.

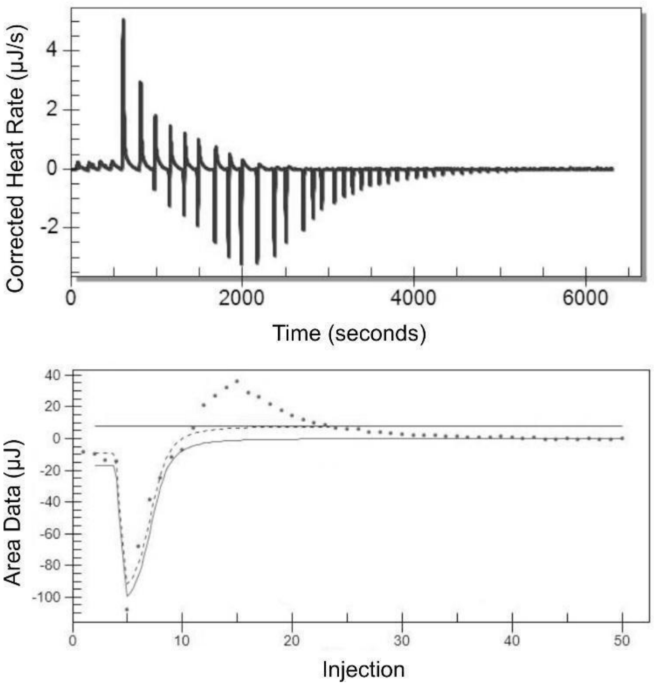

Figure S24. ITC traces of the titration into **MR-8S** with Aspartic Acid in 10mM TRIS buffer at 310 K. The data were fitted into a two-site binding model.

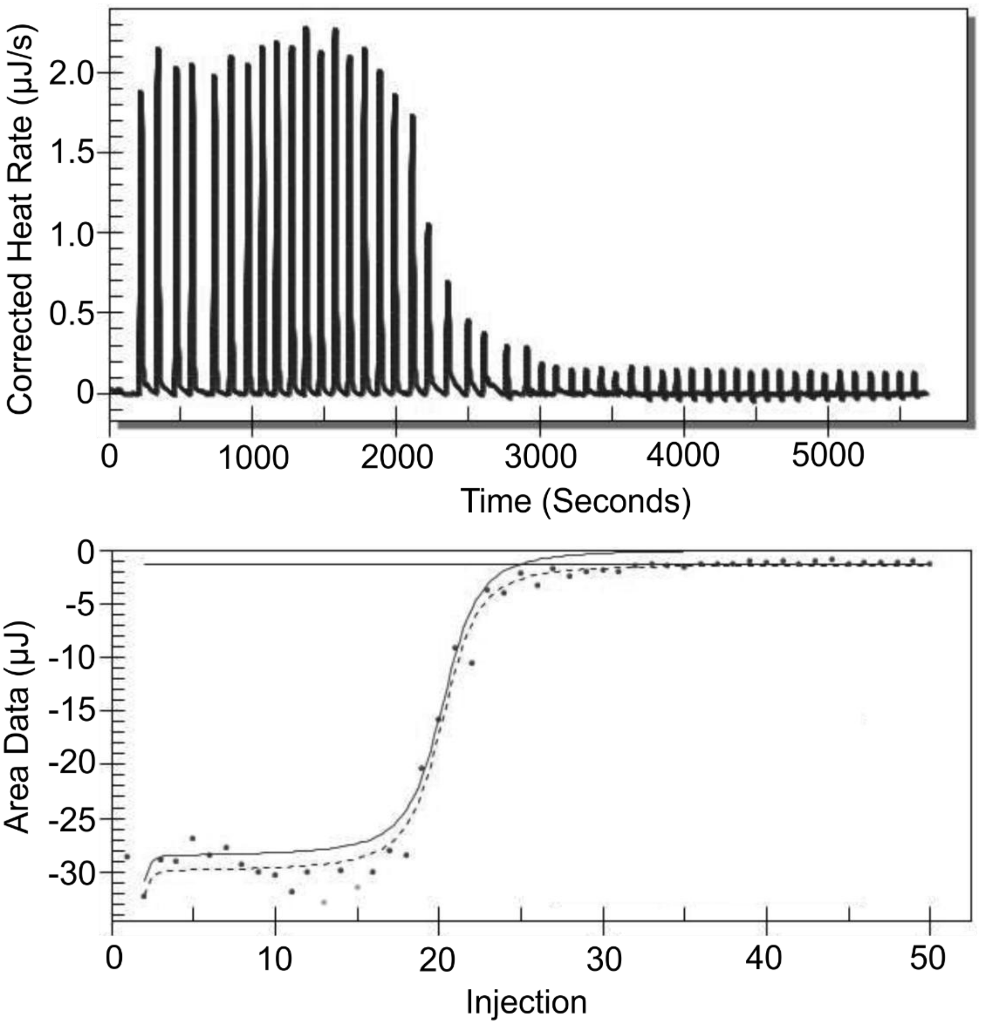

Figure S25. ITC traces of the titration into **MR-8S** with αA66-80 crystallin peptide in 10mM TRIS buffer at 310 K. The data were fitted into a two-site sequential binding model.

VIII. Structure Preparations

We began with the PDB structure of human αA-crystallin (PDB ID: 6T1R)[2], obtained from the RCSB Protein Data Bank, which represents a sixteen-mer assembly. Using Chimera[3], all extra chains were removed, and chain A was retained for further analysis. This chain was submitted to the H++ web server[4–6] to assign protonation states at physiological pH 7.4. The resulting protonated structure was then trimmed to isolate the αA66–80 crystallin peptide, as shown in Figure 7 in the main manuscript. All Asp residues in the trimmed peptide were deprotonated, all Lys residues were protonated, and the sole His residue was protonated at Nδ. To prepare the peptide for molecular dynamics (MD) simulations, the N- and C-terminal ends were chemically capped acetylation (ACE) at the N-terminus and amidation (NME) at the C-terminus to neutralize terminal charges and maintain structural stability during the simulation.

To investigate the aggregation mechanism, two systems were constructed. In the first, two peptide chains were placed in the same simulation box to allow dimerization, and in the second, four peptide chains were used to study tetramer formation. These initial configurations were generated using the N- and C-terminal capped peptide as input for Packmol[7], with a 2.0 Å tolerance and a 40 Å cubic box. The Packmol output structures (shown in Figure 8 of the main manuscript as randomly placed dimer and tetramer) were then prepared for MD simulations. The *tleap* program of Amber24[8,9] was employed to solvate the peptide dimer and tetramer in cubic boxes with a 10 Å buffer of TIP3P[10] water (4,262 water molecules for the dimer and 6,251 for the tetramer). NaCl ions were added to achieve a physiological salt concentration of 0.15 M, resulting in total system sizes of 13,366 atoms for the dimer and 19,897 atoms for the tetramer. The Amber ff19SB[11,12] force field was used for the peptide.

To explore the interactions between resorcinarenes and the peptide, molecular docking was performed prior to MD simulations. The structures of **UR-4S**, **LR-4S**, **MR-8S**, and **UR-4A** were first geometry-optimized using Density Functional Theory (DFT) at the B3LYP[13]/6-31G(d,p)[14,15] level with the Gaussian 16 quantum chemistry package.[16] The optimized structures are shown in Figure 1 in main manuscript and were subsequently used as input geometries for the docking calculations. Docking was carried out using the Smina software.[17] Prior to docking, the PDB coordinates of both the peptide and the resorcinarenes were converted to the PDBQT format using the Open Babel package,[18] with assignment of partial charges ("Q") and AutoDock 4 (AD4) atom types ("T"). A cubic docking grid box of 40 Å was defined to ensure exhaustive conformational sampling and unbiased identification of favorable binding poses. The results of the docking studied are shown in Supplementary Information in section XI. The best docked poses were used as starting structures for MD simulations.

The charges and parameters of the resorcinarenes, including **MR-8S**, were generated using the *Antechamber* program of Amber24[8,9] with the AM1-BCC charge scheme and GAFF2[19] without any manual adjustments, including for the sulfonate groups. Resorcinarenes were simulated in their experimentally relevant charge states: **UR-4S** and **LR-4S** (−4), **UR-4A** (+4), and **MR-8S** (−8). No comparisons with higher-level DFT-derived charges were performed, as the primary goal is to compare the relative efficacy of resorcinarenes in shielding the aggregation-prone hydrophobic core of the αA66–80 peptide rather than obtaining absolute binding free energies.

For each peptide–resorcinarene complex, *tleap*[8,9] was used to solvate the system in a cubic box with a 10 Å TIP3P[10] water buffer, resulting in 3,051 water molecules for the peptide–**UR-4S** complex, 3,054 for peptide-**LR-4S**, 3,035 for peptide-**MR-8S** and 3,014 for peptide-**UR-4A** complex. NaCl ions were added to achieve a physiological salt concentration of 0.15 M and neutralize the charge created due to poly-ionic resorcinarenes, yielding total system sizes of 9,563 atoms for the peptide-**UR-4S** complex, 9,560 for peptide-**LR-4S**, 9,531 for peptide-**MR-8S** and 9,520 for peptide-**UR-4A**. The Amber ff19SB[11,12] force field was applied to the peptides.

IX. Details of Molecular Dynamics (MD) Simulations

All simulations were performed using the *pmemd.cuda*[20,21] implementation of the AMBER24[8,9] software package. To ensure proper relaxation, the systems underwent a multi-stage explicit solvent equilibration protocol. First, the added water molecules were minimized for 5000 steps (steepest descent followed by conjugate gradient) while restraining the solute with a force constant of 100 kcal mol^{-1} $Å^{-2}$. This was followed by three rounds of MD at constant pressure and 298K with progressively decreasing restraints. In the first round, the system was heated from 100K to 298K over 1 ns with peptide and resorcinarene restrained at 100 kcal mol^{-1} $Å^{-2}$. In the second round, box density was equilibrated for 1 ns at 298K while restraining the peptide backbone and ions (100 kcal mol^{-1} $Å^{-2}$), allowing other atoms to relax. In the third round, the system was equilibrated for 1 ns with backbone restraints reduced to 10 kcal mol^{-1} $Å^{-2}$. The system was then minimized for 1000 steps with 10 kcal mol^{-1} $Å^{-2}$ backbone restraints, followed by three additional 1 ns equilibration rounds with restraints of 10, 1.0, and 0.1 kcal mol^{-1} $Å^{-2}$, respectively. A final 1 ns equilibration at constant pressure and 298K with no restraints allowed the peptide–ligand complex to fully relax. The fully relaxed structure was equilibrated through a 20 ns production run using a 2fs time step, with all bonds involving hydrogen atoms constrained via the *SHAKE*[22] algorithm. Long-range electrostatic interactions were treated using a 9 Å cutoff, and simulations were carried out in the NPT ensemble at 298 K, employing a Langevin thermostat[23] and a Berendsen barostat[24]. Following this, the production run was extended to 100 ns with three independent replicas, which served as the final production simulations. The analysis were carried out on the final 100 ns of the trajectories, and residue-wise interaction energies were determined using the linear interaction energy (lie) modules available in *cpptraj*[25] module of AMBER24[8,9]. To enable a comparable assessment of core shielding using SASA, the uncomplexed αA66–80 peptide monomer was simulated using the same MD protocol in the replicates of three.

Contact Analysis

Intermolecular contact analysis was carried out using the cpptraj module[25] of the AMBER24[8,9] package to quantify ion–macrocycle interactions as well as peptide aggregation and dissociation behavior across all molecular dynamics trajectories. A distance cutoff of 3.5 Å was employed for all contact calculations, and both native and nonnative contacts were included to obtain a comprehensive description of transient interaction networks.

Ion–macrocycle contacts between Na^+/Cl^- ions and the resorcinarene systems were analyzed under 0.15 M NaCl conditions to evaluate charge-dependent ion association and electrostatic screening effects. In parallel, inter-peptide contact analysis was performed for both dimeric and tetrameric αA66–80 peptide assemblies to monitor aggregation stability and **MR-8S**–induced dissociation behavior. For the dimer systems, contacts between hydrophobic-core residues (F71–L75) of the two peptide chains were tracked to quantify changes in peptide–peptide interactions in both the absence and presence of **MR-8S**. For the tetramer systems in the absence of **MR-8S**, contacts between the hydrophobic-core residues of chains A, B, and C were computed with respect to the core residues of chain D to characterize the stability of the aggregated assembly. In the presence

of **MR-8S**, the corresponding analysis was performed by monitoring contacts between the core residues of chains A, C, and D relative to chain B, which reflects progressive disruption and dissociation of the tetrameric assembly. Time-resolved contact profiles were evaluated across all replicas and averaged trajectories to ensure statistical consistency and reproducibility of the observed interaction trends.

X. Investigation of the αA66–80-Crystallin Peptide Aggregation Mechanism

X.I Trajectory analysis for αA66–80-Crystallin Peptide dimer formation

X.I.I Replica-wise RoG analysis for αA66–80-Crystallin Peptide dimer formation

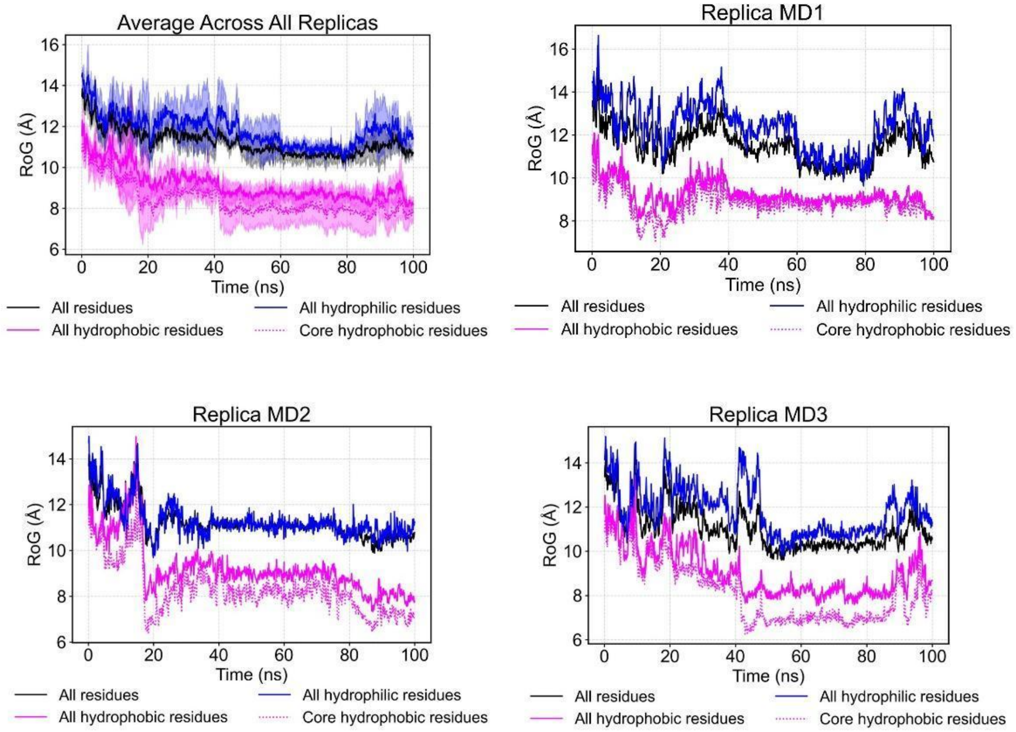

Figure S26. Radius of gyration (**RoG**) analysis of **αA66–80** crystallin peptide **dimer** formation. The plot shows average values across all replicas with corresponding standard deviations, followed by results from individual replicas.

X.I.II Replica-wise SASA analysis for αA66–80-Crystallin Peptide dimer formation

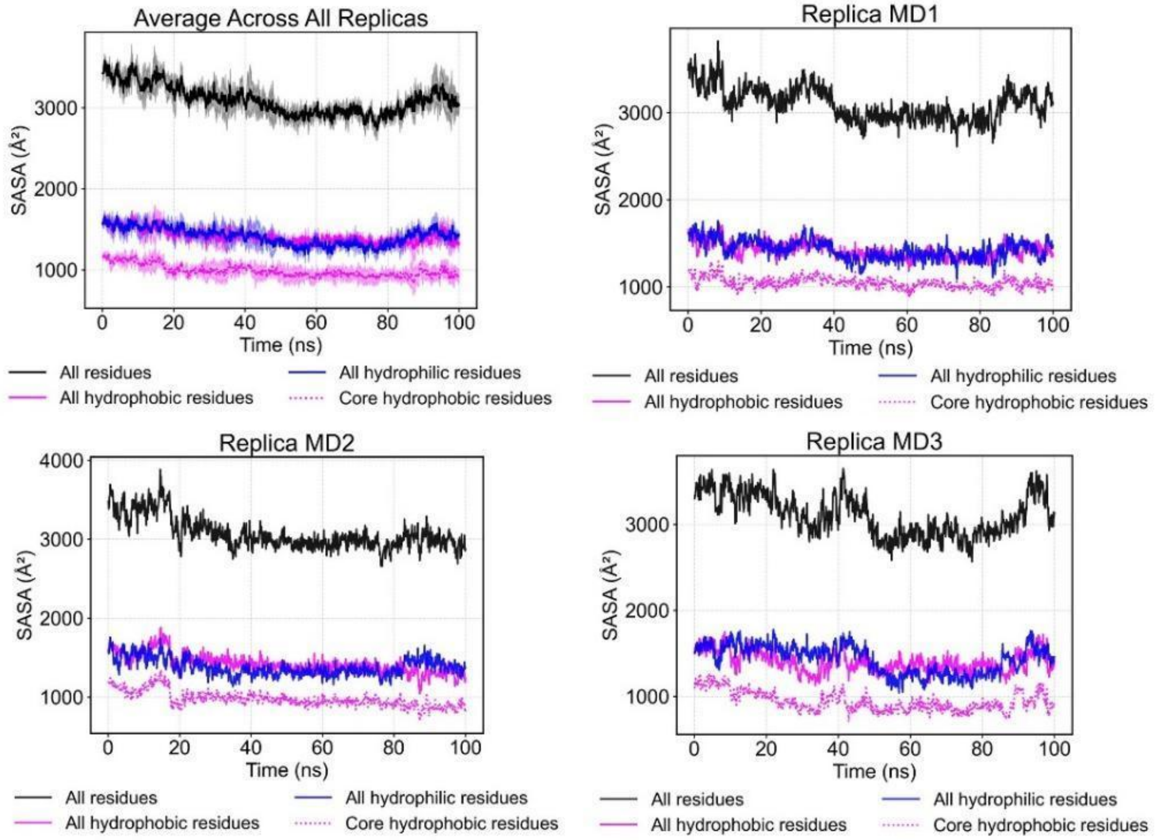

Figure S27. Solvent Accessible Surface Area (**SASA**) analysis of **αA66–80** crystallin peptide **dimer** formation. The plot shows average values across all replicas with corresponding standard deviations, followed by results from individual replicas.

X.I.III Replica-wise RMSF analysis for αA66–80-Crystallin Peptide dimer formation

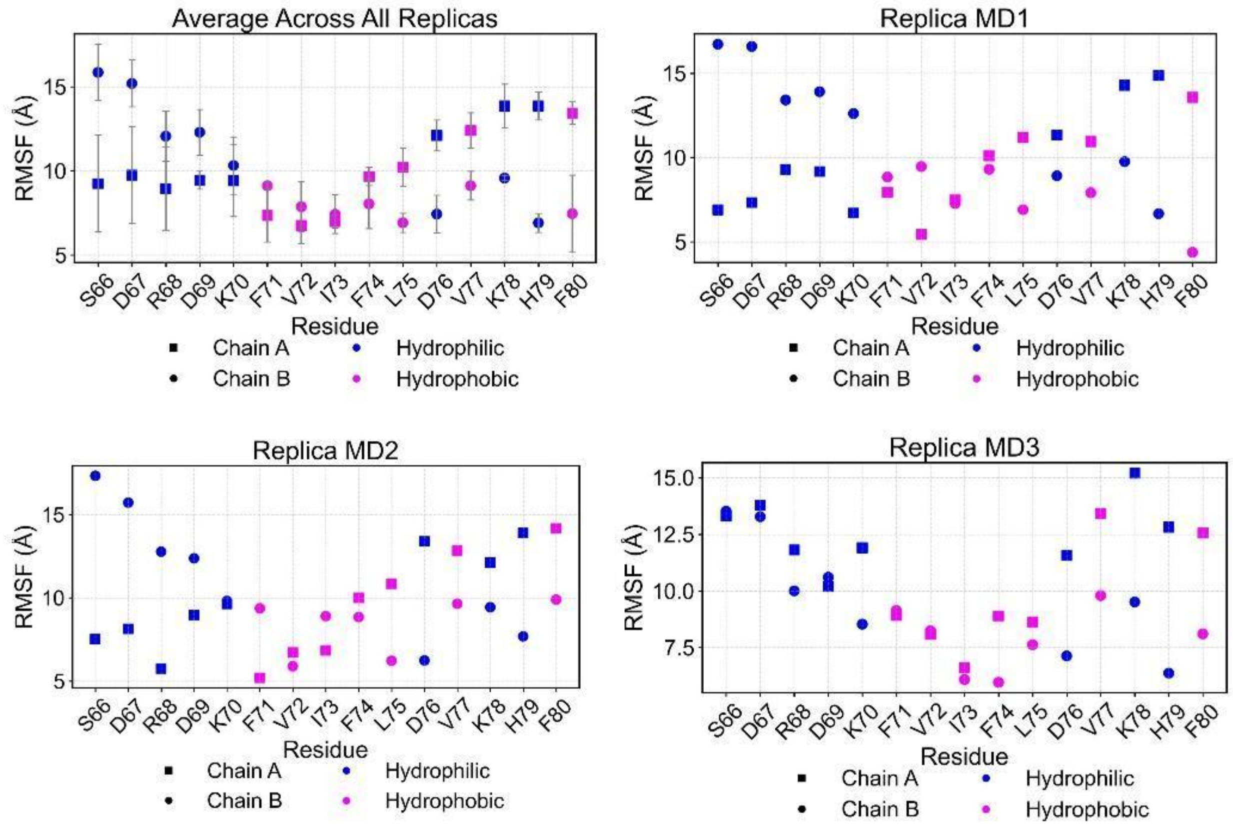

Figure S28. Root-Mean-Square-Fluctuation (**RMSF**) analysis of **αA66–80** crystallin peptide **dimer** formation. The plot shows average values across all replicas with corresponding standard deviations, followed by results from individual replicas.

X.II Trajectory analysis for αA66–80-Crystallin Peptide Tetramer formation

X.II.I Replica-wise RoG analysis for αA66–80-Crystallin Peptide tetramer formation

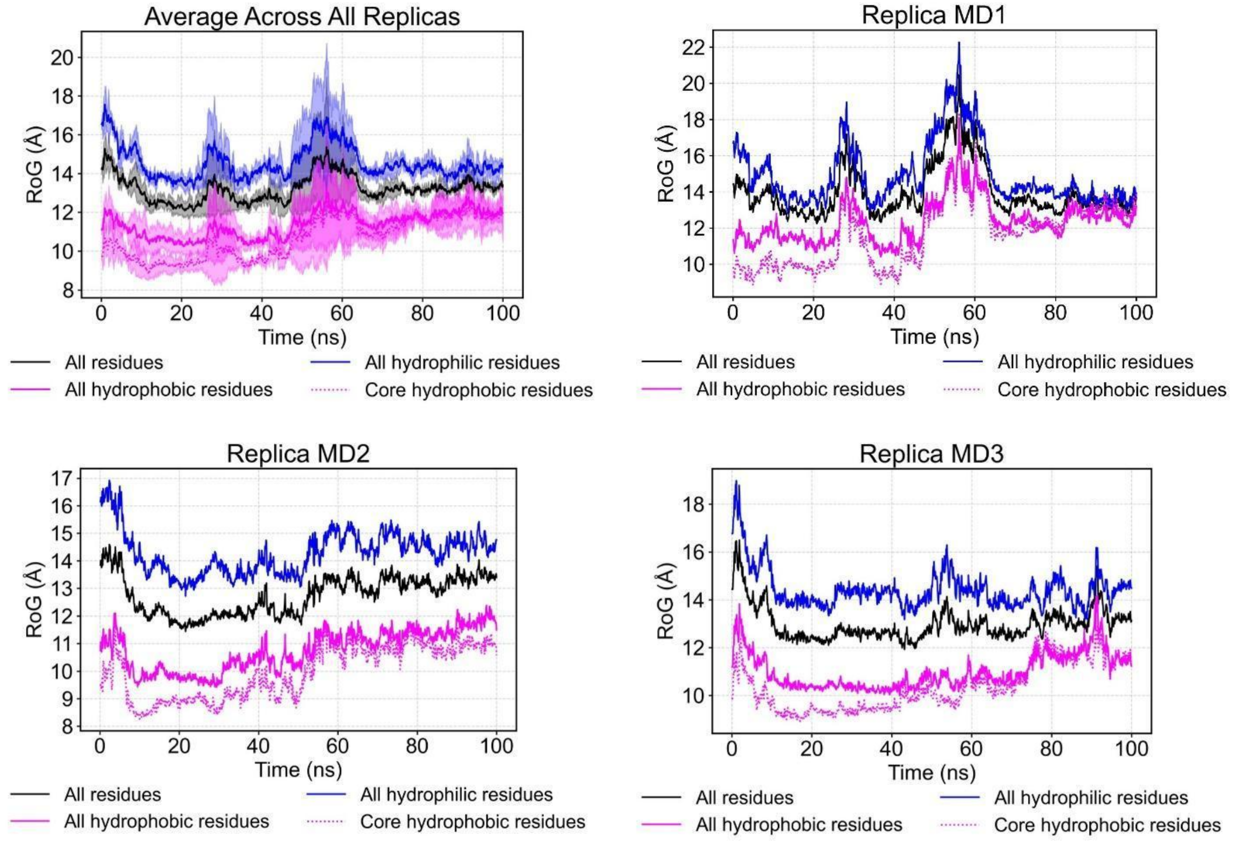

Figure S29. Radius of gyration (**RoG**) analysis of **αA66–80** crystallin peptide **tetramer** formation. The plot shows average values across all replicas with corresponding standard deviations, followed by results from individual replicas.

X.II.II Replica-wise SASA analysis for αA66–80-Crystallin Peptide tetramer formation

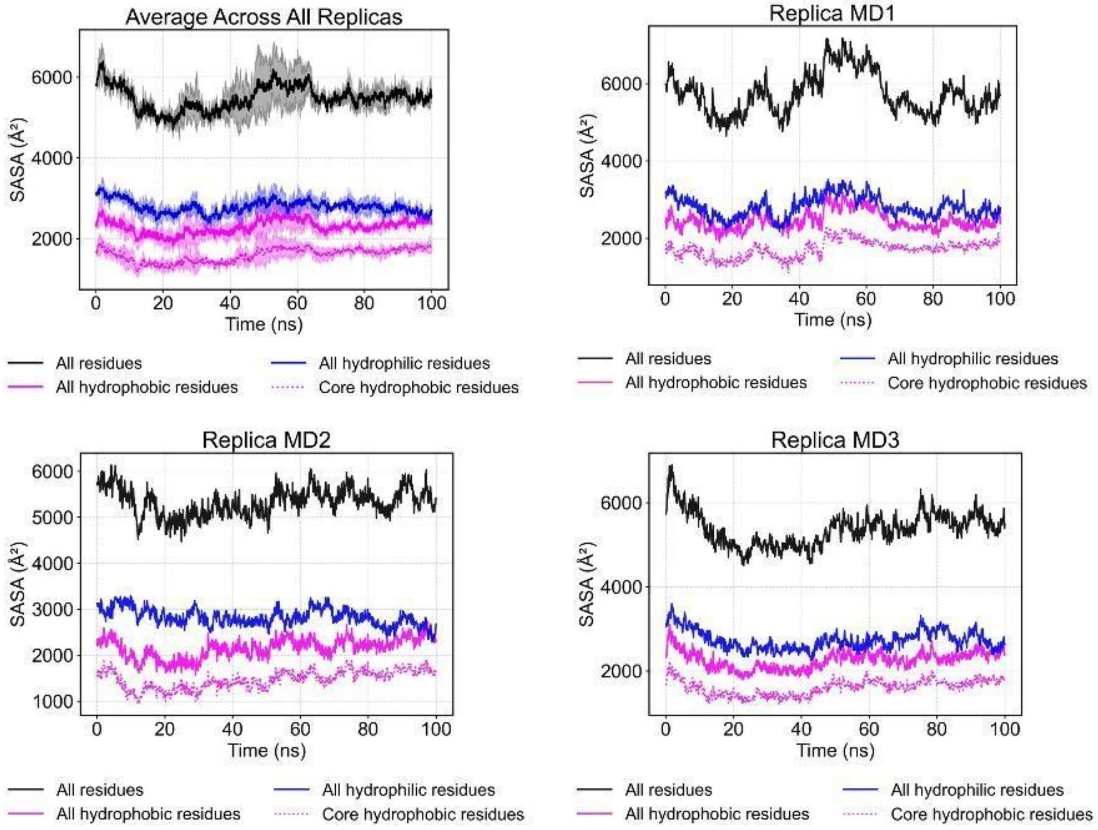

Figure S30. Solvent Accessible Surface Area (**SASA**) analysis of **αA66–80** crystallin peptide **tetramer** formation. The plot shows average values across all replicas with corresponding standard deviations, followed by results from individual replicas.

X.II.III Replica-wise RMSF analysis for αA66–80-Crystallin Peptide tetramer formation

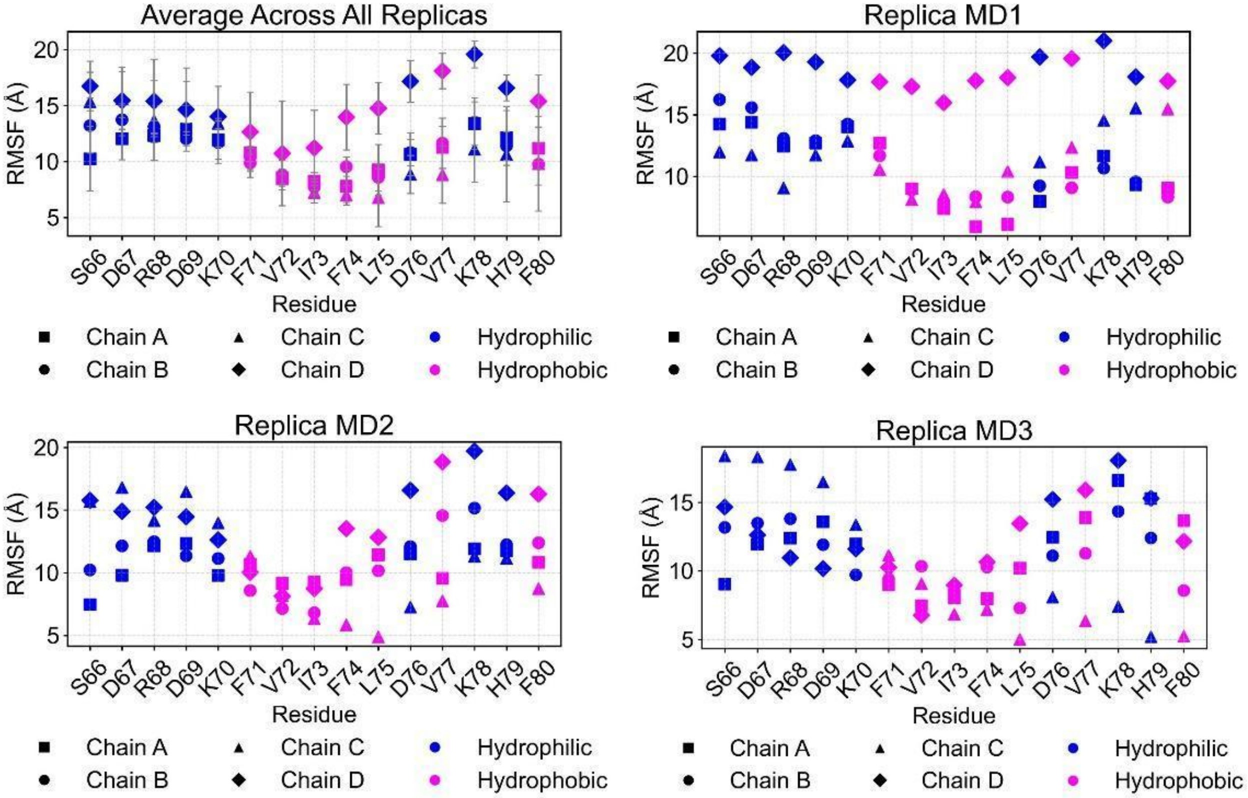

Figure S31. Root-Mean-Square-Fluctuation (**RMSF**) analysis of **αA66–80** crystallin peptide **tetramer** formation. The plot shows average values across all replicas with corresponding standard deviations, followed by results from individual replicas.

X.III Trajectory analysis for αA66–80-Crystallin Peptide dimer stability and equilibration

X.III.I Replica-wise RMSD analysis for αA66–80-Crystallin Peptide dimer stability and equilibration

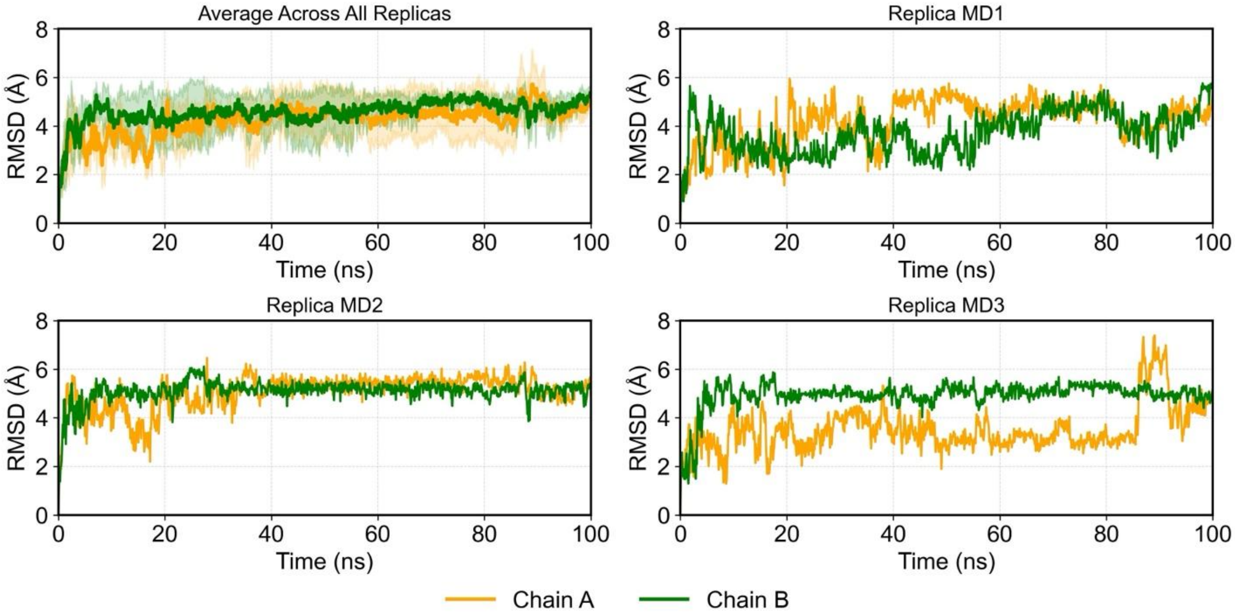

Figure S32. Root-mean-square deviation (**RMSD**) analysis relative to the first simulation frame for the **αA66–80** crystallin peptide **dimer**, illustrating structural stability and equilibration during the simulation. The figure presents the average RMSD profile across three independent replicas with corresponding standard deviations, followed by the individual replica trajectories.

X.III.II Replica-wise RoG analysis for αA66–80-Crystallin Peptide dimer stability and equilibration

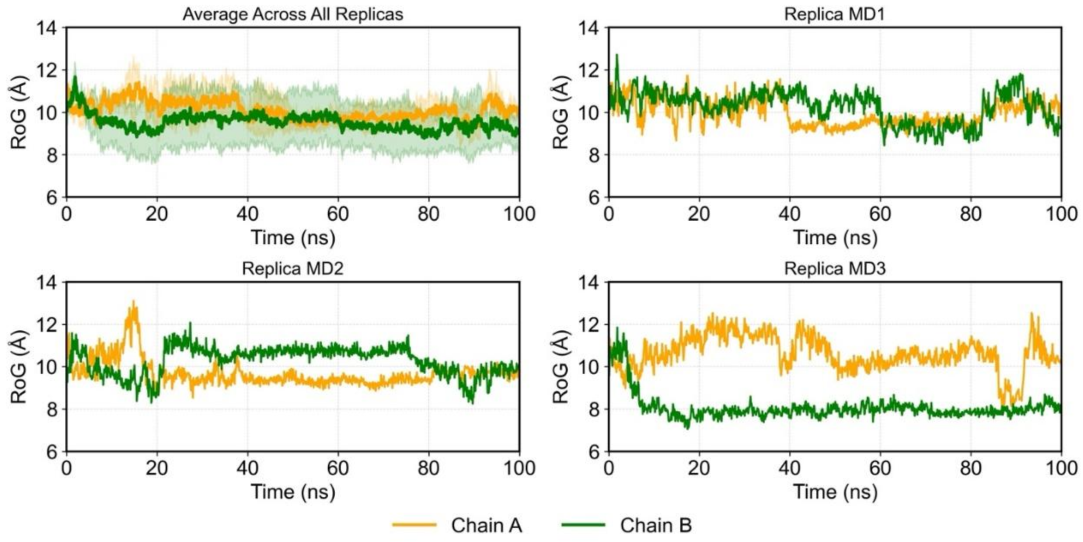

Figure S33. Radius of gyration (**RoG**) analysis of **αA66–80** crystallin peptide **dimer**, illustrating structural stability and equilibration during the simulation. The plot shows average values across all replicas with corresponding standard deviations, followed by results from individual replicas.

X.III.III Replica-wise SASA analysis for αA66–80-Crystallin Peptide dimer stability and equilibration

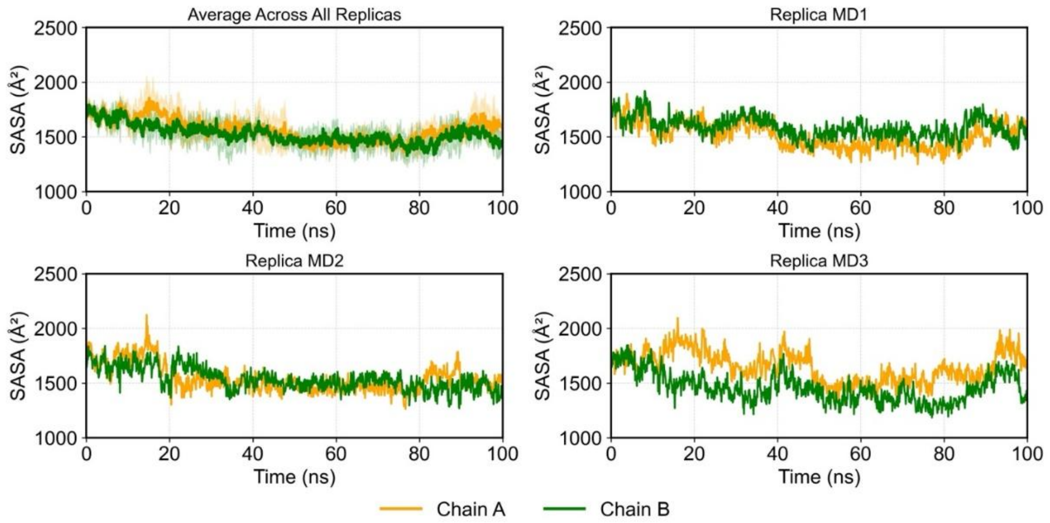

Figure S34. Solvent Accessible Surface Area (**SASA**) analysis of **αA66–80** crystallin peptide **dimer**, illustrating structural stability and equilibration during the simulation. The plot shows average values across all replicas with corresponding standard deviations, followed by results from individual replicas.

X.IV Trajectory analysis for αA66–80-Crystallin Peptide Tetramer stability and equilibration

X.IV.I Replica-wise RMSD analysis for αA66–80-Crystallin Peptide tetramer stability and equilibration

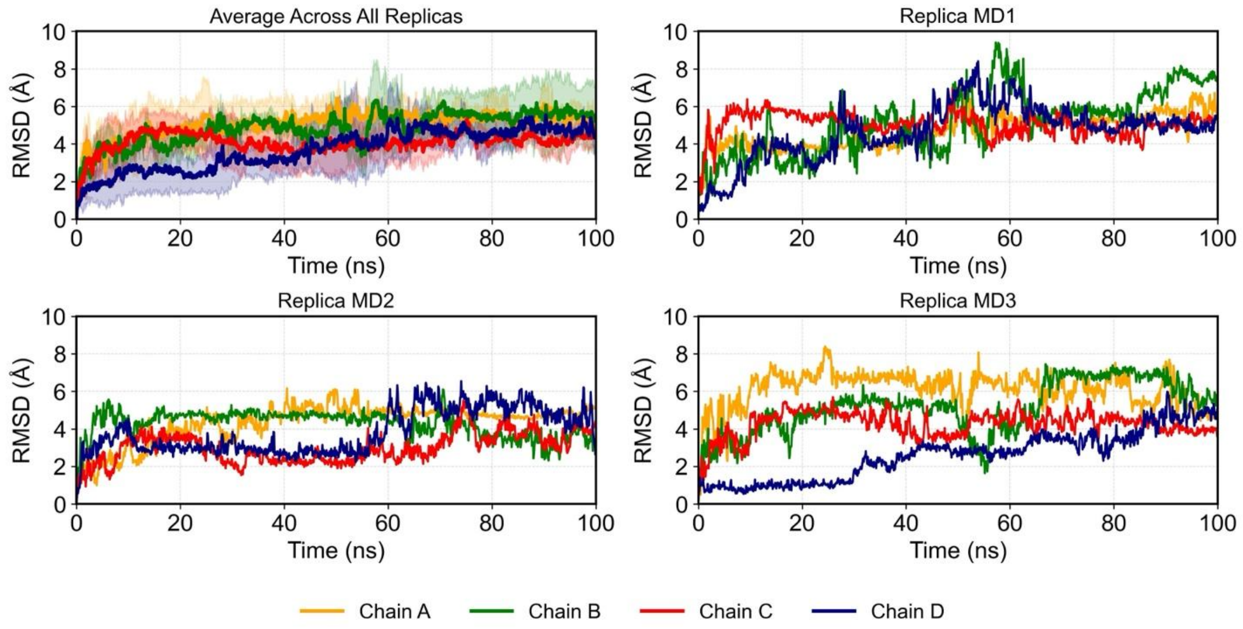

Figure S35. Root-mean-square deviation (**RMSD**) analysis relative to the first simulation frame for the **αA66–80** crystallin peptide **teramer**, illustrating structural stability and equilibration during the simulation. The figure presents the average RMSD profile across three independent replicas with corresponding standard deviations, followed by the individual replica trajectories.

X.IV.II Replica-wise RoG analysis for αA66–80-Crystallin Peptide tetramer stability and equilibration

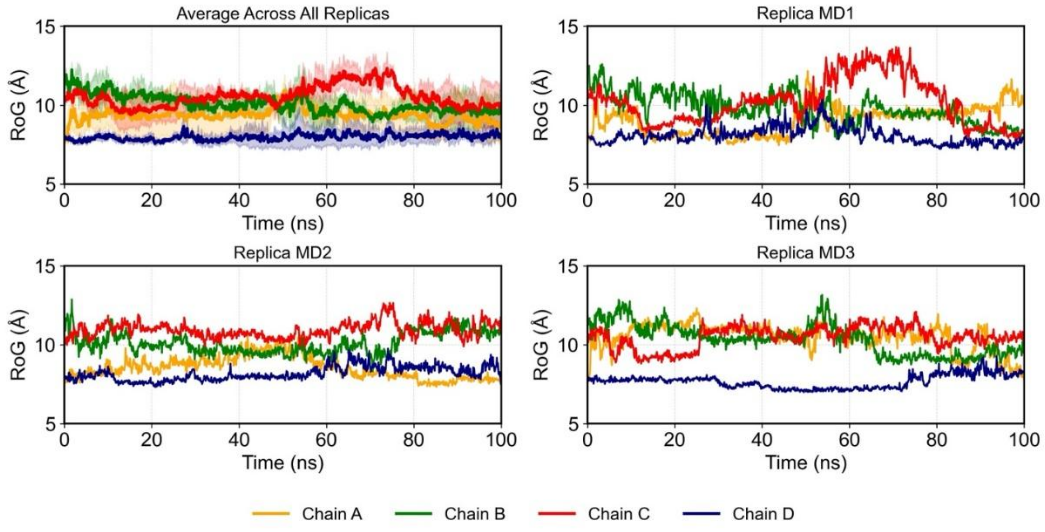

Figure S36. Radius of gyration (**RoG**) analysis of **αA66–80** crystallin peptide **tetramer**, illustrating structural stability and equilibration during the simulation. The plot shows average values across all replicas with corresponding standard deviations, followed by results from individual replicas.

X.IV.II Replica-wise SASA analysis for αA66–80-Crystallin Peptide tetramer stability and equilibration

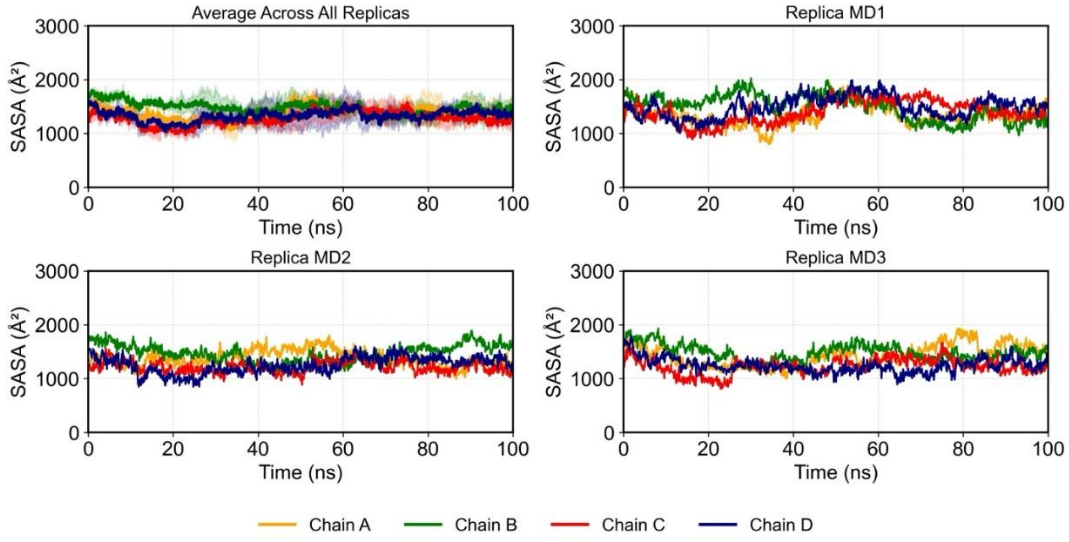

Figure S37. Solvent Accessible Surface Area (**SASA**) analysis of **αA66–80** crystallin peptide **tetramer**, illustrating structural stability and equilibration during the simulation. The plot shows average values across all replicas with corresponding standard deviations, followed by results from individual replicas.

X.III. Linear Interaction energy (lie) analysis for αA66–80-Crystallin Aggregation Mechanism

To investigate the interaction behaviours, linear interaction energy (lie) analysis was performed for both the dimer and tetramer systems, as illustrated in Figure S32. Each plot represents averages over three replicas, with individual replica data provided in Figures S33-S36. The lie approach quantifies the residue-residue interaction energy throughout the simulation trajectory and decomposes it into electrostatic (Eele) and van der Waals (EvdW) contributions.

To gain residue-specific insights into dimer formation, the interaction analysis was categorized into six groups: (a) all residues of chain A interacting with all residues of chain B (black); (b) all hydrophilic residues of chain A interacting with all hydrophilic residues of chain B (blue); (c) all hydrophobic residues of chain A interacting with all hydrophobic residues of chain B (magenta); (d) core-hydrophobic residues of chain A interacting with core-hydrophobic residues of chain B (dotted magenta); (e) all hydrophobic residues of chain A interacting with all hydrophilic residues of chain B (green); and (f) core-hydrophobic residues of chain A interacting with all hydrophilic residues of chain B (dotted green) for the dimer.

For the tetramer analysis, chain B was treated as the reference interaction partner, while residues from chains A, C, and D were clustered together for comparison.

As shown in Figure S32, in the dimer system, the electrostatic interaction energy is predominantly governed by interactions between the hydrophilic residues of chains A and B. This is evidenced by the close overlap between the total electrostatic energy (Eele, black) and the hydrophilic–hydrophilic Eele contribution (blue).

A similar trend is observed in the tetramer system, where electrostatic stabilization is mainly driven by interactions between the hydrophilic residues of chain B and those of chains A, C, and D. Here again, the near-complete overlap of the total Eele (black) with the hydrophilic–hydrophilic Eele component (blue) confirms the dominant contribution of hydrophilic residue interactions to the overall electrostatic energy.

However, hydrophobic residues show substantial contribution to the van der Waals component, as reflected in the EvdW profiles of the dimer system. This contribution is more pronounced in the tetramer, where hydrophobic–hydrophobic interactions (magenta) constitute a major component

of the total EvdW energy highlighting their pivotal role in mediating van der Waals stabilization and driving aggregation. Together with the lie, RoG, SASA, and RMSF analysis, these results indicate that the core hydrophobic residues remain central to aggregation and may serve as promising targets for its inhibition.

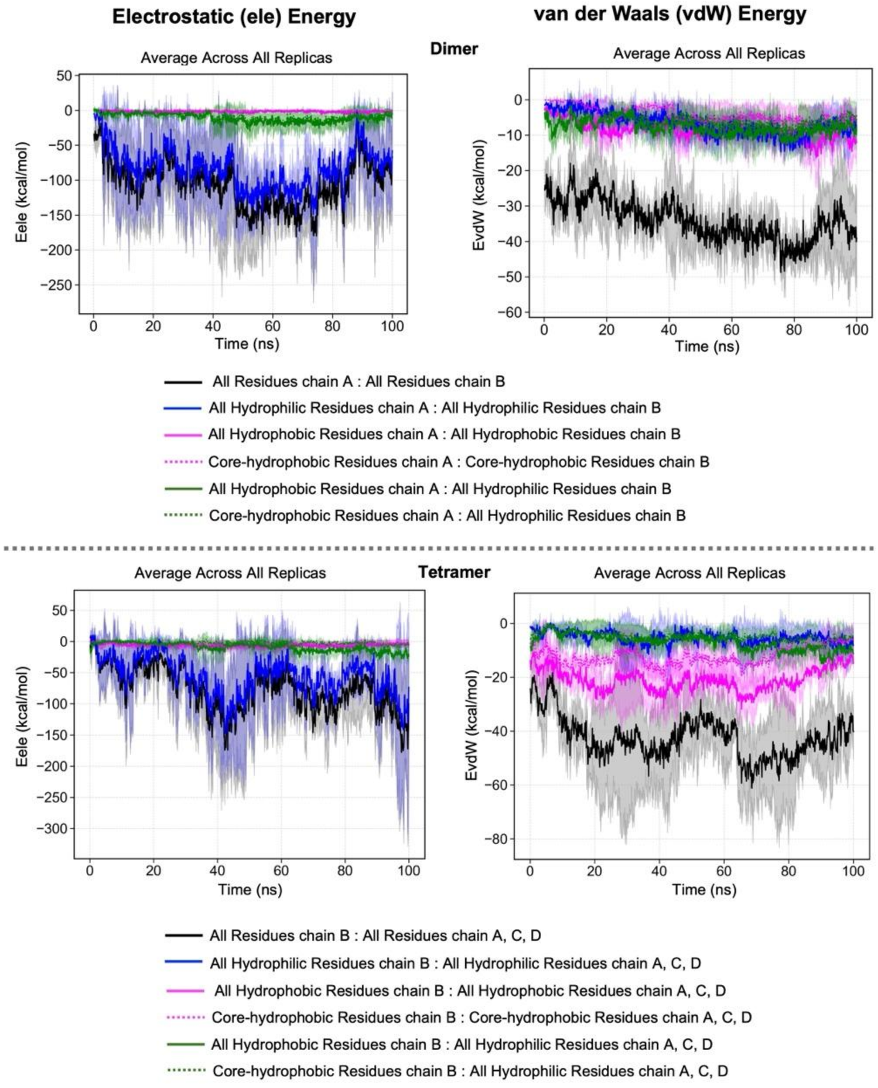

Figure S38. The linear interaction energy (**lie**) analysis of the **αA66–80** crystallin peptide during **dimer** and **tetramer** formation. The plots illustrate the **electrostatic (Eele)** and **van der Waals (EvdW)** interaction components over the simulation trajectory. For the tetramer, chain B was treated as the reference chain, and its interactions were analysed with residues from chains A, C, and D grouped together. The results highlight dominant hydrophilic contributions to electrostatic

interactions and pronounced hydrophobic contributions to van der Waals stabilization, particularly in the tetramer.

X.III.I Replica-wise lie(electrostatic) analysis for αA66–80-Crystallin dimer formation

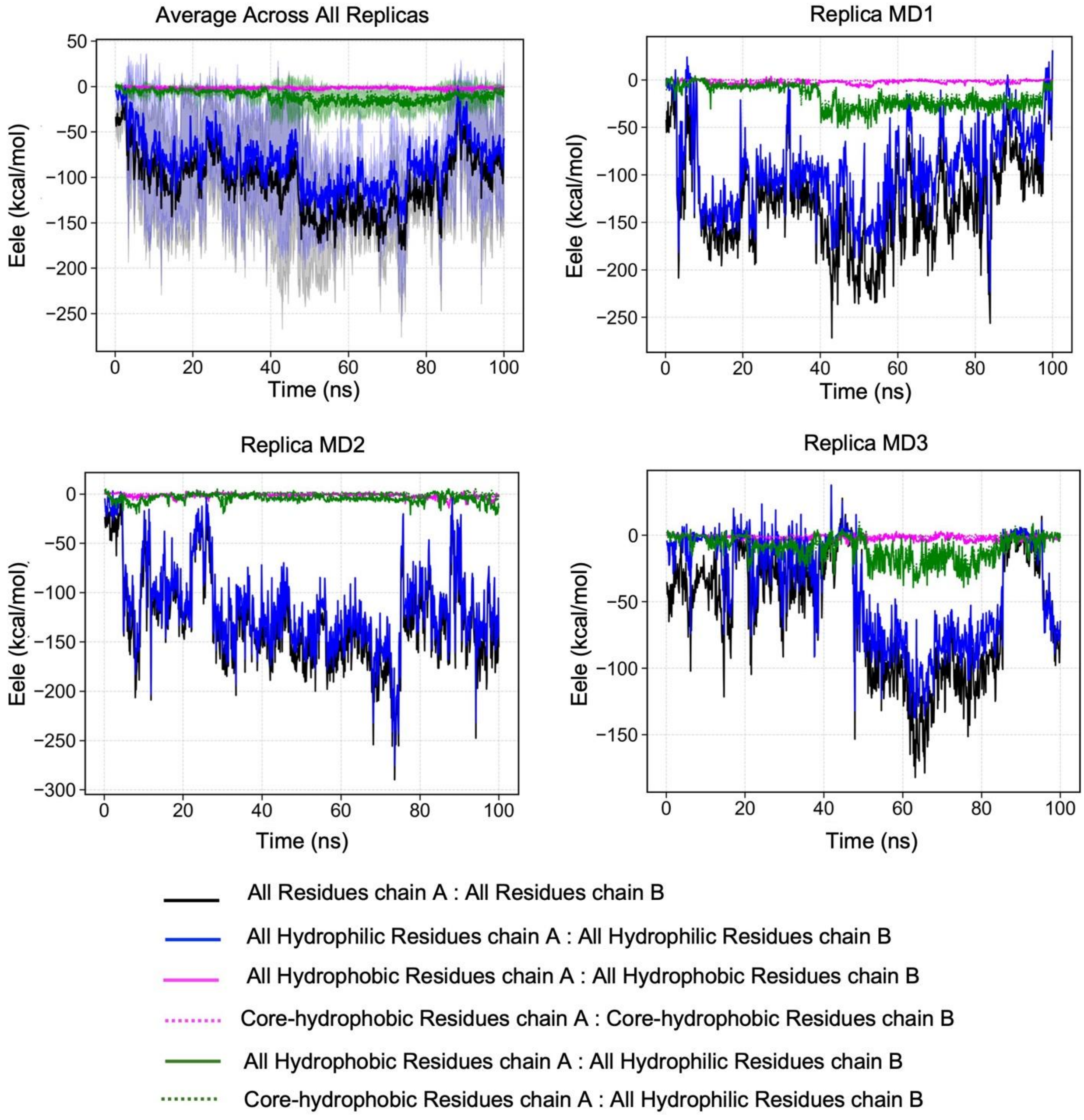

Figure S39. The linear interaction energy (**lie)** analysis (**electrostatics**) of the **αA66–80** crystallin peptide during **dimer** formation. The plot shows average values across all replicas with corresponding standard deviations, followed by results from individual replicas.

X.III.II Replica-wise lie(van der Waals) analysis for αA66–80-Crystallin dimer formation

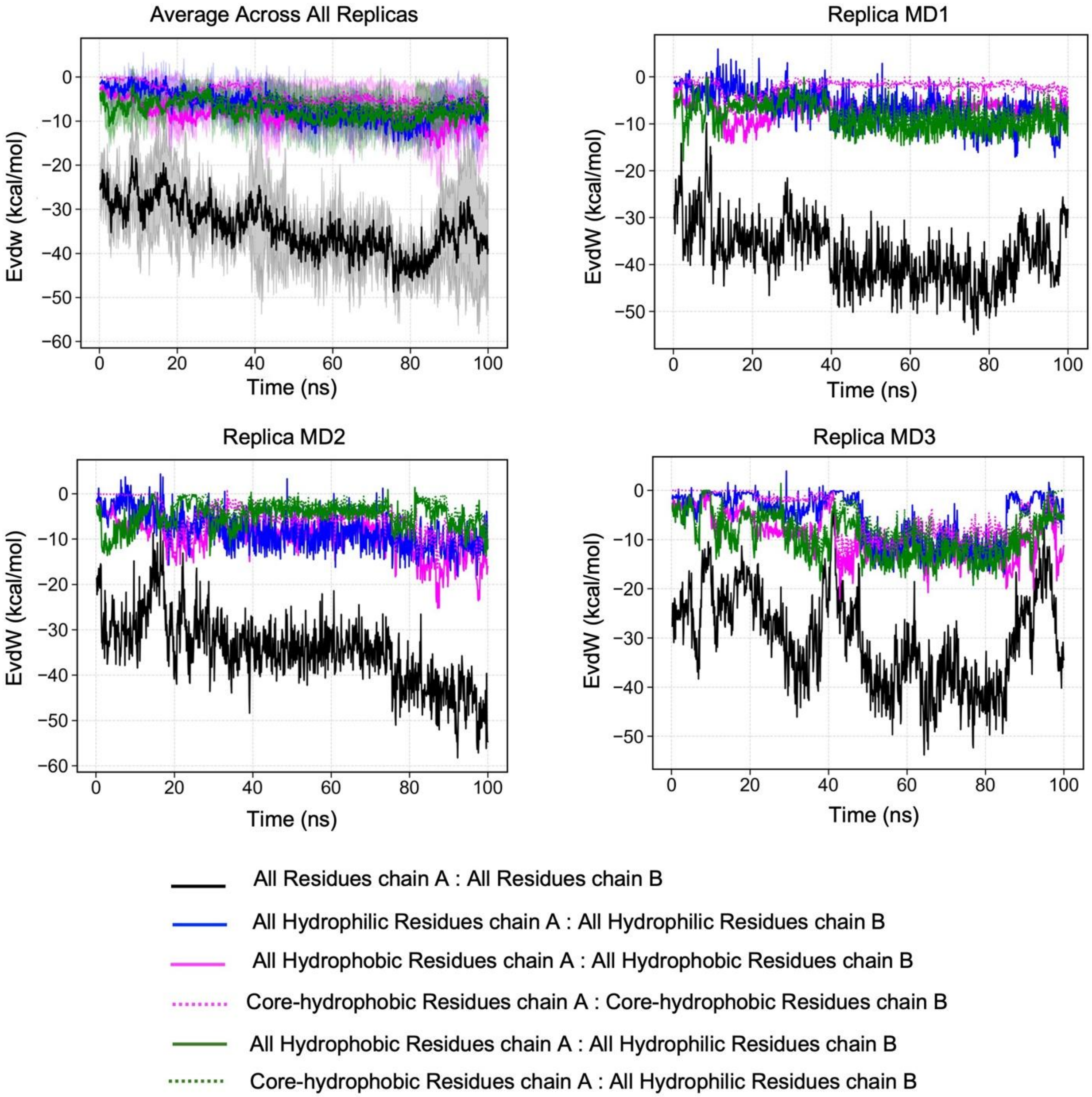

Figure S40. The linear interaction energy (**lie**) analysis (**van der Waals**) of the **αA66–80** crystallin peptide during **dimer** formation. The plot shows average values across all replicas with corresponding standard deviations, followed by results from individual replicas.

X.II.IV Replica-wise lie(electrostatic) analysis for αA66–80-Crystallin tetramer formation

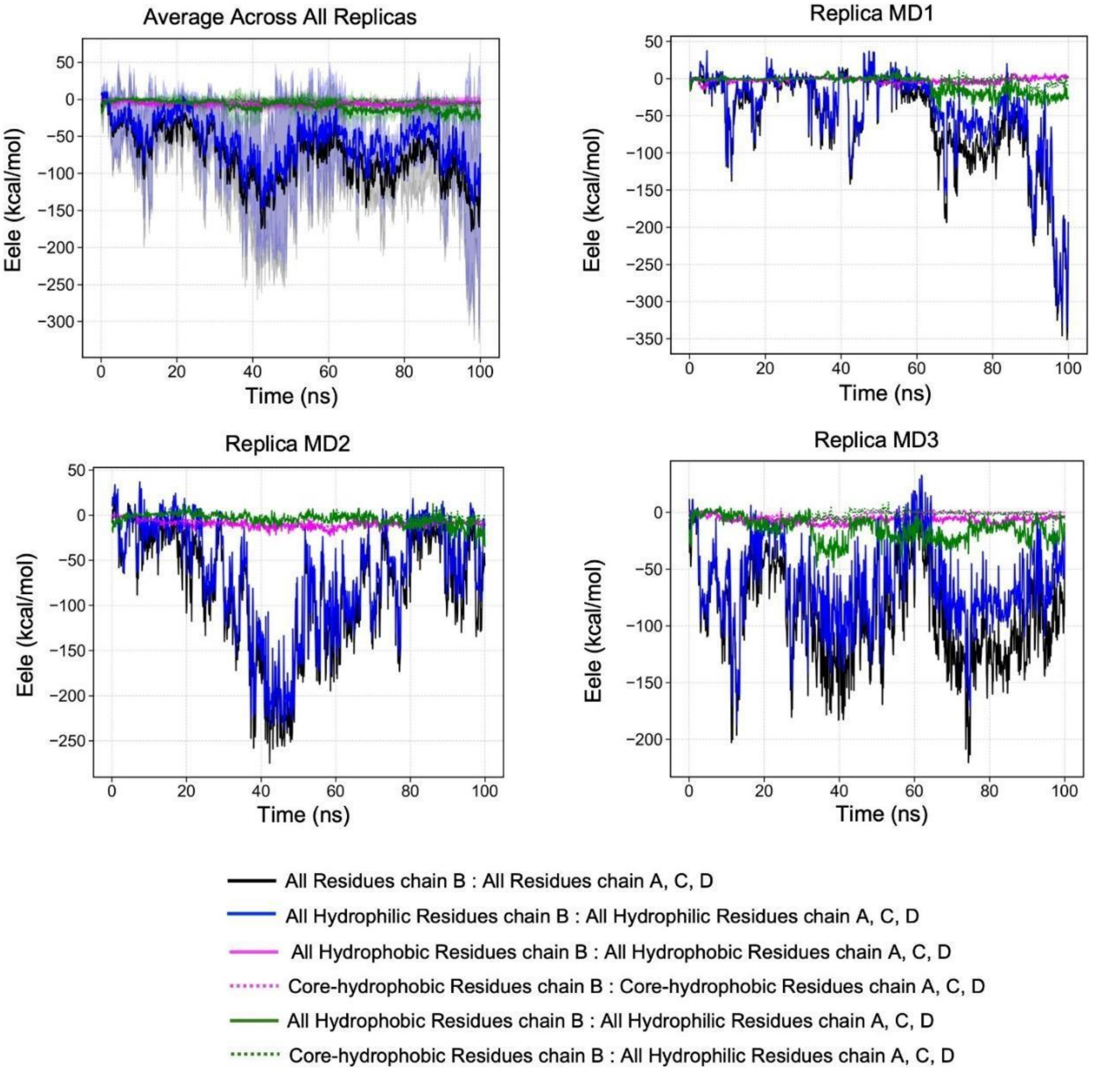

Figure S41. The linear interaction energy (**lie**) analysis (**electrostatics**) of the **αA66–80** crystallin peptide during **tetramer** formation. The plot shows average values across all replicas with corresponding standard deviations, followed by results from individual replicas.

X.II.V Replica-wise lie(van der Waals) analysis for αA66–80-Crystallin tetramer formation

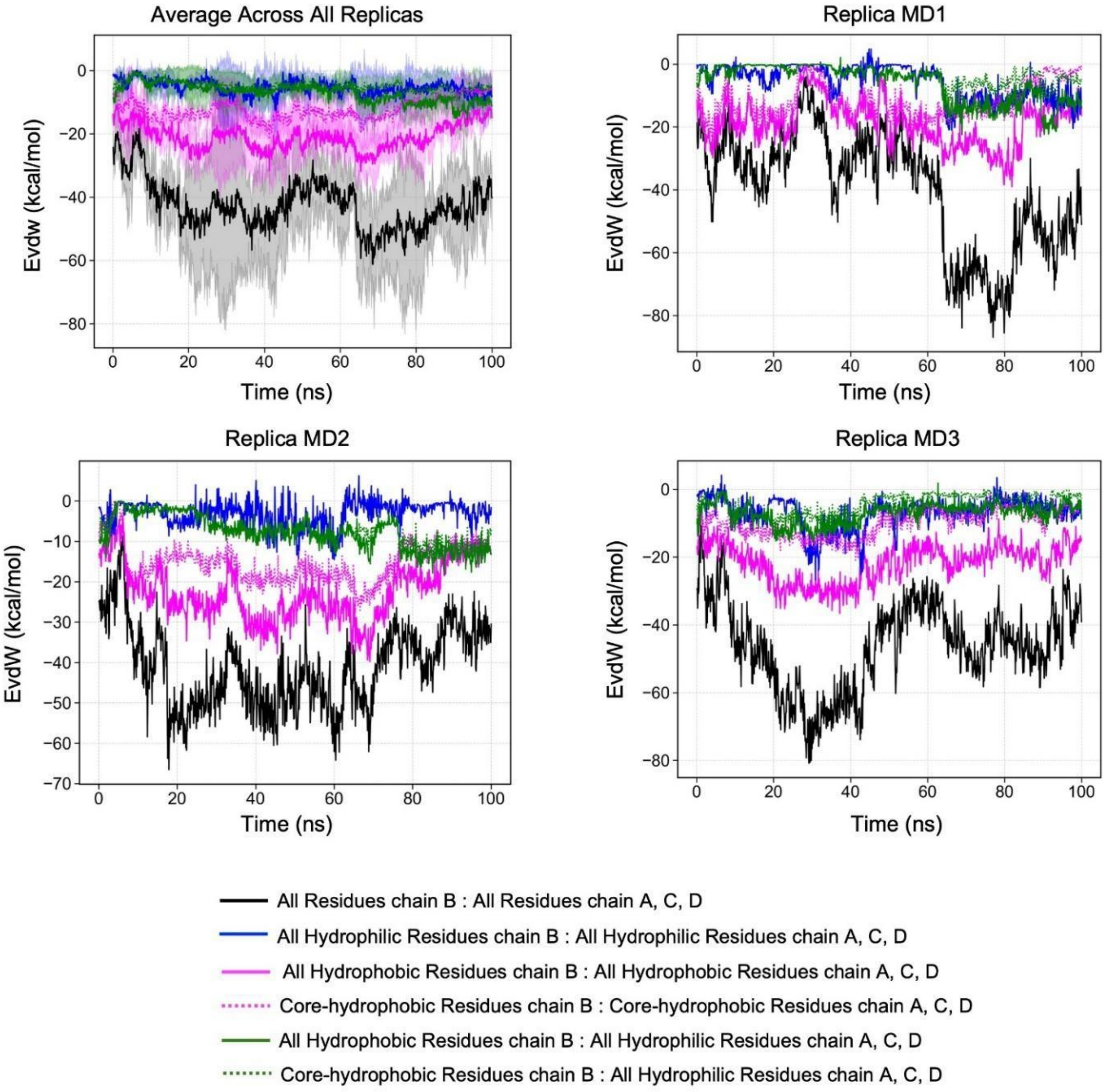

Figure S42. The linear interaction energy (**lie**) analysis (**van der Waals**) of the **αA66–80** crystallin peptide during **tetramer** formation. The plot shows average values across all replicas with corresponding standard deviations, followed by results from individual replicas.

XI. Molecular Docking of αA66–80 Peptide–Resorcinarene Complexes

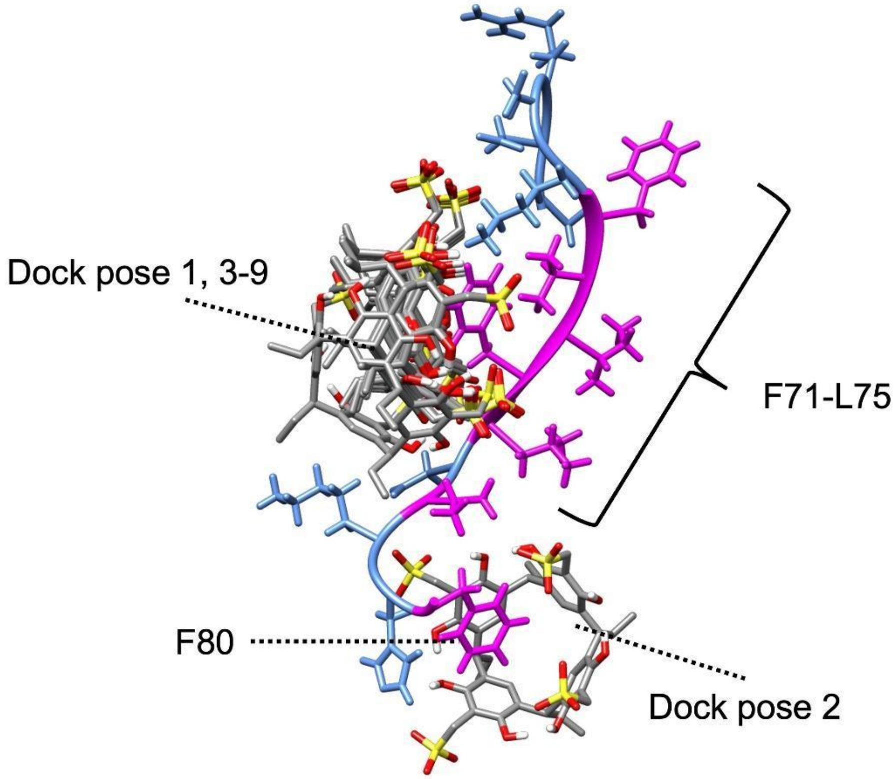

Dock Pose	Binding Affinity/ kcal mol^{-1}
Dock 1	-5.1903
Dock 2	-5.1743
Dock 3	-5.1630
Dock 4	-5.1469
Dock 5	-5.0993
Dock 6	-5.0767
Dock 7	-5.0553
Dock 8	-5.0456
Dock 9	-5.0399

Figure S43. Docking results of **UR-4S** with the **αA66–80** peptide. The best-ranked pose localized near residue F80, whereas other poses predominantly occupied the core hydrophobic region (F71–L75). Dock pose 2 was selected for subsequent MD simulations to ensure consistency with the other tested resorcinarenes.

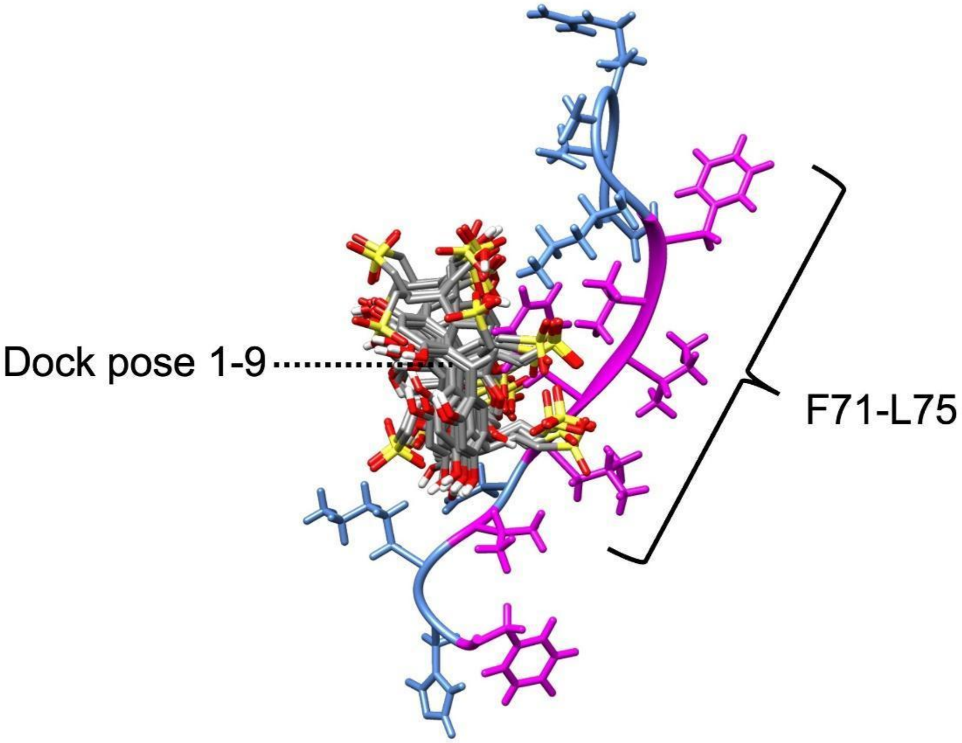

Dock Pose	Binding Affinity/kcal mol^{-1}
Dock 1	-5.3257
Dock 2	-5.1663
Dock 3	-5.1243
Dock 4	-5.1226
Dock 5	-5.0780
Dock 6	-5.0615
Dock 7	-5.0350
Dock 8	-4.9883
Dock 9	-4.9855

Figure S44. Docking results of **LR-4S** with the **αA66–80** peptide. All top-ranked poses were located at the core hydrophobic region (F71–L75), and dock pose 1 was selected for further analysis.

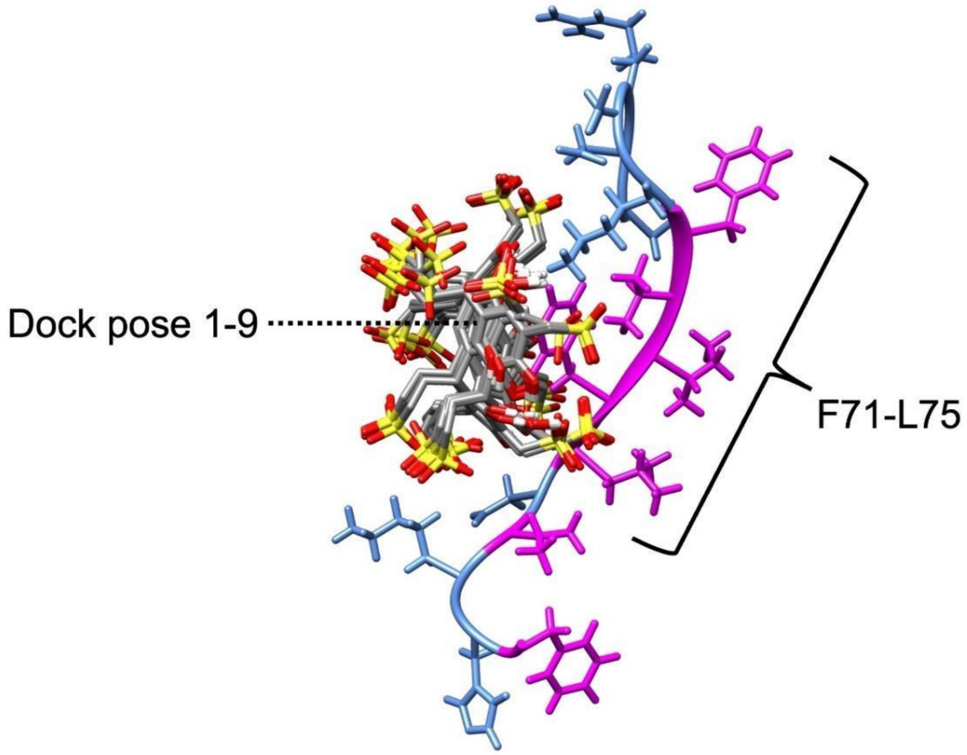

Dock Pose	Binding Affinity/kcal mol^{-1}
Dock 1	-4.7731
Dock 2	-4.7528
Dock 3	-4.6520
Dock 4	-4.6218
Dock 5	-4.5786
Dock 6	-4.5748
Dock 7	-4.5466
Dock 8	-4.5451
Dock 9	-4.5197

Figure S45. Docking results of **MR-8S** with the **αA66–80** peptide. All top-ranked poses were located at the core hydrophobic region (F71–L75), and dock pose 1 was selected for further analysis.

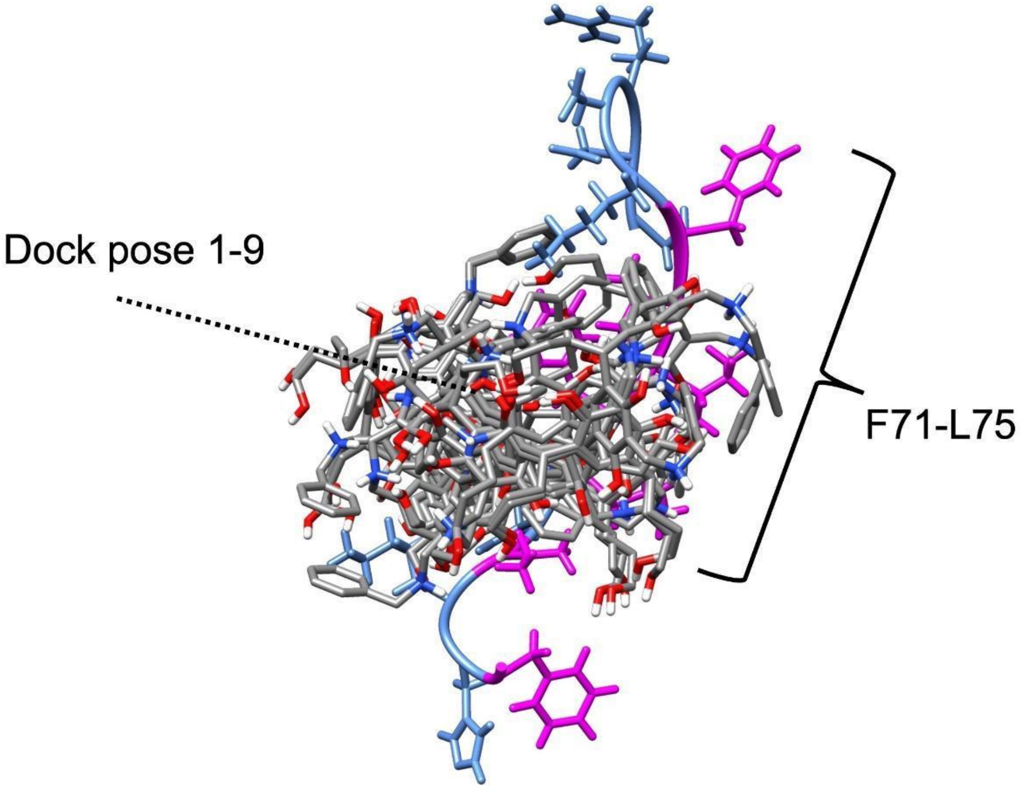

Dock Pose	Binding Affinity/ kcal mol^{-1}
Dock 1	-3.7622
Dock 2	-3.7085
Dock 3	-3.6853
Dock 4	-3.6672
Dock 5	-3.6550
Dock 6	-3.6216
Dock 7	-3.6208
Dock 8	-3.6147
Dock 9	-3.5852

Figure S46. Docking results of **UR-4A** with the **αA66–80** peptide. All top-ranked poses were located at the core hydrophobic region (F71–L75), and dock pose 1 was selected for further analysis.

XII. Investigation of Resorcinarene-Mediated Inhibition of αA66–80 Peptide Aggregation

XII.I Replica-wise RMSF analysis for αA66–80-Crystallin Peptide and Resorcinarene Complex

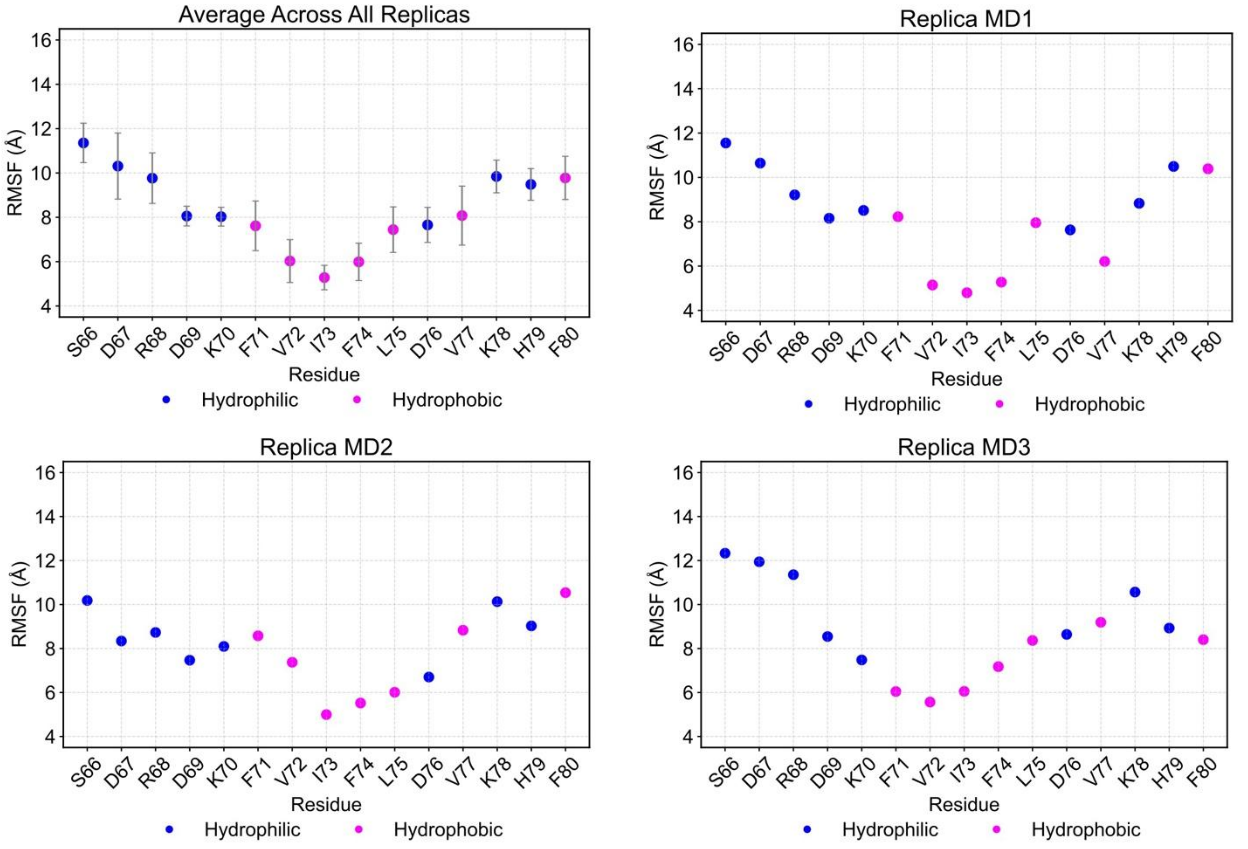

Figure S47. Root-Mean-Square-Fluctuation (**RMSF**) analysis of **αA66–80 peptide monomer**. The plot shows average values across all replicas with corresponding standard deviations, followed by results from individual replicas.

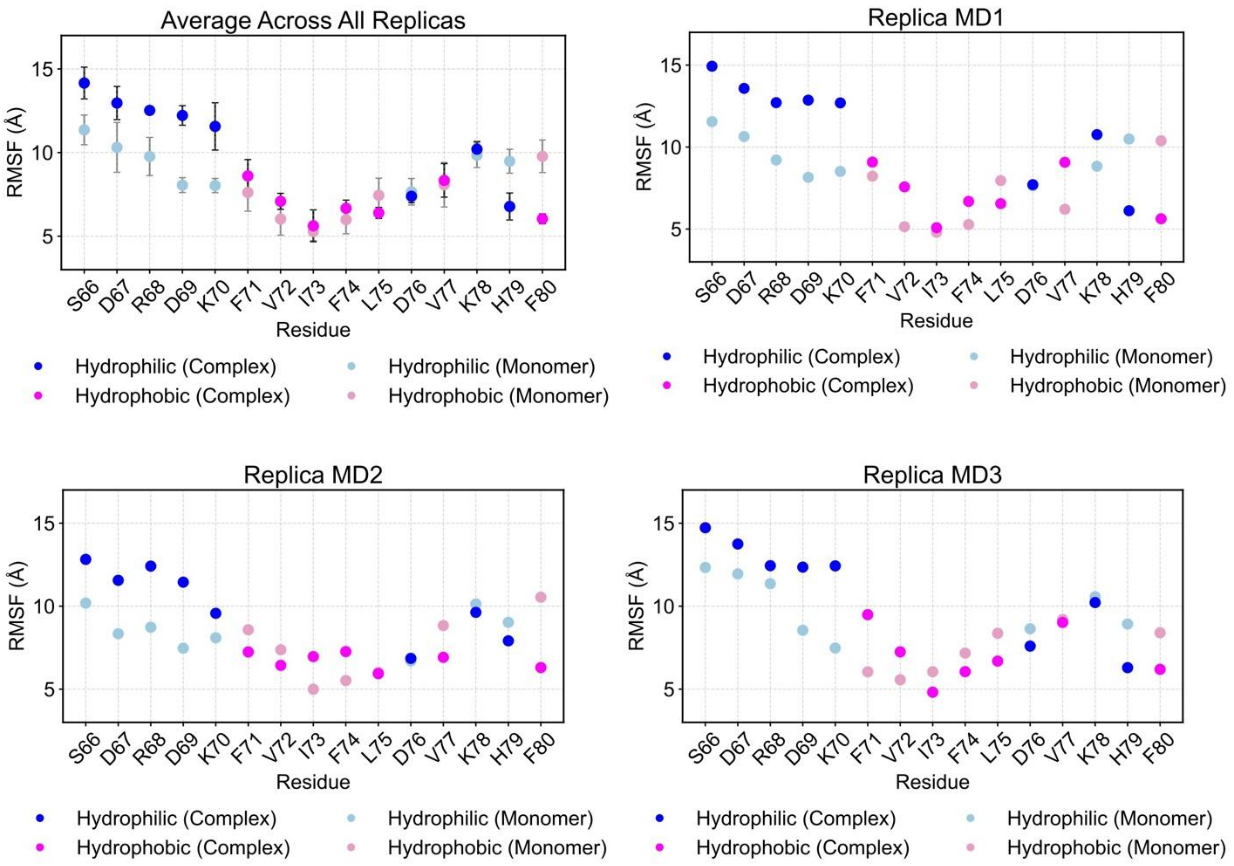

Figure S48. Root-Mean-Square-Fluctuation (**RMSF**) analysis of **αA66–80 peptide** interacting with **UR-4S**. The RMSF profile of the αA66–80 peptide monomer is included as a faint reference for direct comparison. The plot shows average values across all replicas with corresponding standard deviations, followed by results from individual replicas.

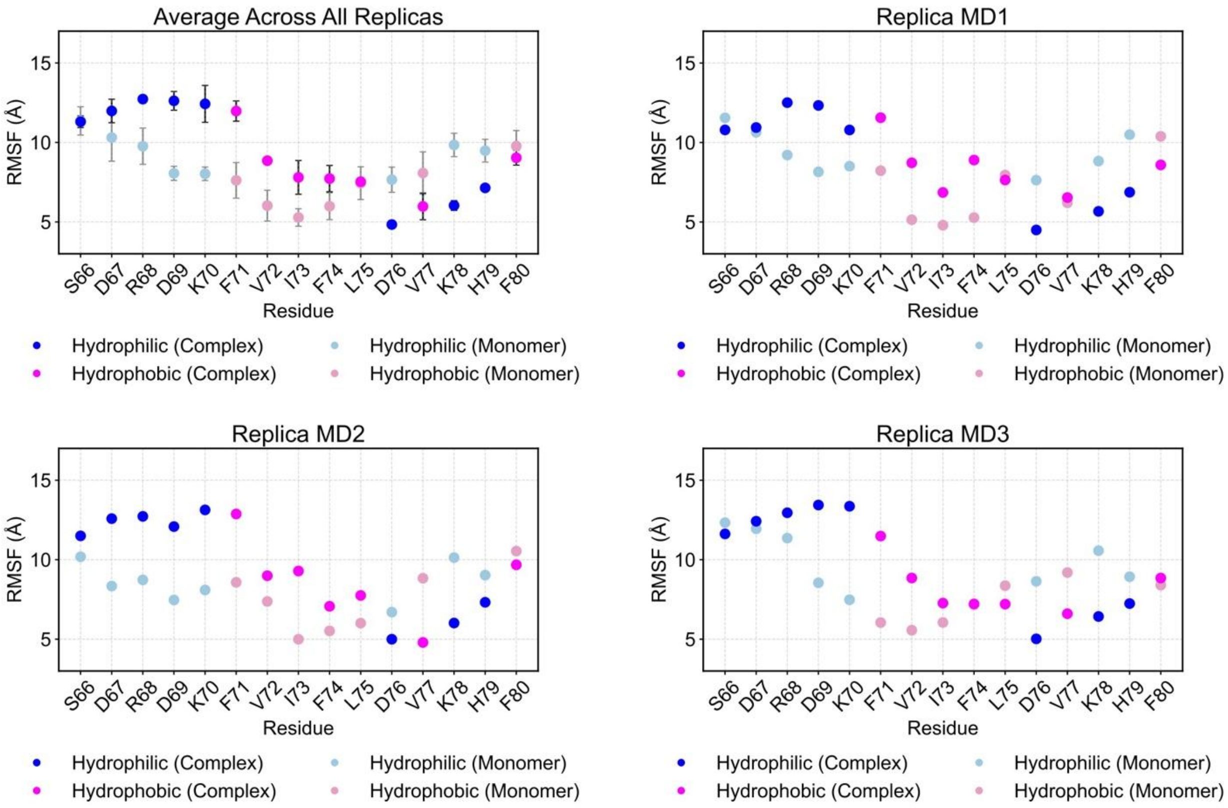

Figure S49. Root-Mean-Square-Fluctuation (**RMSF**) analysis of **αA66–80 peptide** interacting with **LR-4S**. The RMSF profile of the αA66–80 peptide monomer is included as a faint reference for direct comparison. The plot shows average values across all replicas with corresponding standard deviations, followed by results from individual replicas.

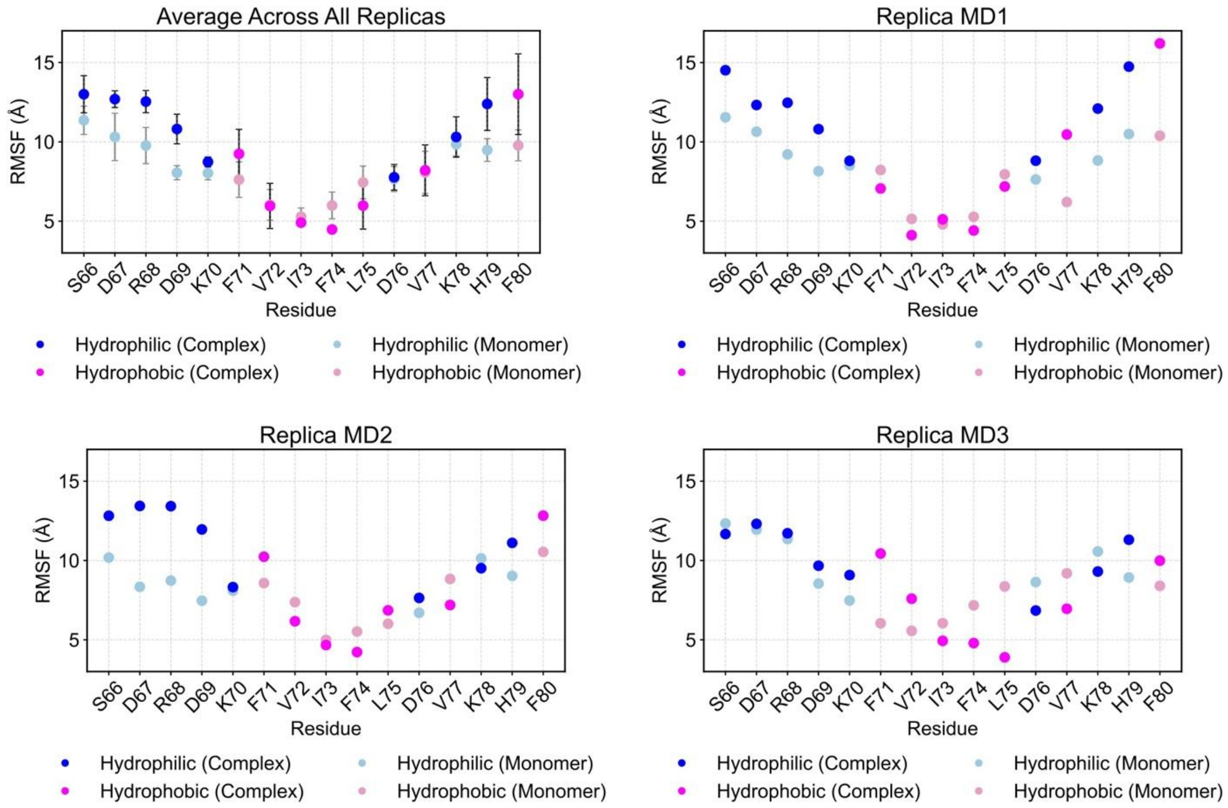

Figure S50. Root-Mean-Square-Fluctuation (**RMSF**) analysis of **αA66–80 peptide** interacting with **MR-8S**. The RMSF profile of the αA66–80 peptide monomer is included as a faint reference for direct comparison. The plot shows average values across all replicas with corresponding standard deviations, followed by results from individual replicas.

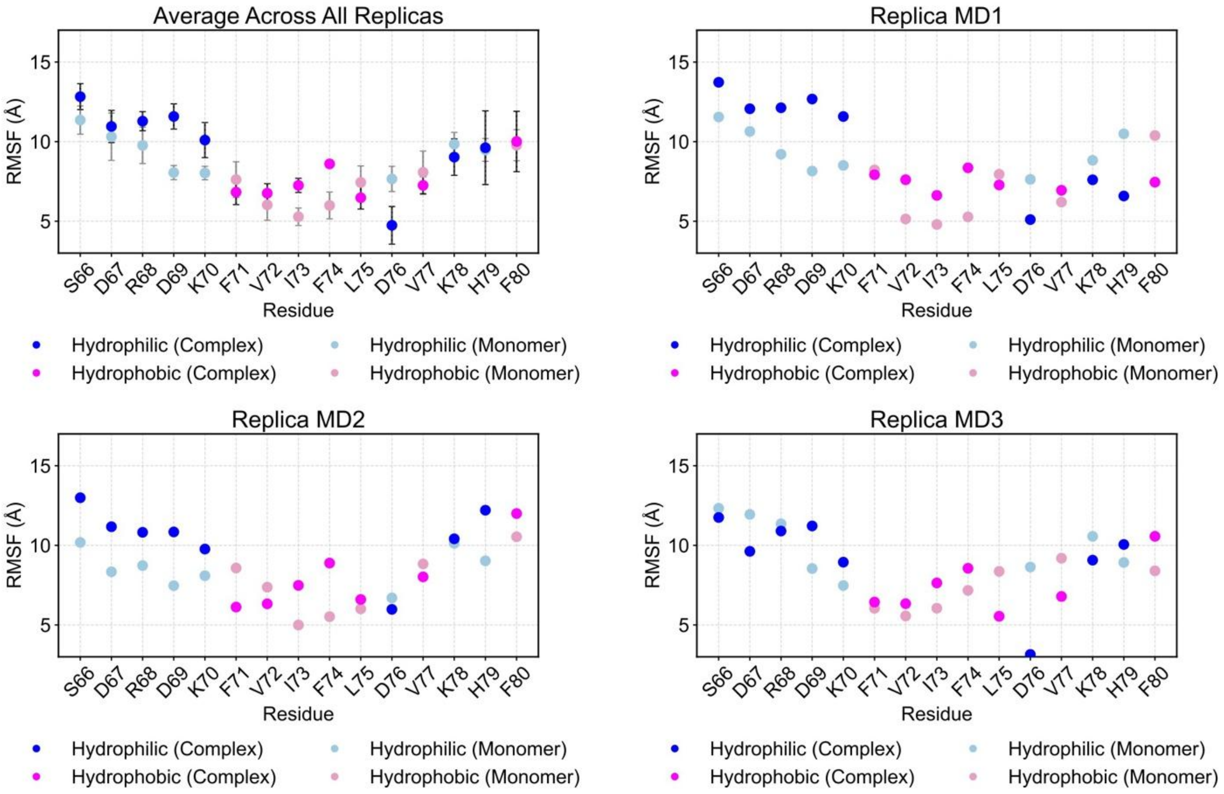

Figure S51. Root-Mean-Square-Fluctuation (**RMSF**) analysis of **αA66–80 peptide** interacting with **UR-4A**. The RMSF profile of the αA66–80 peptide monomer is included as a faint reference for direct comparison. The plot shows average values across all replicas with corresponding standard deviations, followed by results from individual replicas.

XII.II Replica-wise SASA analysis for αA66–80-Crystallin Peptide and Resorcinarene Complex

Table S3. Quantitative assessment of hydrophobic core shielding based on the solvent-accessible surface area (SASA) of residues F71–L75 of the αA66–80 peptide monomer and in complexes with different resorcinarenes. SASA values are reported for each individual MD replica as well as averaged over **three independent replicas** and expressed as mean ± standard deviation ($Å^2$).

Replica	Monomer	UR-4S	LR-4S	UR-4A	MR-8S
**Replica 1**	593.71 ± 49.73	563.67 ± 48.65	615.24 ± 41.74	557.39 ± 49.03	480.50 ± 39.08
**Replica 2**	604.99 ± 43.80	533.43 ± 55.80	663.81 ± 50.32	492.05 ± 48.79	475.93 ± 45.24
**Replica 3**	636.48 ± 47.91	566.57 ± 45.23	603.98 ± 43.70	504.04 ± 46.72	426.64 ± 54.70
**Average**	611.73 ± 18.10	554.56 ± 14.99	627.68 ± 25.96	517.83 ± 28.40	461.02 ± 24.38

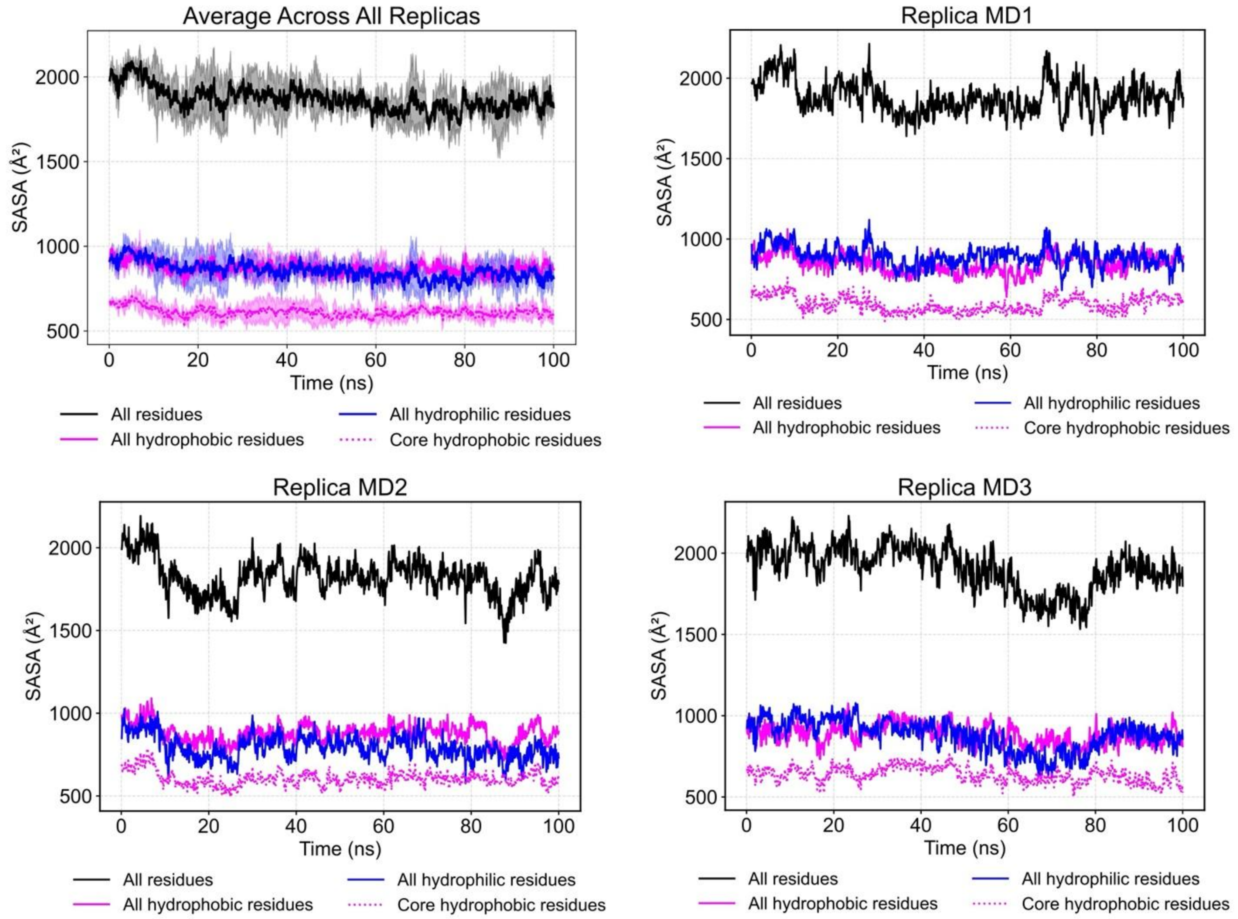

Figure S52. Solvent Accessible Surface Area (**SASA**) analysis of **αA66–80 peptide monomer**. The plot shows average values across all replicas with corresponding standard deviations, followed by results from individual replicas.

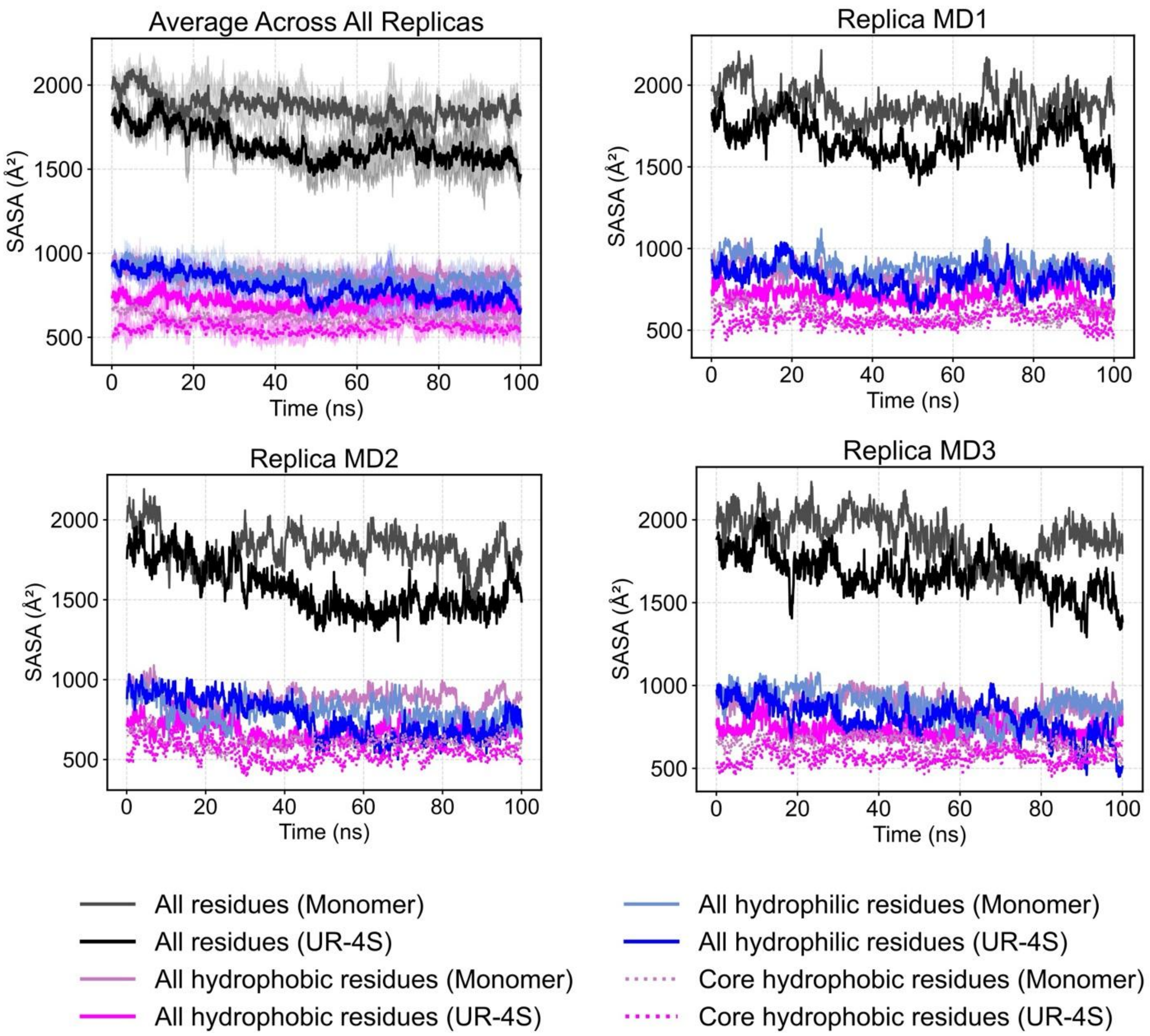

Figure S53. Solvent Accessible Surface Area (**SASA**) analysis of **αA66–80 peptide** interacting with **UR-4S**. The SASA profile of the αA66–80 peptide monomer is included as a faint reference for direct comparison. The plot shows average values across all replicas with corresponding standard deviations, followed by results from individual replicas.

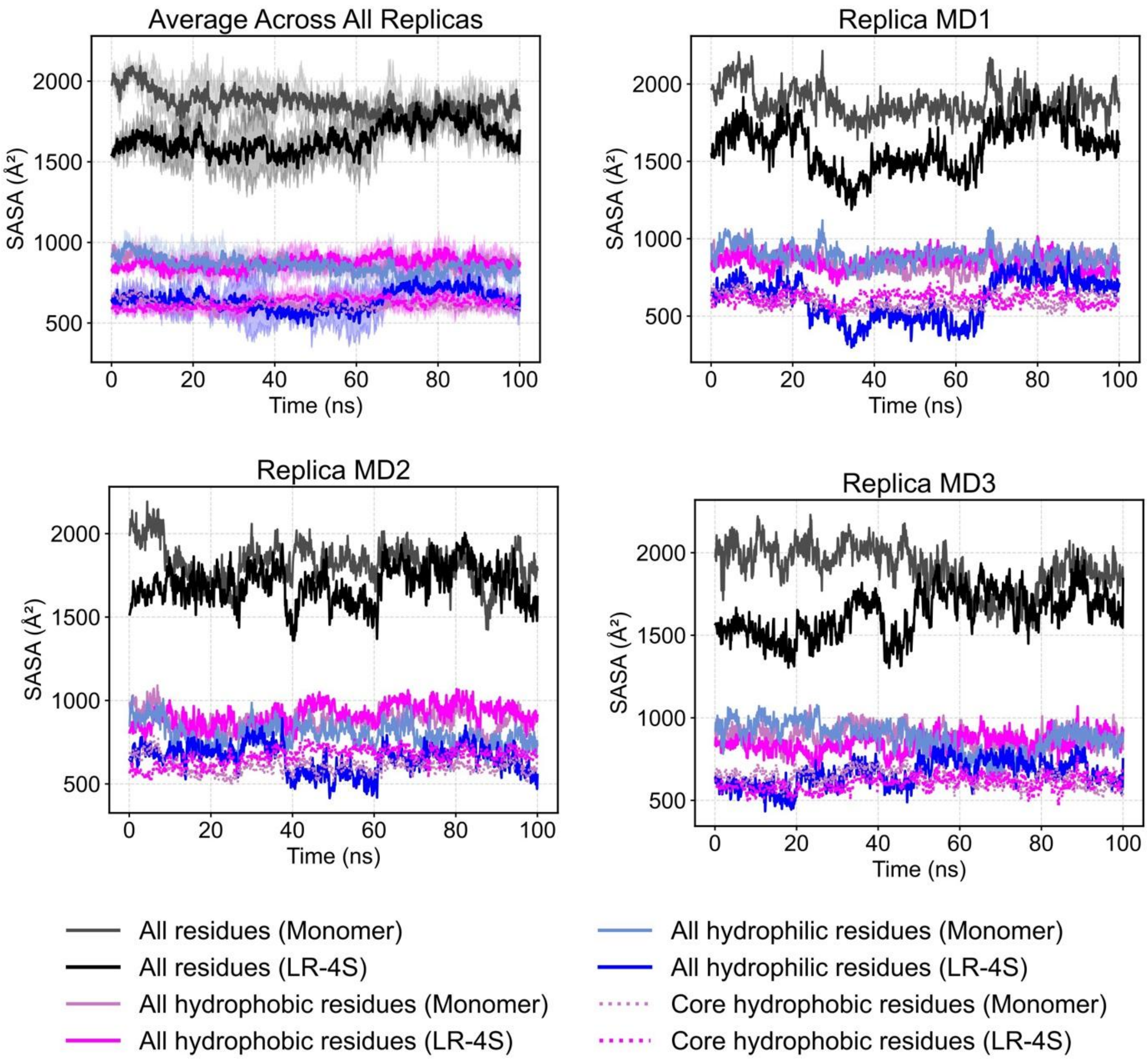

Figure S54. Solvent Accessible Surface Area (**SASA**) analysis of **αA66–80 peptide** interacting with **LR-4S**. The SASA profile of the αA66–80 peptide monomer is included as a faint reference for direct comparison. The plot shows average values across all replicas with corresponding standard deviations, followed by results from individual replicas.

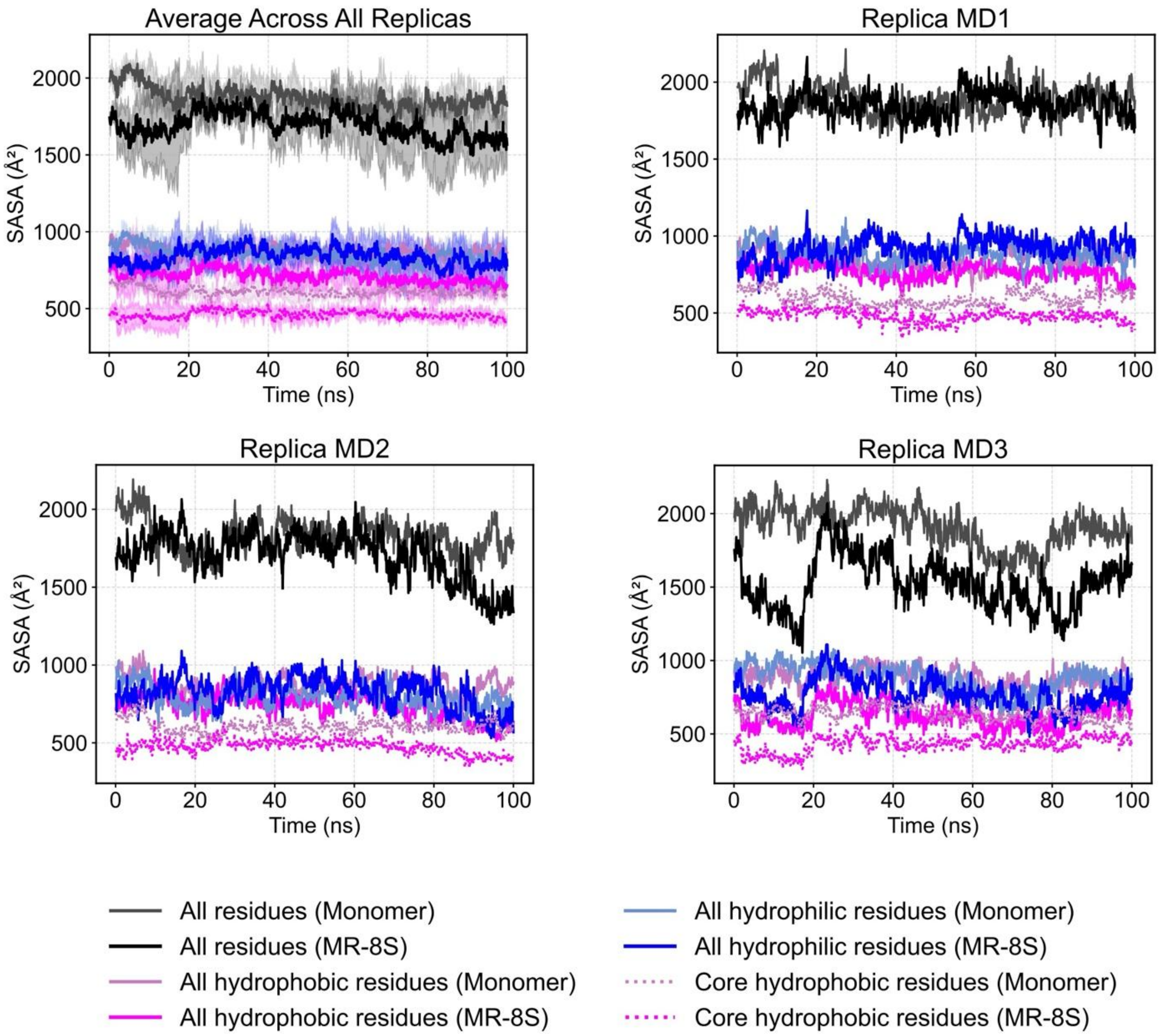

Figure S55. Solvent Accessible Surface Area (**SASA**) analysis of **αA66–80 peptide** interacting with **MR-8S**. The SASA profile of the αA66–80 peptide monomer is included as a faint reference for direct comparison. The plot shows average values across all replicas with corresponding standard deviations, followed by results from individual replicas.

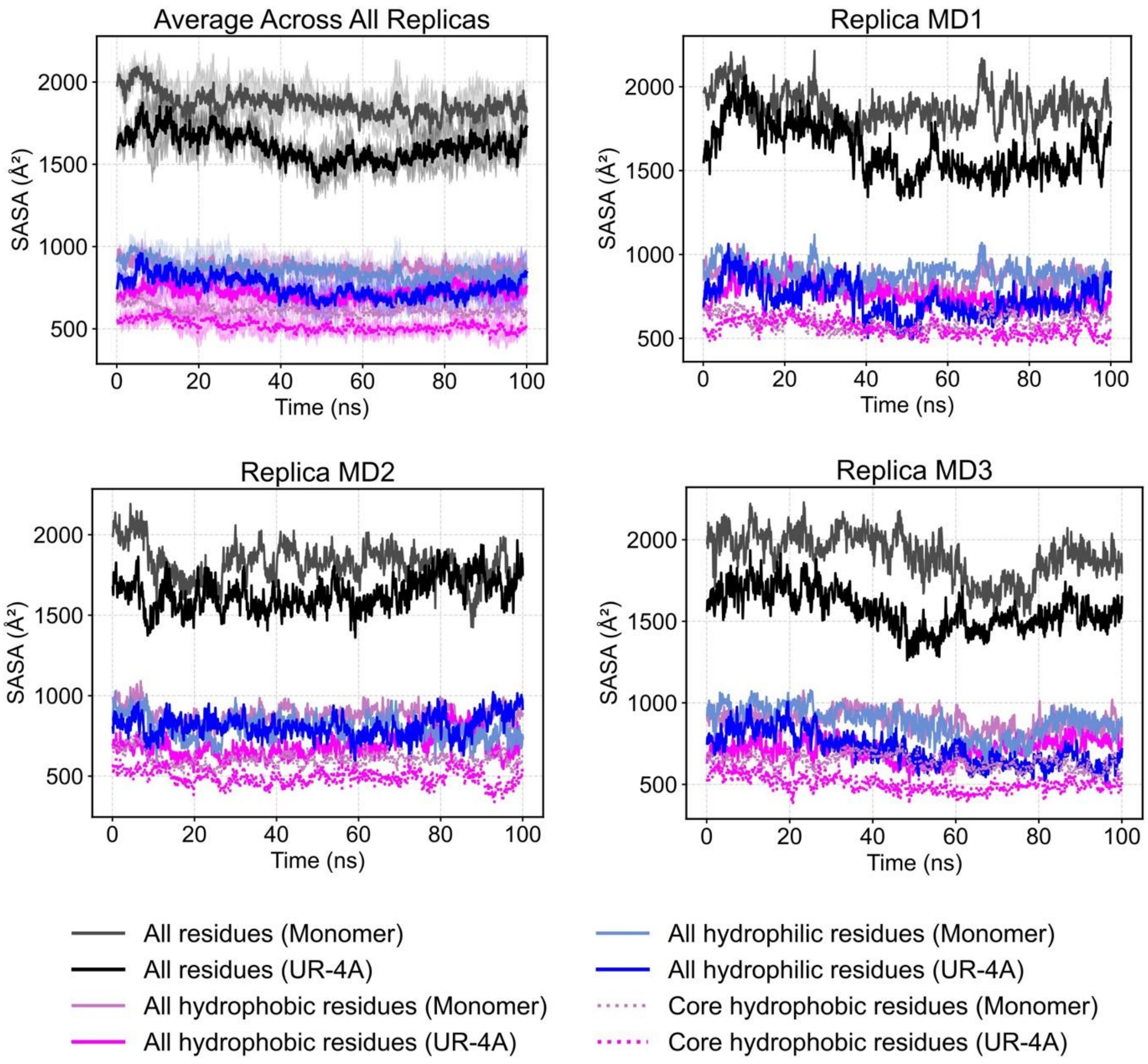

Figure S56. Solvent Accessible Surface Area (**SASA**) analysis of **αA66–80 peptide** interacting with **UR-4A**. The SASA profile of the αA66–80 peptide monomer is included as a faint reference for direct comparison. The plot shows average values across all replicas with corresponding standard deviations, followed by results from individual replicas.

XII.III Replica-wise lie analysis for αA66–80-Crystallin Peptide and Resorcinarene complex

The Linear Interaction Energy (lie) analysis reveals a clear distinction between electrostatic and van der Waals contributions to complex stabilization, See Figure S51, represents averages over three replicas, with individual replica data provided in Figures S52-S55. Electrostatic interactions are predominantly mediated by hydrophilic residues, whereas van der Waals stabilization primarily arises from hydrophobic residues.

Figure S51 presents a comparative lie analysis of αA66–80 peptide complexes with different resorcinarenes (**UR-4S**, **LR-4S**, **MR-8S**, and **UR-4A**).

For **UR-4S**, the overall van der Waals contribution is largely dominated by non-core hydrophobic residues such as F80 and V77, with minimal participation from the core hydrophobic residues (F71–L75). This disparity between the van der Waals profiles of core hydrophobic residues and total hydrophobic residues indicates incomplete core masking and reduced complex stability relative to the other systems.

Similarly, **LR-4S** exhibits interaction profiles in which both electrostatic and van der Waals contributions are governed primarily by hydrophilic contacts. The stronger stabilization from hydrophilic residues compared to hydrophobic ones reflects poor engagement of the aggregation-prone hydrophobic core, resulting in weak inhibitory stabilization.

In contrast, **MR-8S** displays pronounced van der Waals stabilization dominated by core hydrophobic residues. This strong core engagement leads to effective hydrophobic masking, making MR-8S the most stable and potent inhibitor within the series.

UR-4A exhibits a more balanced interaction profile, involving both hydrophilic and hydrophobic residues. However, its core hydrophobic residues remain only partially masked and contribute modestly to overall stabilization, yielding intermediate complex stability and inhibitory activity.

Overall, the combined RMSF, SASA, and lie analysis reveal a consistent trend: inhibitor potency and complex stability directly correlate with effective hydrophobic core masking and strong van der Waals stabilization from core hydrophobic residues. Among the tested systems, MR-8S forms the most stable and effectively masked complex, followed by UR-4A with intermediate stability, while UR-4S and LR-4S exhibit the weakest stabilization due to insufficient hydrophobic core engagement.

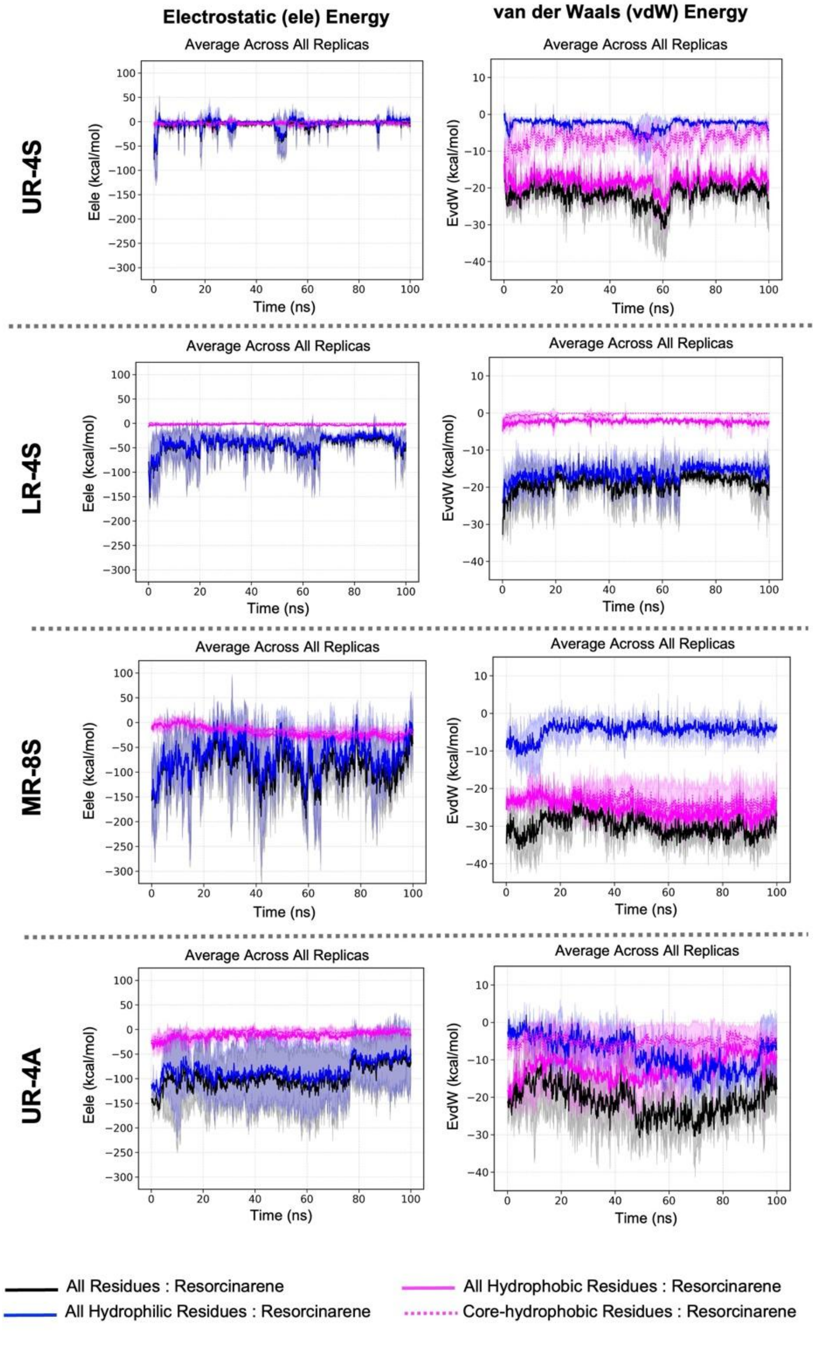

Figure S57. Comparative Linear Interaction (lie) analysis of **αA66–80 peptide** complexes with different resorcinarenes (**UR-4S**, **LR-4S**, **MR-8S** and **UR-4A**). The lie results decompose total interaction energy into electrostatic and van der Waals components, revealing distinct stabilization patterns driven by hydrophilic and hydrophobic residue interactions.

XII.II Replica-wise lie analysis for αA66–80-Crystallin Peptide and Resorcinarene complex

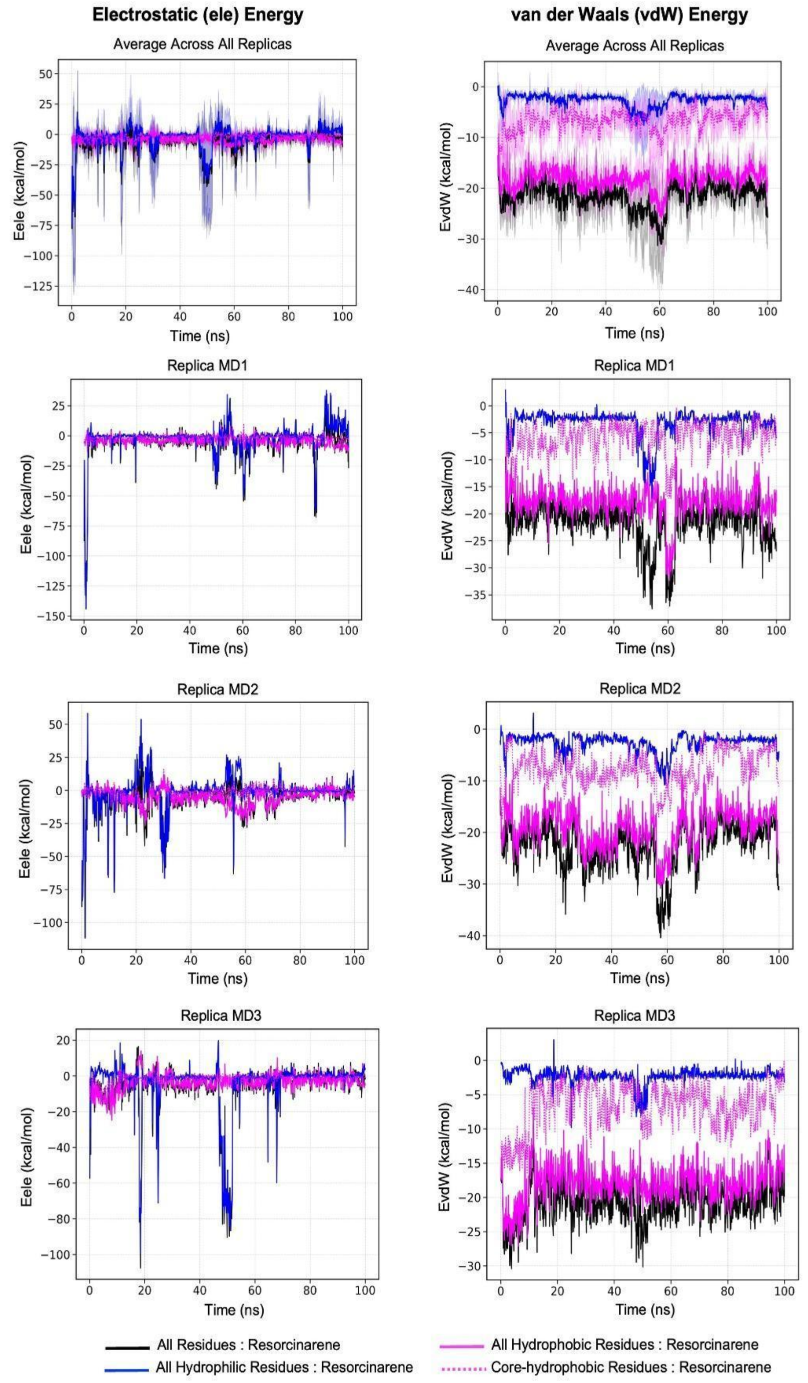

Figure S58. The linear interaction energy (**lie**) analysis both **electrostatic (Eele)** and **van der Waals (EvdW)** of the **αA66–80** peptide and **UR-4S** complex. The plot shows average values across all replicas with corresponding standard deviations, followed by results from individual replicas.

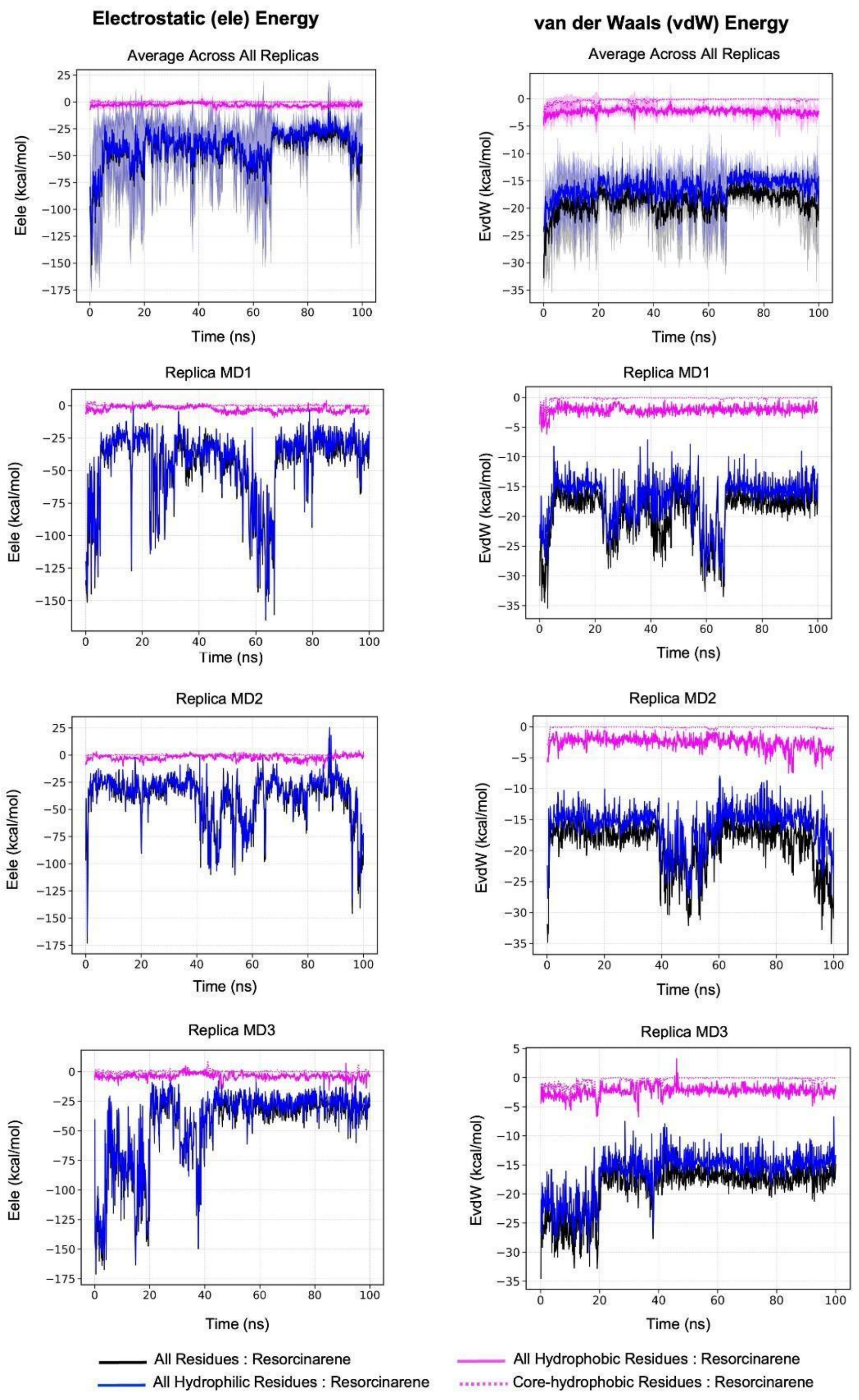

Figure S59. The linear interaction energy (**lie**) analysis both **electrostatic (Eele) and van der Waals (EvdW)** of the **αA66–80** peptide and **LR-4S** complex. The plot shows average values across all replicas with corresponding standard deviations, followed by results from individual replicas.

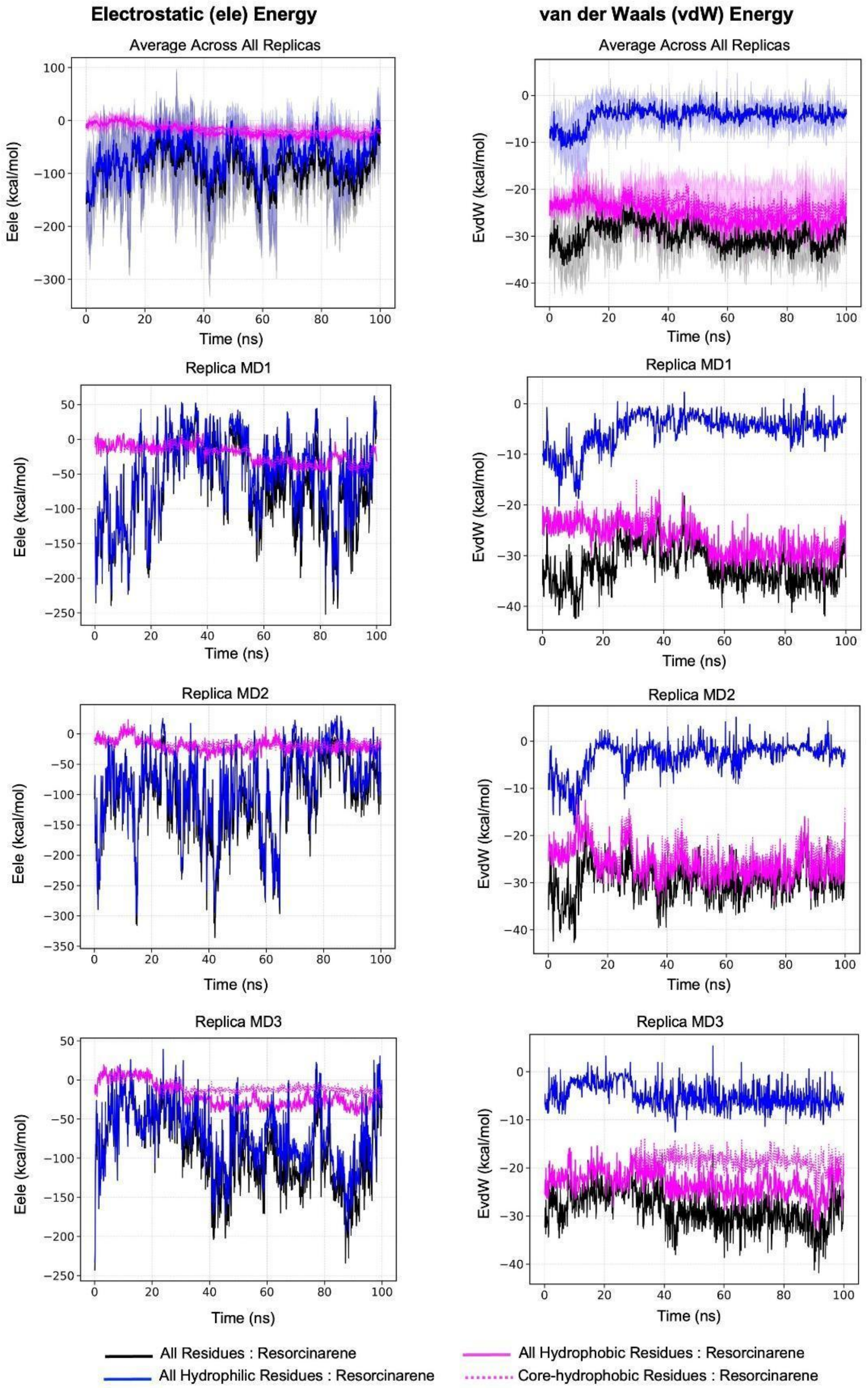

Figure S60. The linear interaction energy (**lie**) analysis both **electrostatic (Eele)** and **van der Waals (EvdW)** of the **αA66–80** peptide and **MR-8S** complex. The plot shows average values across all replicas with corresponding standard deviations, followed by results from individual replicas.

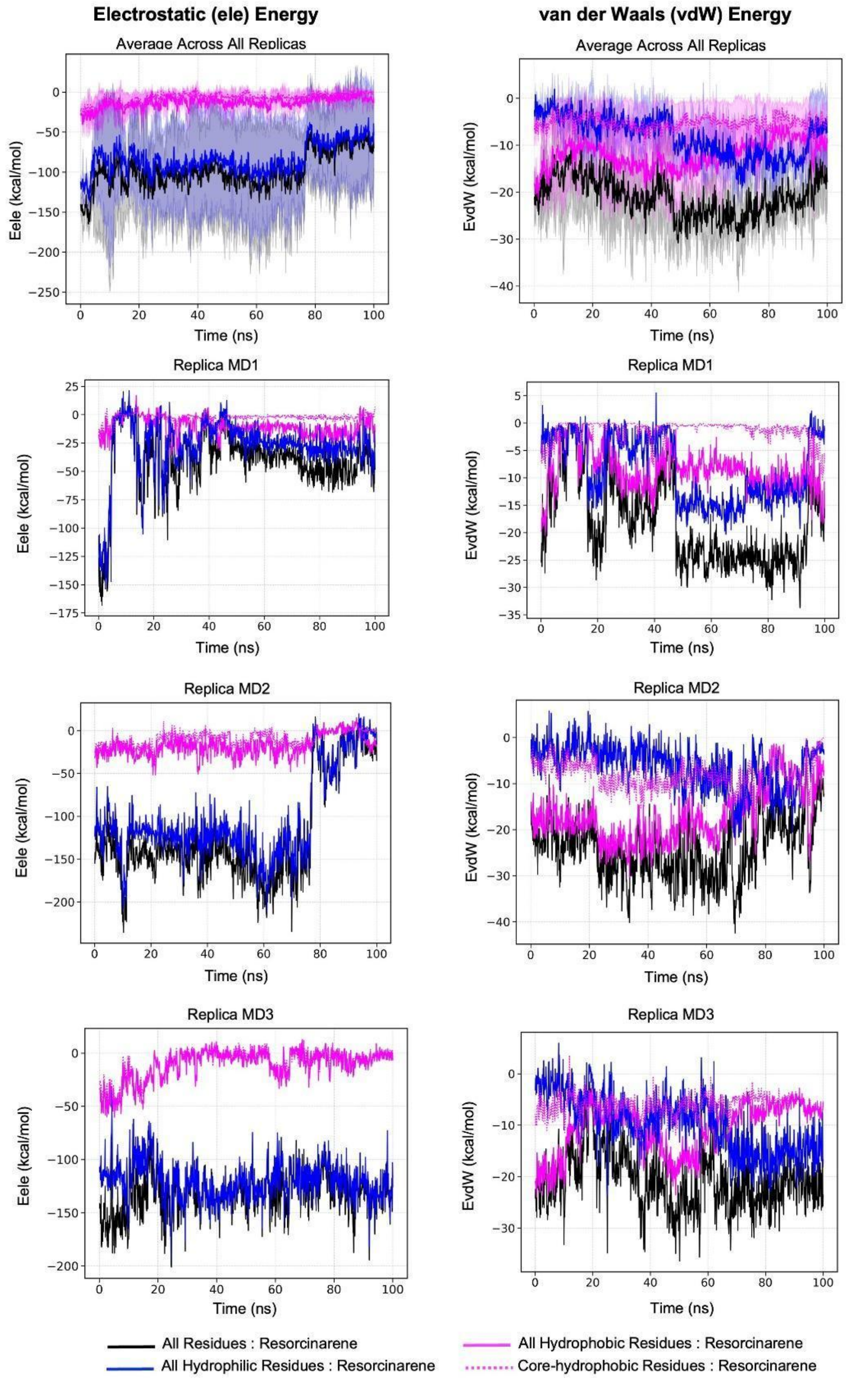

Figure S61. The linear interaction energy (**lie**) analysis both **electrostatic (Eele)** and **van der Waals (EvdW)** of the **αA66–80** peptide and **UR-4A** complex. The plot shows average values across all replicas with corresponding standard deviations, followed by results from individual replicas.

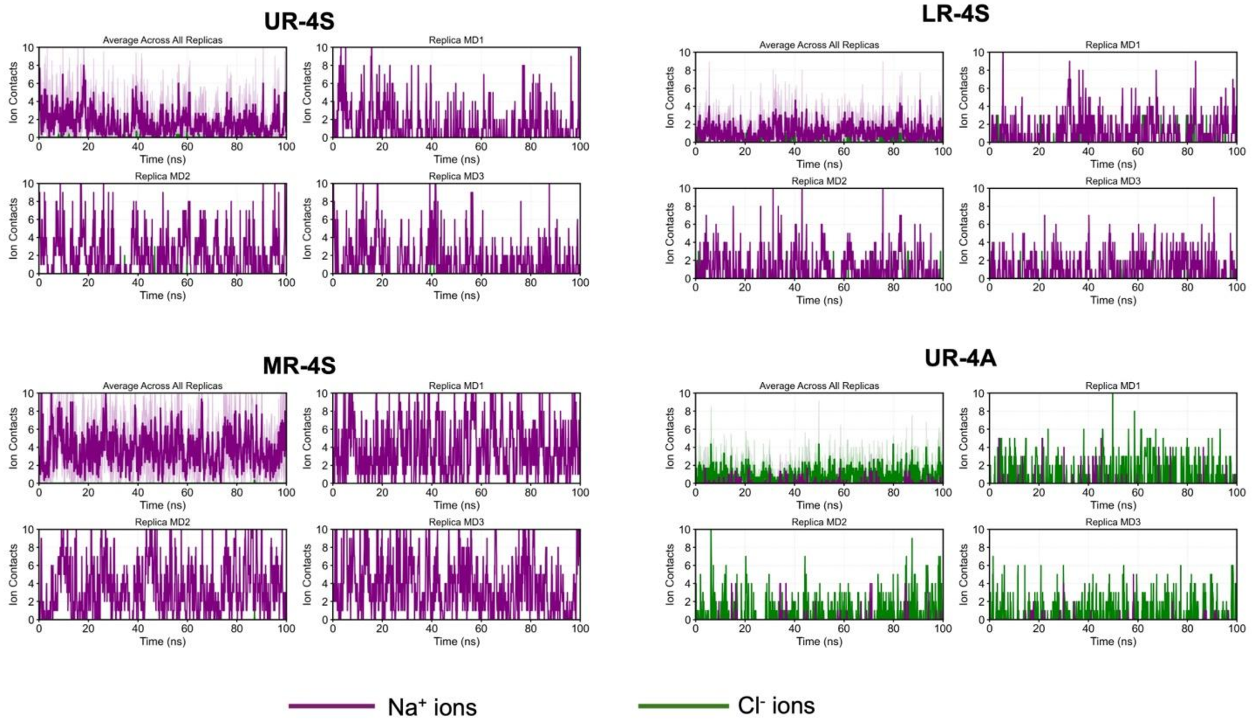

Figure S62. Time-resolved ion–macrocycle contact analysis for all resorcinarene–peptide complexes in the presence of 0.15 M NaCl. Average ion-contact profiles between Na^+/Cl^- ions and the resorcinarene systems (**UR-4S**, **LR-4S**, **MR-8S**, and **UR-4A**) are shown together with the corresponding replica-wise contact traces. Ion–macrocycle contacts were calculated using the cpptraj module[25] of AMBER24[8,9] with a 3.5 Å distance cutoff, where both native and nonnative contacts were included in the total contact count. The analysis reveals clear charge-dependent ion association behavior: the tetraanionic systems **UR-4S** and **LR-4S** coordinate approximately two Na^+ ions on average, the cationic **UR-4A** system coordinates approximately two Cl^- ions, and the octaanionic **MR-8S** system coordinates approximately four Na^+ ions. Ion interactions remain highly dynamic across all systems, indicating transient electrostatic screening rather than stable or persistent ion binding.

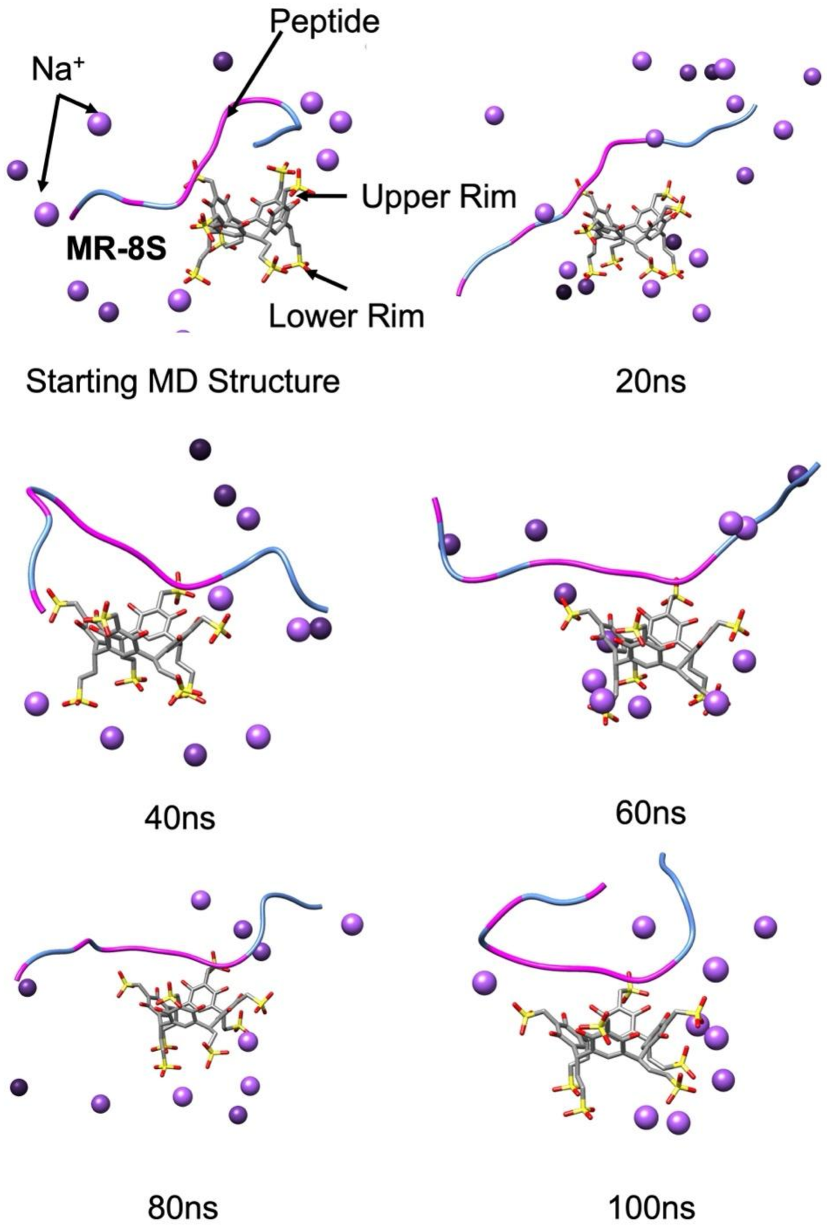

Figure S63. Representative snapshots illustrating the dynamic association of Na^+ ions with **MR-8S** over the course of the simulation (0, 20, 40, 60, 80, and 100 ns). Na^+ ions are observed to interact interchangeably with both the upper and lower rims of the macrocycle, without persistent localization. This dynamic ion distribution demonstrates that counterion association does not impose a fixed orientation on **MR-8S**, supporting the conclusion that macrocycle–peptide interactions, rather than ion binding, govern the observed binding mode.

XIII. Reversing the Fold by MR-8S

To investigate how **MR-8S** interferes with the aggregation of the αA66–80 peptide, we utilized pre-equilibrated dimeric and tetrameric assemblies obtained from 100 ns molecular dynamics simulations. These simulations yielded stable oligomeric structures, providing a reliable foundation to examine the impact of **MR-8S** on peptide aggregation and potential de-aggregation behavior. Representative dimer and tetramer structures were subsequently used to construct 2:1 and 4:1 peptide:MR-8S systems, respectively.

The selection of representative structures was based on a comprehensive clustering analysis performed across all three independent replicas for both dimer and tetramer systems. Specifically, all trajectories were combined and analyzed using clustering over a total of 3000 frames in Chimera[3]. The most populated cluster corresponded to compact oligomeric conformations characterized by strong packing interactions among the hydrophobic core residues. The centroid structure of this dominant cluster was therefore selected as the representative structure for both dimeric and tetrameric assemblies.

These representative structures were then used as targets for docking **MR-8S** using Smina[17]. The hydrophobic core region of chain A was defined as the primary binding site to evaluate the ability of **MR-8S** to disrupt key aggregation-driving interactions. The resulting docking poses are presented in Figure S64. The top-ranked docked complexes were subsequently subjected to further molecular dynamics simulations using the same protocol described earlier.

Dimer

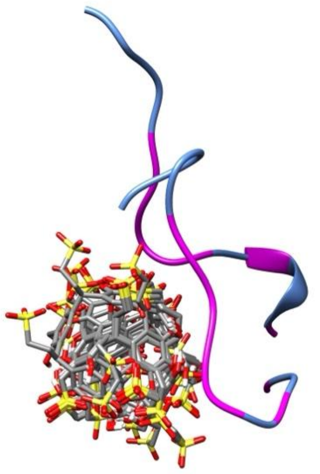

Dock pose	Binding Affinity/ kcal mol^{-1}
Dock 1	-5.1665
Dock 2	-5.1619
Dock 3	-5.1557
Dock 4	-5.1078
Dock 5	-5.0805
Dock 6	-5.0764
Dock 7	-5.07467
Dock 8	-5.0728
Dock 9	-5.0550

Tetramer

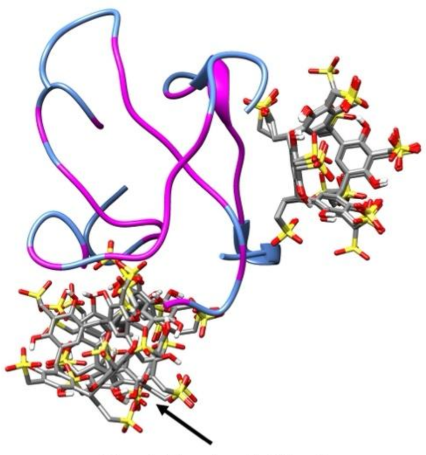

Dock pose	Binding Affinity/ kcal mol^{-1}
Dock 1	-5.8343
Dock 2	-5.8037
Dock 3	-5.7914
Dock 4	-5.7794
Dock 5	-5.6961
Dock 6	-5.6751
Dock 7	-5.6557
Dock 8	-5.4881
Dock 9	-5.4089

Figure S64. Docking poses of **MR-8S** on representative αA66–80 peptide oligomers. The representative dimer and tetramer structures, obtained from clustering analysis of combined MD trajectories, were used as targets for docking.

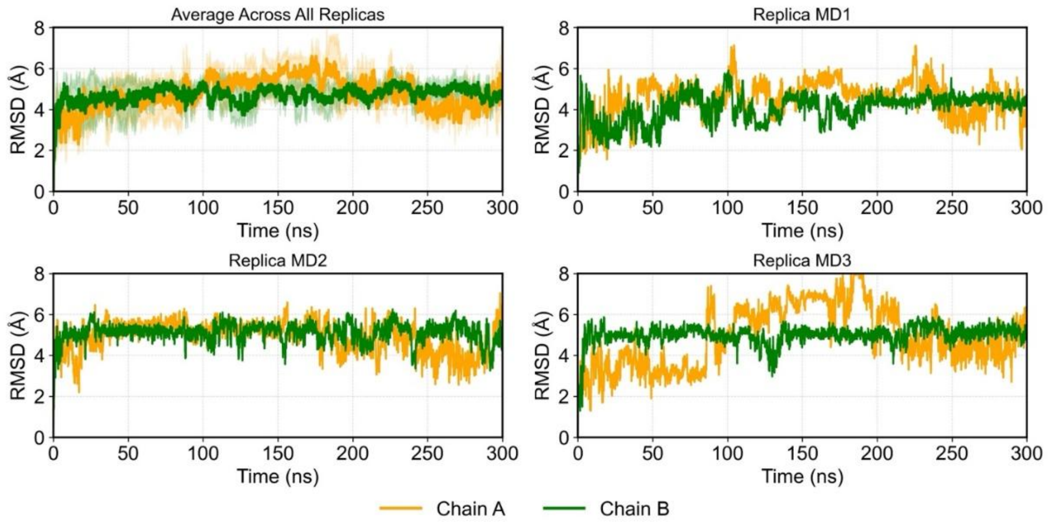

Figure S65. Root-mean-square deviation (**RMSD**) analysis relative to the initial simulation frame for the apo αA66–80 crystallin peptide **dimer** during the additional 200 ns extension of the simulations, resulting in a total simulation time of **300 ns**. The figure presents the average RMSD profile across three independent replicas with corresponding standard deviations, followed by the individual replica trajectories. The convergence of the RMSD profiles indicates that the dimeric assembly remains structurally stable and well equilibrated throughout the extended simulation timescale.

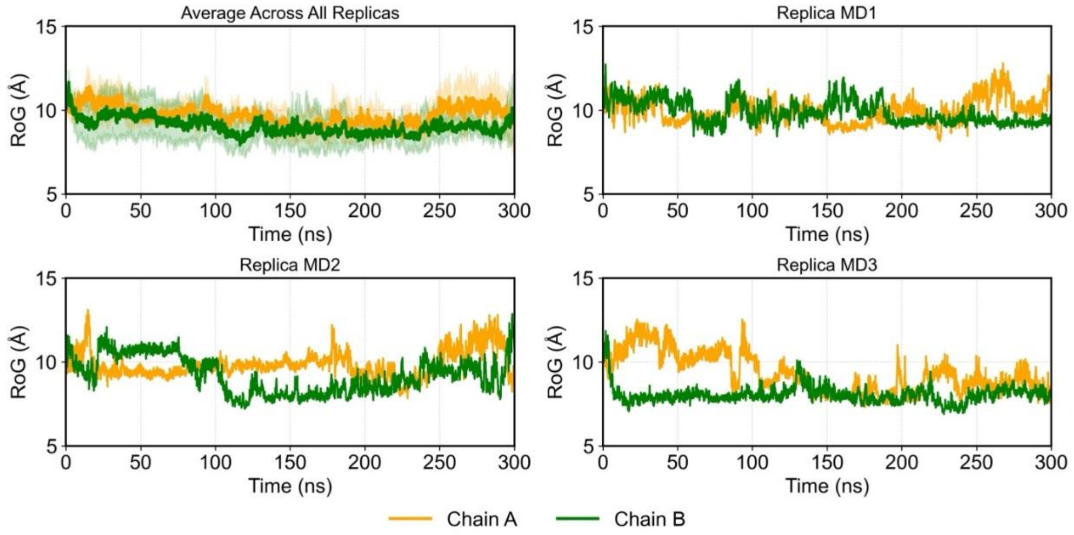

Figure S66. Radius of gyration (**RoG**) analysis of the apo αA66–80 crystallin peptide **dimer** during the additional 200 ns simulation extension (total simulation time: **300 ns**). The figure presents the average RoG profile across three independent replicas together with the corresponding standard deviations, followed by the individual replica trajectories. The relatively stable RoG values throughout the simulation indicate preservation of the compact aggregate structure and equilibration of the dimeric assembly over the extended timescale.

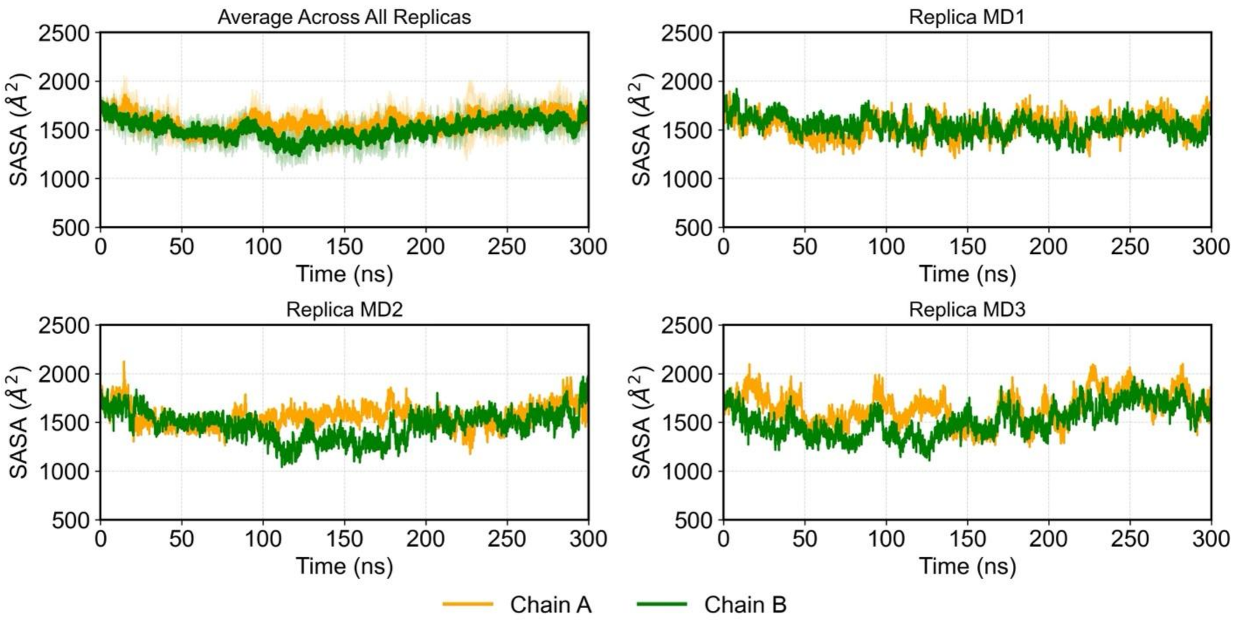

Figure S67. Solvent-accessible surface area (**SASA**) analysis of the apo αA66–80 crystallin peptide **dimer** during the additional 200 ns simulation extension (total simulation time: 300 ns). The figure shows the average SASA profile obtained from three independent replicas together with the corresponding standard deviations, followed by the individual replica trajectories. The absence of substantial changes in SASA throughout the simulation indicates maintenance of the aggregate structural integrity and equilibration of the dimeric assembly over the extended simulation timescale.

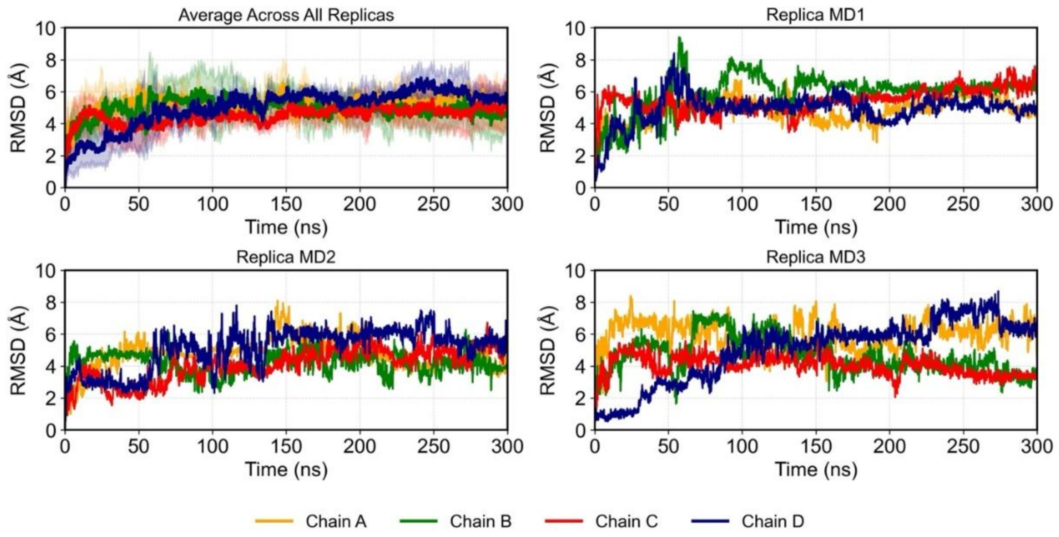

Figure S68. Root-mean-square deviation (**RMSD**) analysis relative to the initial simulation frame for the apo αA66–80 crystallin peptide **tetramer** during the additional 200 ns extension of the simulations, resulting in a total simulation time of **300 ns**. The figure presents the average RMSD profile across three independent replicas with corresponding standard deviations, followed by the individual replica trajectories. The convergence of the RMSD profiles indicates that the tetrameric assembly remains structurally stable and well equilibrated throughout the extended simulation timescale.

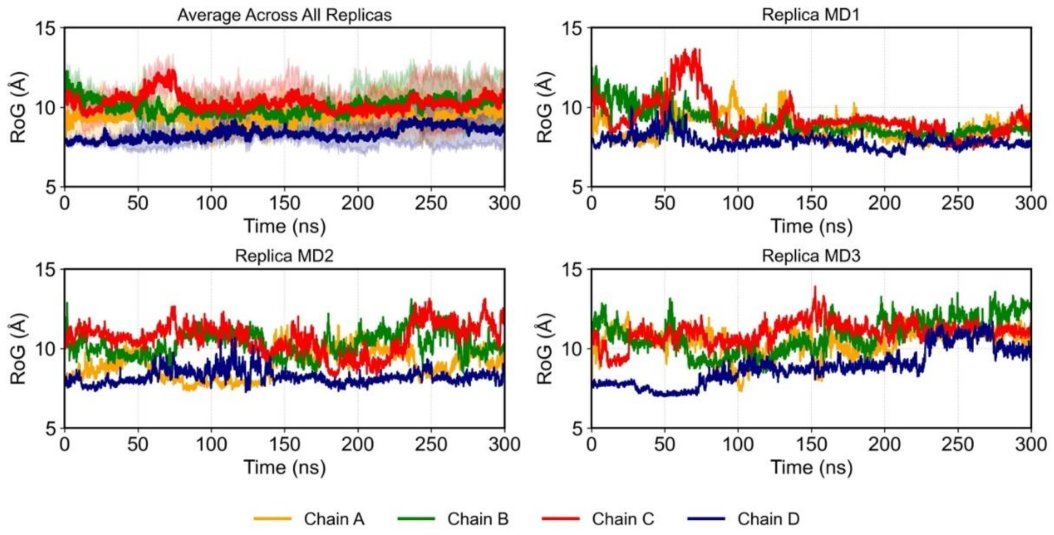

Figure S69. Radius of gyration (**RoG**) analysis of the apo αA66–80 crystallin peptide **tetramer** during the additional 200 ns simulation extension (total simulation time: **300 ns**). The figure presents the average RoG profile across three independent replicas together with the corresponding standard deviations, followed by the individual replica trajectories. The relatively stable RoG values throughout the simulation indicate preservation of the compact aggregate architecture and equilibration of the tetrameric assembly over the extended timescale.

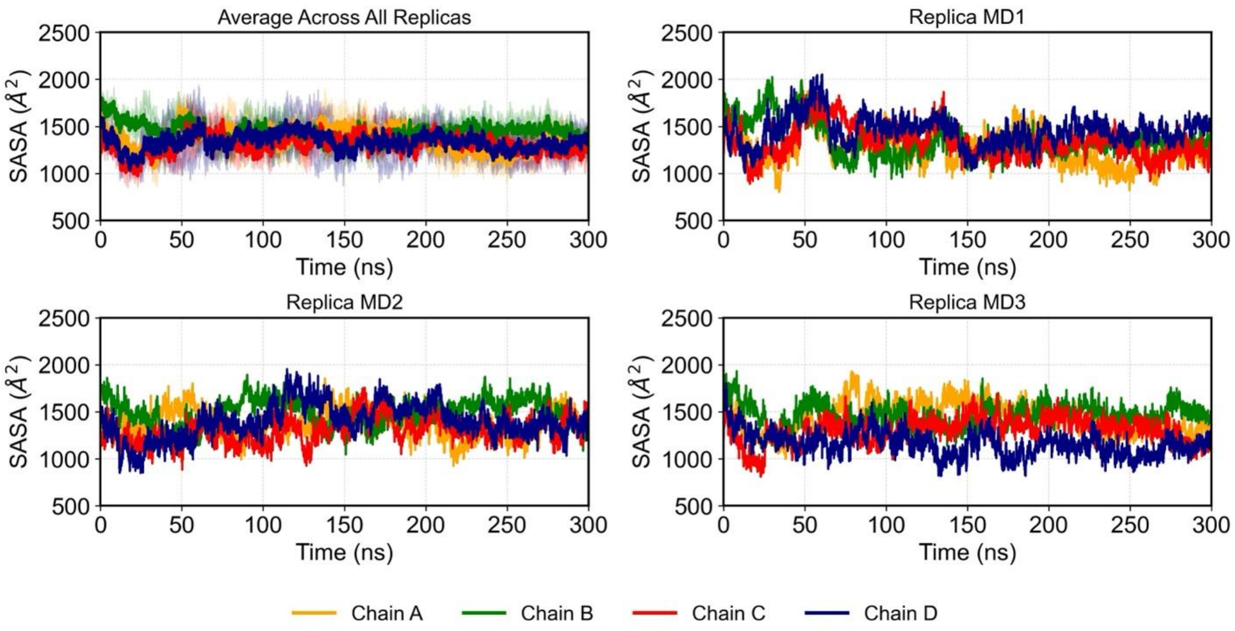

Figure S70. Solvent-accessible surface area (**SASA**) analysis of the apo αA66–80 crystallin peptide **tetramer** during the additional 200 ns simulation extension (total simulation time: **300 ns**). The figure shows the average SASA profile obtained from three independent replicas together with the corresponding standard deviations, followed by the individual replica trajectories. The absence of substantial changes in SASA throughout the simulation indicates maintenance of the tetrameric aggregate structural integrity and equilibration over the extended simulation timescale.

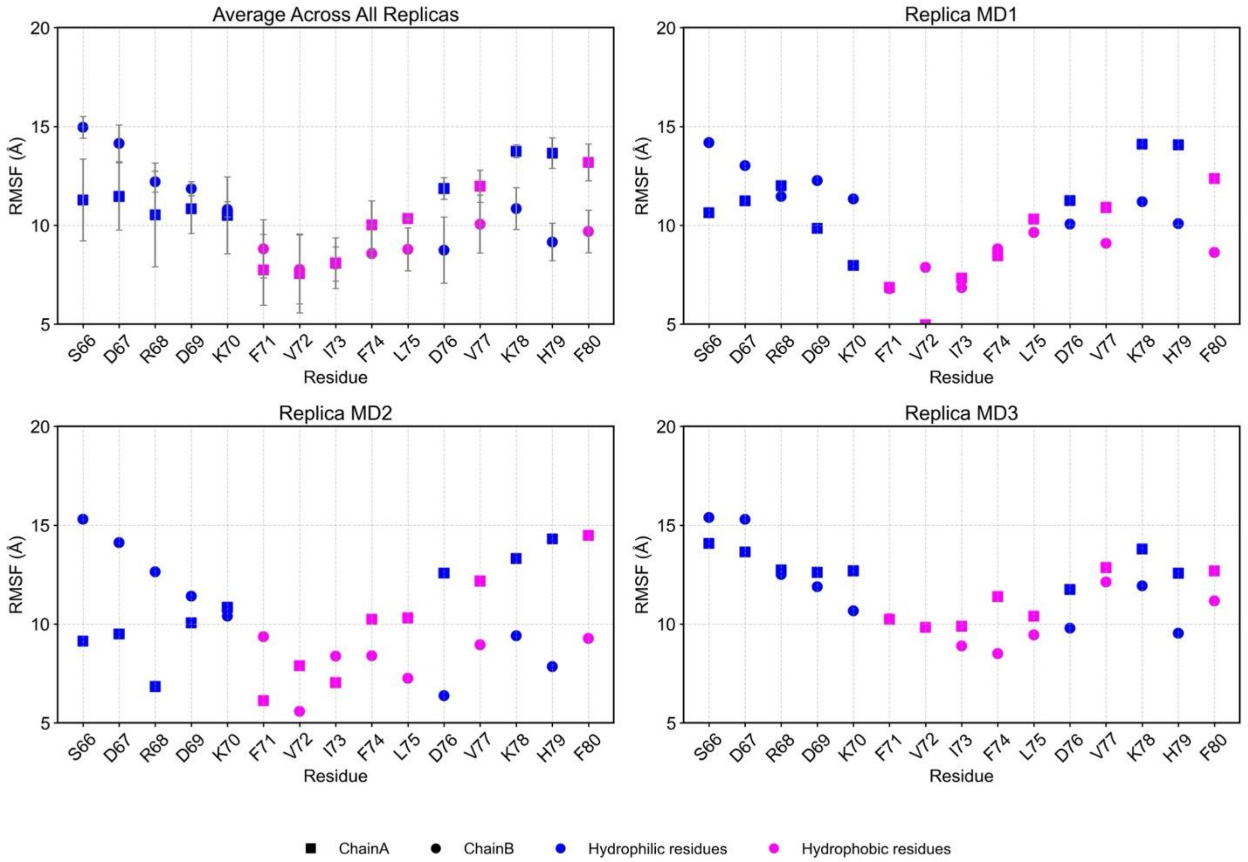

Figure S71. Root-Mean-Square-Fluctuation (**RMSF**) analysis of **αA66–80** crystallin peptide **dimer** formation over **300 ns**. The plot shows average values across all replicas with corresponding standard deviations, followed by results from individual replicas.

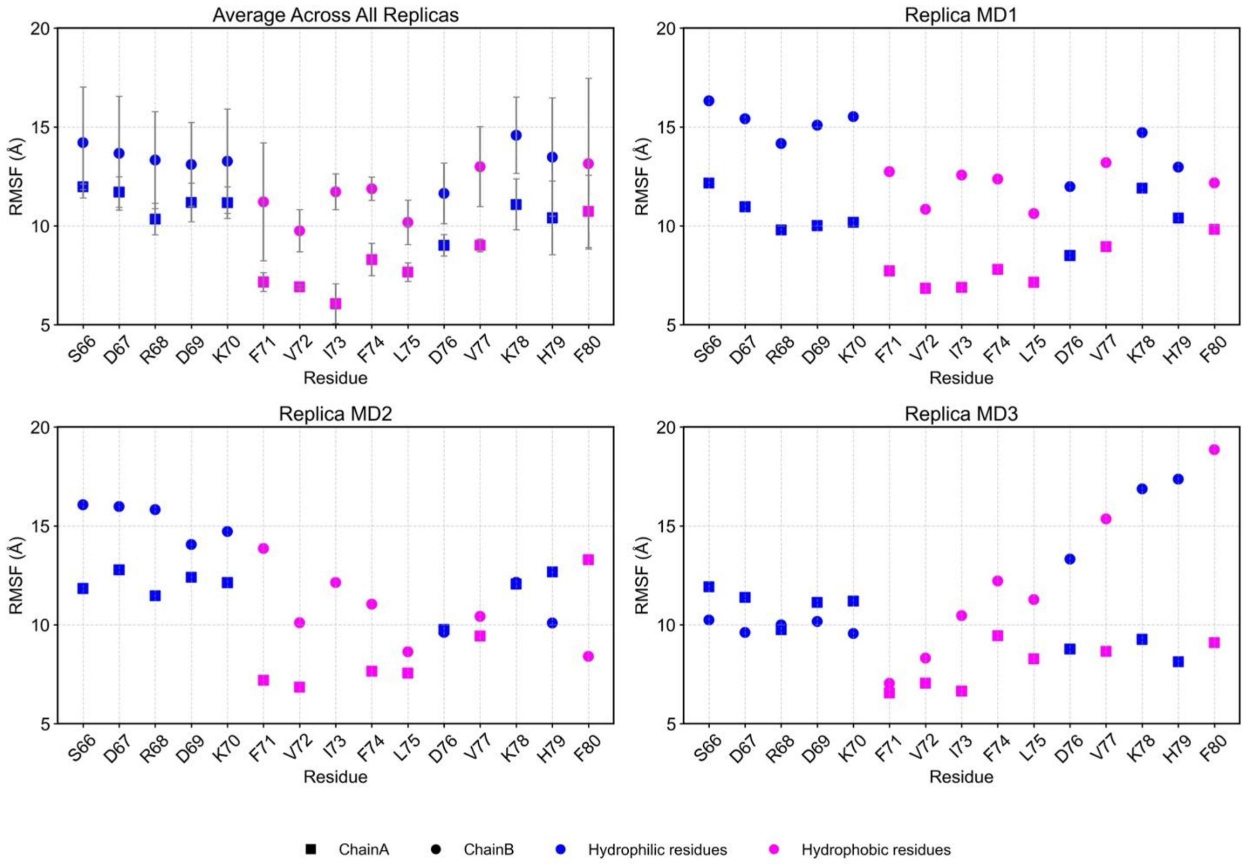

Figure S72. Root-mean-square fluctuation (**RMSF**) analysis of the **2:1 αA66–80 peptide:MR-8S** complex over **300 ns**. The plot shows average values across all replicas with corresponding standard deviations, followed by results from individual replicas.

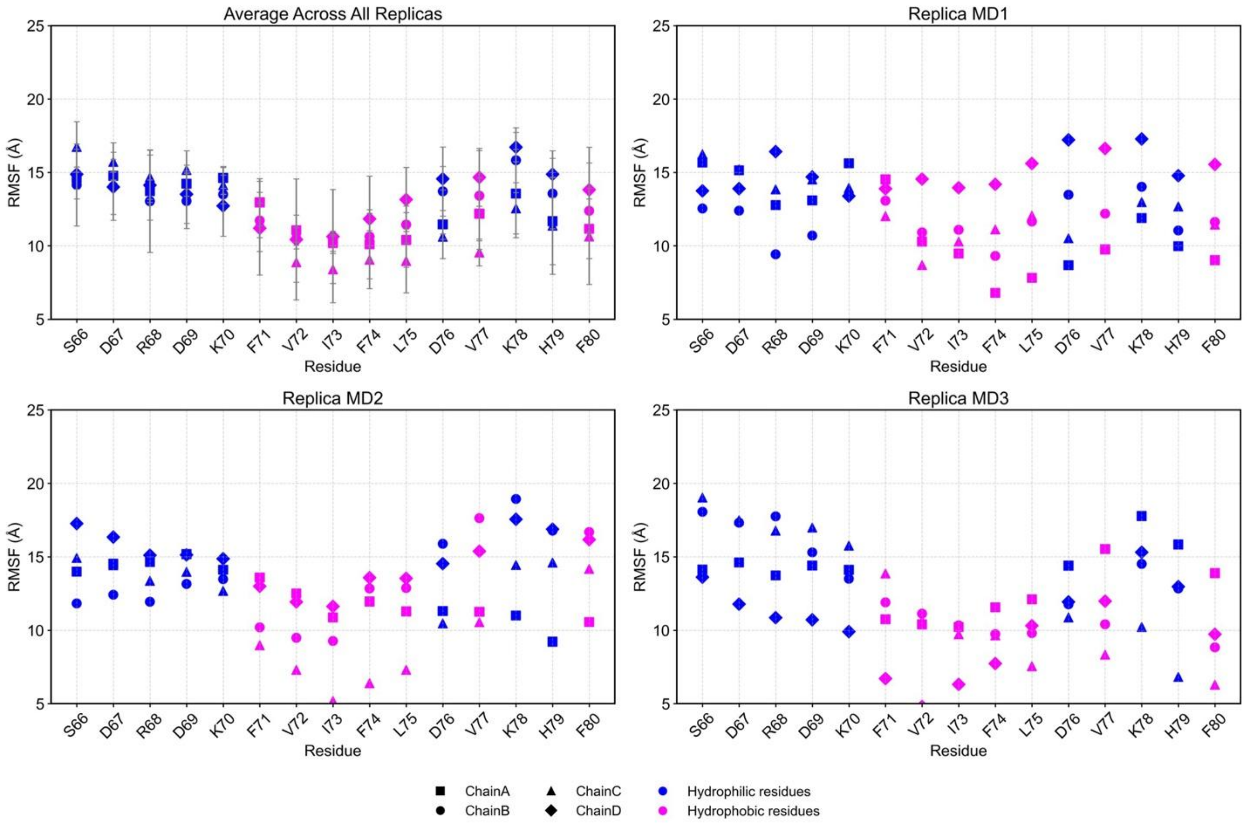

Figure S73. Root-Mean-Square-Fluctuation (**RMSF**) analysis of **αA66–80** crystallin peptide **tetramer** formation over **300 ns**. The plot shows average values across all replicas with corresponding standard deviations, followed by results from individual replicas.

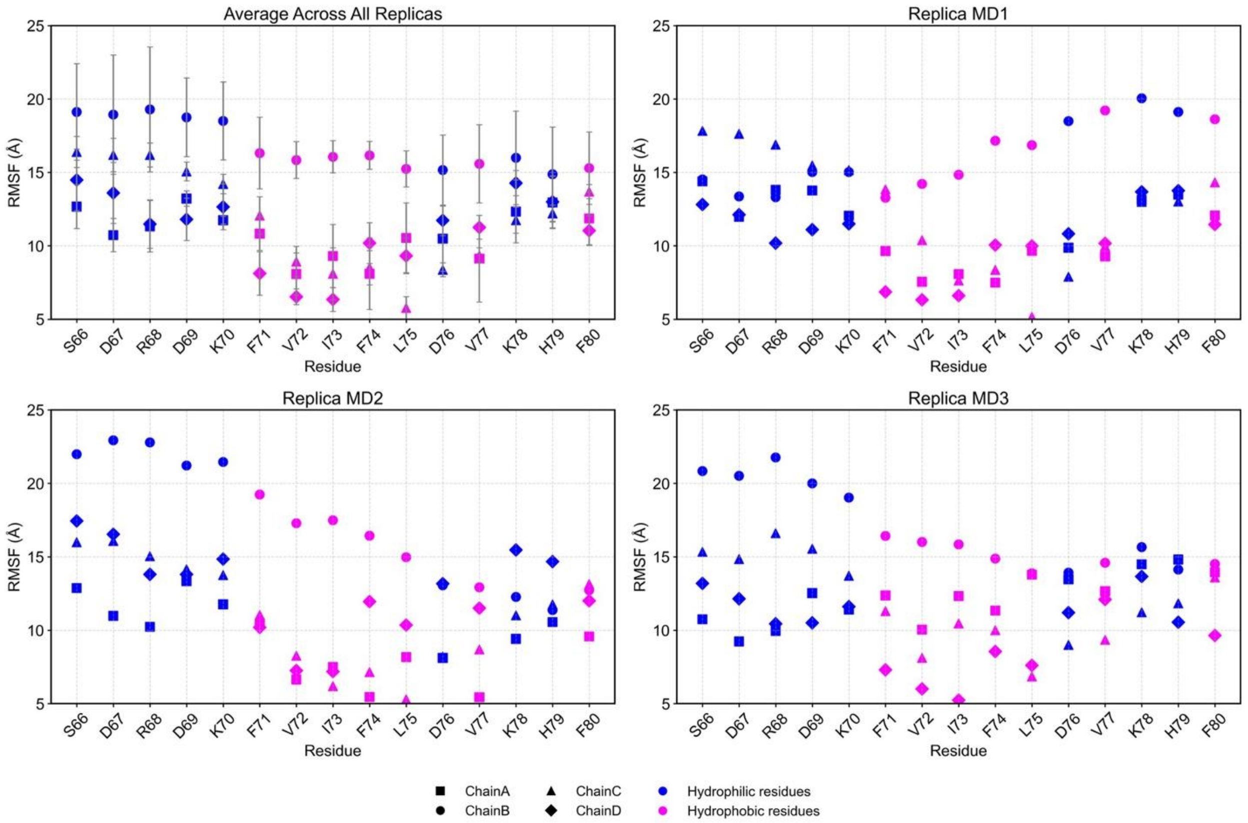

Figure S74. Root-mean-square fluctuation (**RMSF**) analysis of the **4:1 αA66–80 peptide:MR-8S** complex over **300 ns.** The plot shows average values across all replicas with corresponding standard deviations, followed by results from individual replicas.

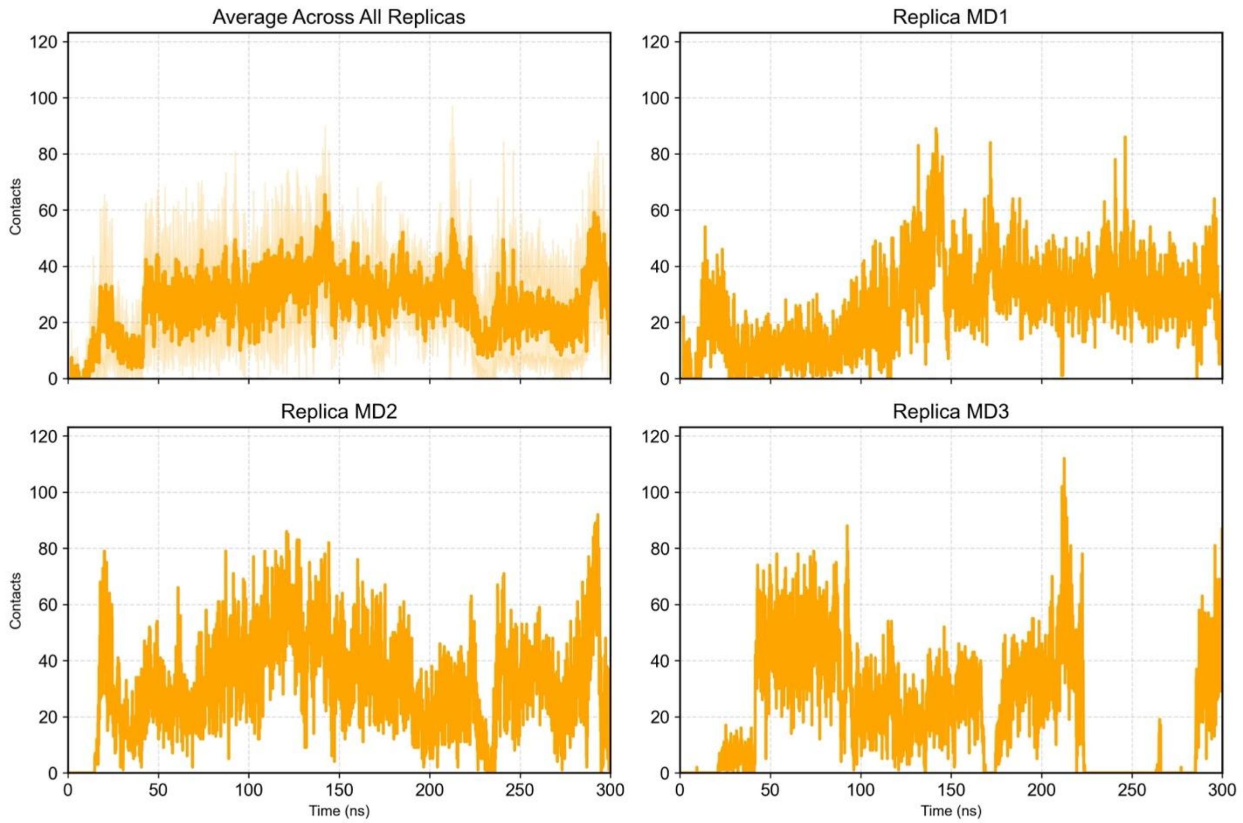

Figure S75. Time-resolved hydrophobic core **contact analysis** of the **dimeric αA66–80 peptide** assembly during the **300 ns** MD simulations. Inter-peptide contacts were monitored between chains A and B to evaluate the stability of the aggregation-prone hydrophobic core interactions. The plot presents the average contact profile across all replicas with corresponding standard deviations, followed by the individual replica trajectories

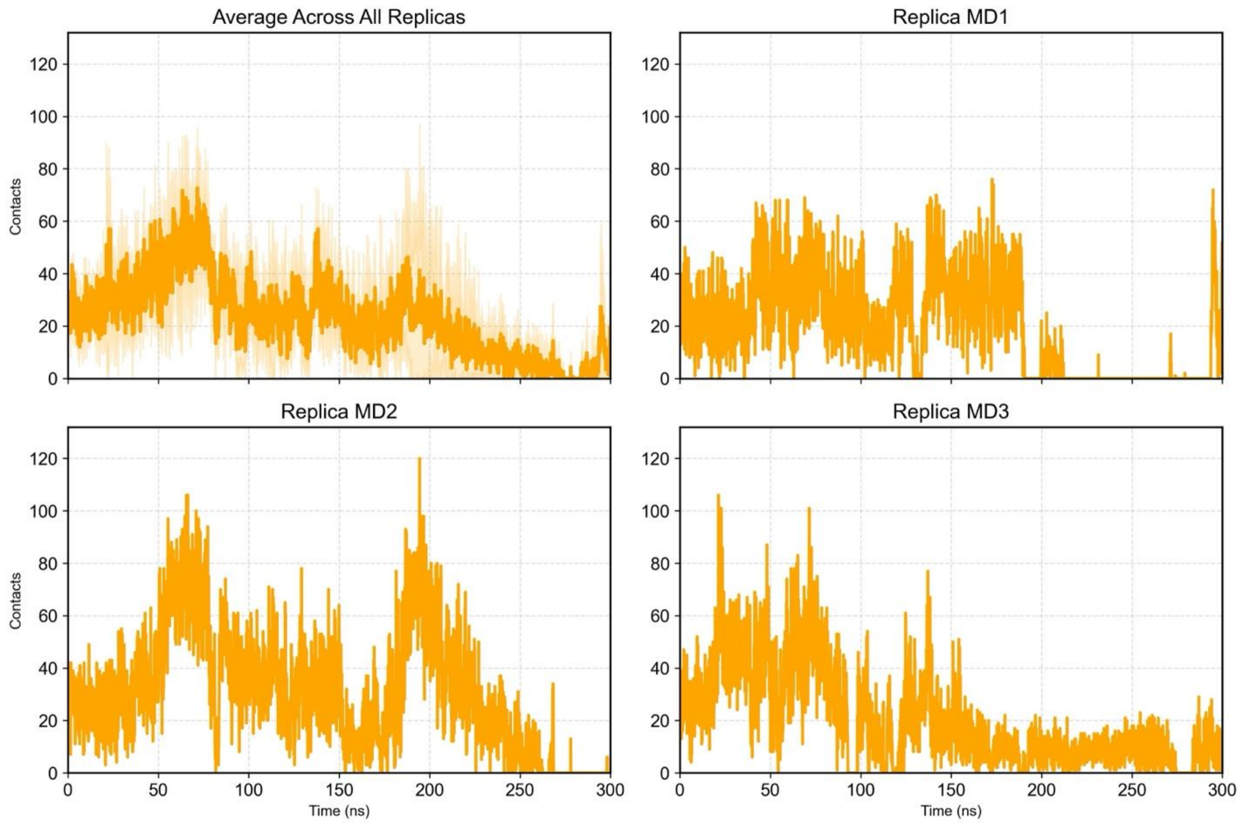

Figure S76. Time-resolved hydrophobic core **contact analysis** of the **2:1 αA66–80 peptide:MR-8S** assembly during the **300 ns** MD simulations. Inter-peptide contacts between chains A and B were monitored to evaluate the stability of aggregation-prone hydrophobic core interactions in the presence of **MR-8S**. The plot presents the average contact profile across all replicas with corresponding standard deviations, followed by the individual replica trajectories.

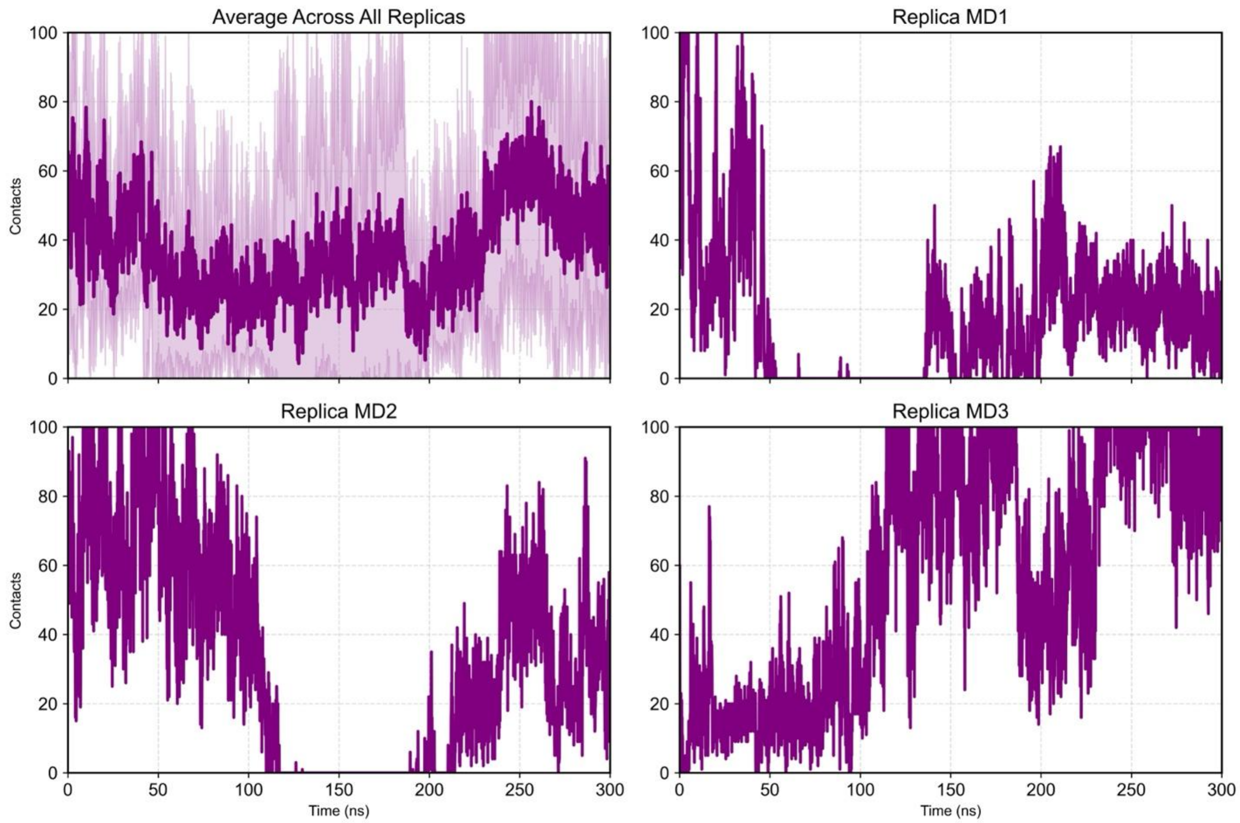

Figure 77. Time-resolved hydrophobic core **contact analysis** of the **tetrameric αA66–80** peptide assembly during the **300 ns** MD simulations. In the apo tetramer, inter-peptide contacts were analyzed between the A,B,C cluster and chain D to evaluate the stability of aggregation-prone hydrophobic core interactions. The plot presents the average contact profile across all replicas with corresponding standard deviations, followed by the individual replica trajectories.

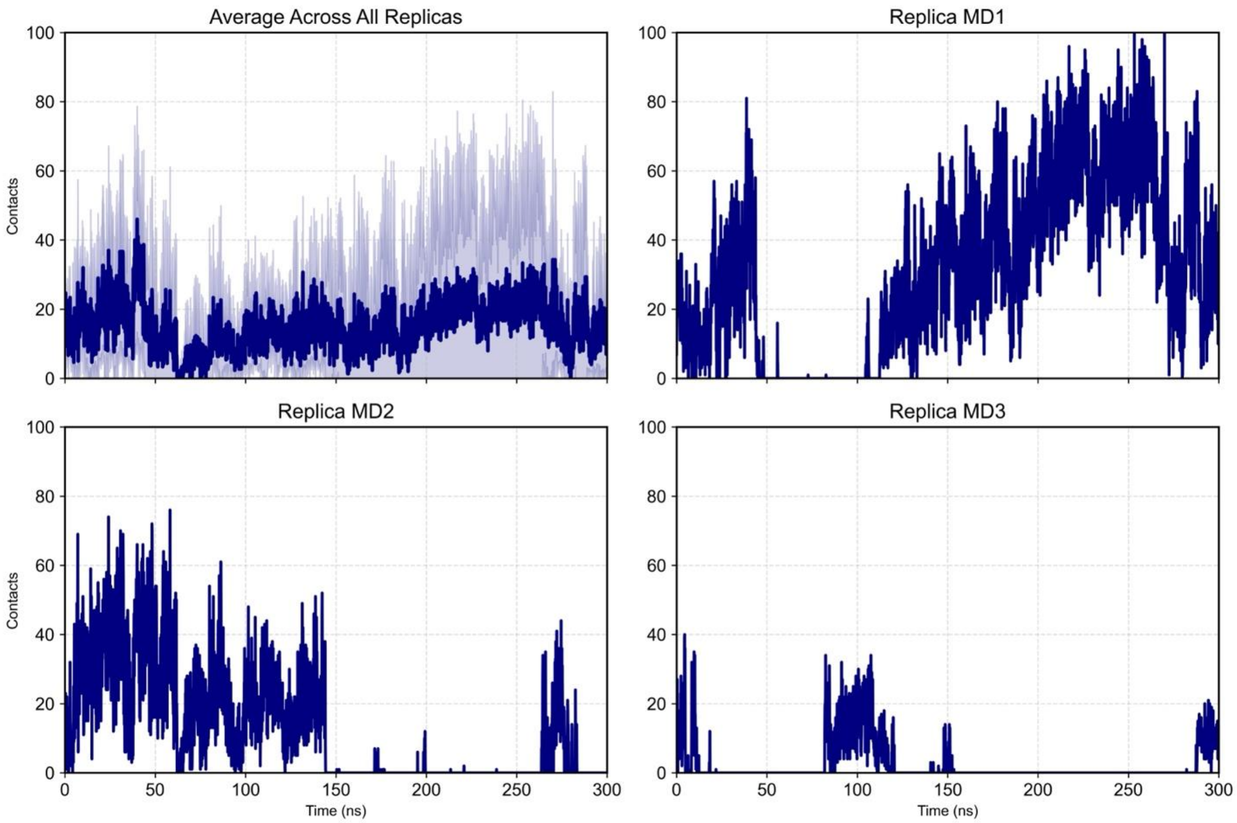

Figure 78. Time-resolved hydrophobic core **contact analysis** of the **4:1 αA66–80 peptide:MR-8S** assembly during the **300 ns** MD simulations. Inter-peptide contacts were monitored between the A,C,D cluster and chain B to evaluate the stability of aggregation-prone hydrophobic core interactions in the presence of **MR-8S**. The plot presents the average contact profile across all replicas with corresponding standard deviations, followed by the individual replica trajectories.

XIV. References

(1) Osei, F. B.; Twum, K.; Manfredi, B.; Fatohi, M.; Bessem Ojong, Y.; Washington, V.; Beyeh, N. K. Ionic Resorcinarenes as Drug Solubilization Agents in Water. *RSC Adv* **2024**, *14* (46), 34228–34238. https://doi.org/10.1039/D4RA06682K.

(2) Kaiser, C. J. O.; Peters, C.; Schmid, P. W. N.; Stavropoulou, M.; Zou, J.; Dahiya, V.; Mymrikov, E. V.; Rockel, B.; Asami, S.; Haslbeck, M.; Rappsilber, J.; Reif, B.; Zacharias, M.; Buchner, J.; Weinkauf, S. The Structure and Oxidation of the Eye Lens Chaperone AA-Crystallin. *Nat Struct Mol Biol* **2019**, *26* (12), 1141–1150. https://doi.org/10.1038/s41594-019-0332-9.

(3) Pettersen, E. F.; Goddard, T. D.; Huang, C. C.; Couch, G. S.; Greenblatt, D. M.; Meng, E. C.; Ferrin, T. E. UCSF Chimera - A Visualization System for Exploratory Research and Analysis. *J*

Comput Chem **2004**, *25* (13), 1605–1612. https://doi.org/10.1002/JCC.20084;WGROUP:STRING:PUBLICATION.

(4) Anandakrishnan, R.; Aguilar, B.; Onufriev, A. V. H++ 3.0: Automating PK Prediction and the Preparation of Biomolecular Structures for Atomistic Molecular Modeling and Simulations. *Nucleic Acids Res* **2012**, *40* (1), 537–541. https://doi.org/10.1093/NAR/GKS375.

(5) Myers, J.; Grothaus, G.; Narayanan, S.; Onufriev, A. A Simple Clustering Algorithm Can Be Accurate Enough for Use in Calculations of PKs in Macromolecules. *Proteins: Struct Funct Genet* **2006**, *63* (4), 928–938. https://doi.org/10.1002/PROT.20922.

(6) Gordon, J. C.; Myers, J. B.; Folta, T.; Shoja, V.; Heath, L. S.; Onufriev, A. H++: A Server for Estimating p Ka s and Adding Missing Hydrogens to Macromolecules. *Nucleic Acids Res* **2005**, *33* (2), 368–371. https://doi.org/10.1093/NAR/GKI464.

(7) Martinez, L.; Andrade, R.; Birgin, E. G.; Martínez, J. M. PACKMOL: A Package for Building Initial Configurations for Molecular Dynamics Simulations. *J Comput Chem* **2009**, *30* (13), 2157–2164. https://doi.org/10.1002/JCC.21224.

(8) Case, D. A.; Cerutti, D. S.; Cruzeiro, V. W. D.; Darden, T. A.; Duke, R. E.; Ghazimirsaeed, M.; Giambaşu, G. M.; Giese, T. J.; Götz, A. W.; Harris, J. A.; Kasavajhala, K.; Lee, T. S.; Li, Z.; Lin, C.; Liu, J.; Miao, Y.; Salomon-Ferrrer, R.; Shen, J.; Snyder, R.; Swails, J.; Walker, R. C.; Wang, J.; Wu, X.; Zeng, J.; Cheatham, T. E.; Roe, D. R.; Roitberg, A.; Simmerling, C.; York, D. M.; Nagan, M. C.; Merz, K. M. Recent Developments in Amber Biomolecular Simulations. *J Chem Inf Model* **2025**, *65* (15), 7835–7843. https://doi.org/10.1021/ACS.JCIM.5C01063.

(9) Case, D. A.; Aktulga, H. M.; Belfon, K.; Cerutti, D. S.; Cisneros, G. A.; Cruzeiro, V. W. D.; Forouzesh, N.; Giese, T. J.; Götz, A. W.; Gohlke, H.; Izadi, S.; Kasavajhala, K.; Kaymak, M. C.; King, E.; Kurtzman, T.; Lee, T. S.; Li, P.; Liu, J.; Luchko, T.; Luo, R.; Manathunga, M.; Machado, M. R.; Nguyen, H. M.; O'Hearn, K. A.; Onufriev, A. V.; Pan, F.; Pantano, S.; Qi, R.; Rahnamoun, A.; Risheh, A.; Schott-Verdugo, S.; Shajan, A.; Swails, J.; Wang, J.; Wei, H.; Wu, X.; Wu, Y.; Zhang, S.; Zhao, S.; Zhu, Q.; Cheatham, T. E.; Roe, D. R.; Roitberg, A.; Simmerling, C.; York, D. M.; Nagan, M. C.; Merz, K. M. AmberTools. *J Chem Inf Model* **2023**, *63* (20), 6183–6191. https://doi.org/10.1021/ACS.JCIM.3C01153.

(10) Jorgensen, W. L.; Chandrasekhar, J.; Madura, J. D.; Impey, R. W.; Klein, M. L. Comparison of Simple Potential Functions for Simulating Liquid Water. *J Chem Phys* **1983**, *79* (2), 926–935. https://doi.org/10.1063/1.445869.

(11) Tian, C.; Kasavajhala, K.; Belfon, K. A. A.; Raguette, L.; Huang, H.; Migues, A. N.; Bickel, J.; Wang, Y.; Pincay, J.; Wu, Q.; Simmerling, C. Ff19SB: Amino-Acid-Specific Protein Backbone Parameters Trained against Quantum Mechanics Energy Surfaces in Solution. *J Chem Theory Comput* **2019**, *16* (1), 528–552. https://doi.org/10.1021/ACS.JCTC.9B00591.

(12) Maier, J. A.; Martinez, C.; Kasavajhala, K.; Wickstrom, L.; Hauser, K. E.; Simmerling, C. Ff14SB: Improving the Accuracy of Protein Side Chain and Backbone Parameters from Ff99SB. *J Chem Theory Comput* **2015**, *11* (8), 3696–3713. https://doi.org/10.1021/ACS.JCTC.5B00255.

(13) Becke, A. D. Density-functional Thermochemistry. III. The Role of Exact Exchange. *J. Chem. Phys.* **1993**, *98*, 5648–5652. https://doi.org/ 10.1063/1.464913.

(14) Hariharan, P. C.; Pople, J. A. Accuracy of AH equilibrium geometries by single determinant molecular-orbital theory, *Mol. Phys.* **1974**, *27*, 209-14. DOI: 10.1080/00268977400100171.

(15) Francl, M.M.; Pietro, W. J.; Hehre, W. J.; Binkley, J. S.; DeFrees, D. J.; Pople, J. A.; Gordon, M. S. Self-Consistent Molecular Orbital Methods. 23. A polarization-type basis set for 2nd-row elements, *J. Chem. Phys.* **1982**, *77*, 3654-65. DOI: 10.1063/1.444267.

(16) Frisch, M. J.; Trucks, G. W.; Schlegel, H. B.; Scuseria, G. E.; Robb, M. A.; Cheeseman, J. R.; Scalmani, G.; Barone, V.; Petersson, G. A.; Nakatsuji, H.; et. al. Gaussian 16, Revision C.01; Gaussian Inc: Wallingford CT, 2016.

(17) Koes, D. R.; Baumgartner, M. P.; Camacho, C. J. Lessons Learned in Empirical Scoring with Smina from the CSAR 2011 Benchmarking Exercise. *J Chem Inf Model* **2013**, *53* (8), 1893–1904. https://doi.org/10.1021/CI300604Z.

(18) O'Boyle, N. M.; Banck, M.; James, C. A.; Morley, C.; Vandermeersch, T.; Hutchison, G. R. Open Babel: An Open Chemical Toolbox. *J Cheminform* **2011**, *3* (1), 42-50. https://doi.org/10.1186/1758-2946-3-33.

(19) Wang, J.; Wolf, R. M.; Caldwell, J. W.; Kollman, P. A.; Case, D. A. Development and testing of a general amber force field. *J. Comput. Chem.* **2004**, *25*, 1157−1174. https://doi.org/10.1002/jcc.20035.

(20) Götz, A. W.; Williamson, M. J.; Xu, D.; Poole, D.; Le Grand, S.; Walker, R. C. Routine Microsecond Molecular Dynamics Simulations with AMBER on GPUs. 1. Generalized Born. *J Chem Theory Comput* **2012**, *8* (5), 1542–1555. https://doi.org/10.1021/CT200909J.

(21) Salomon-Ferrer, R.; Götz, A. W.; Poole, D.; Le Grand, S.; Walker, R. C. Routine Microsecond Molecular Dynamics Simulations with AMBER on GPUs. 2. Explicit Solvent Particle Mesh Ewald. *J Chem Theory Comput* **2013**, *9* (9), 3878–3888. https://doi.org/10.1021/CT400314Y.

(22) Ryckaert, J. P.; Ciccotti, G.; Berendsen, H. J. C. Numerical Integration of the Cartesian Equations of Motion of a System with Constraints: Molecular Dynamics of n-Alkanes. *J Comput Phys* **1977**, *23* (3), 327–341. https://doi.org/10.1016/0021-9991(77)90098-5.

(23) Loncharich, R. J.; Brooks, B. R.; Pastor, R. W. Langevin Dynamics of Peptides: The Frictional Dependence of Isomerization Rates of N-acetylalanyl-N′-methylamide. *Biopolymers* **1992**, *32* (5), 523–535. https://doi.org/10.1002/BIP.360320508.

(24) Berendsen, H. J. C.; Postma, J. P. M.; Van Gunsteren, W. F.; Dinola, A.; Haak, J. R. Molecular Dynamics with Coupling to an External Bath. *J Chem Phys* **1984**, *81* (8), 3684–3690. https://doi.org/10.1063/1.448118.

(25) Roe, D. R.; Cheatham, T. E. PTRAJ and CPPTRAJ: Software for Processing and Analysis of Molecular Dynamics Trajectory Data. *J Chem Theory Comput* **2013**, *9* (7), 3084–3095. https://doi.org/10.1021/CT400341P.

***
